# Parasites of the hermit crab *Pagurus hirsutiusculus*; distribution, prevalence, and thermal ecology

**DOI:** 10.1371/journal.pone.0335145

**Published:** 2025-11-19

**Authors:** Madeleine H. Abbott, Christopher D.G. Harley, Harmony A. Martell, Carly Janusson, Matthew A. Lemay, Alyssa-Lois M. Gehman

**Affiliations:** 1 Department of Zoology, University of British Columbia, Vancouver, British Columbia, Canada; 2 Department of Biology, University of Victoria, Victoria, British Columbia, Canada; 3 Institute for the Ocean and Fisheries, University of British Columbia, Vancouver, British Columbia, Canada; 4 Hakai Institute, Quadra Island, British Columbia, Canada; 5 Institute of Environment, Florida International University, Miami, Florida, United States of America; IEAPM: Instituto de Estudos do Mar Almirante Paulo Moreira, BRAZIL

## Abstract

Parasites are common throughout the biosphere and can play significant ecological roles. However, most parasites are understudied, particularly with regards to how their prevalence and impacts vary with environmental conditions. As a result, there remains an incomplete understanding of how both parasites and their hosts may be impacted by climate change. We conducted field surveys to better understand the parasite distributions of the intertidal hermit crab *Pagurus hirsutiusculus* in British Columbia*.* We found three genera of externally visible parasites (*Eremitione giardi, Peltogasterella* sp., and *Peltogaster* sp.), and one hyperparasite (*Liriopsis pygmaea*), which were present throughout the surveyed region. The prevalence of *E. giardi* was related to salinity while *Peltogaster* sp. was related to host size. At some locations, we observed a decline in prevalence in the parasite *Peltogaster* sp. following the 2021 Pacific Northwest heat wave event. In the lab, we compared the metabolic rate across a temperature range and survivorship post heat stress in individuals infected with *Peltogaster* sp. and uninfected individuals. We did not find a difference in metabolism based on infection status but found that uninfected individuals had significantly higher survivorship following heat stress. This study highlights the pervasiveness of parasitic interactions and demonstrates the need to study them in combination with environmental factors to better understand the effects of climate change on populations.

## Introduction

Parasite infections are ubiquitous in biological systems [1] and can have strong ecological and physiological effects. They can regulate host populations by altering reproductive ability [2], and account for as much as 78% of food web linkages in some ecosystems [3]. Despite their ubiquity and importance, parasites are frequently left out of ecological studies. Even our understanding of parasite taxonomy is woefully poor; it is estimated that many parasite species (e.g., up to 95% of helminths) remain undescribed due to their small and inconspicuous nature [1]. Identifying the parasites present in a community is a critical first step to understanding their ecology and role in a changing environment.

Host-parasite interactions can mediate host response outcomes to warming [4–6], and ignoring the important interactions between host-parasite dynamics and abiotic stressors could result in erroneous conclusions about the risks of climate change on a species or community [7,8]. In the context of changing ocean conditions, it is important to understand parasite natural history in order to determine what regions are likely to contain specific parasites and to understand how their prevalence might be affected by climate change [4]. A variety of abiotic (e.g., salinity, temperature) and biotic factors (e.g., host size, host density) have been suggested as possible factors associated with prevalence in some marine parasite systems [4,9–12], but it is not known how widely these apply across marine ecosystems. Abiotic and biotic factors can also influence the relationship between parasites and their hosts; parasites can affect their hosts in a myriad of ways including altering thermal tolerance, mortality, reproduction, and growth [4,13,14]. This information is important in determining how host parasite dynamics might change with warming temperatures, and how these changes might affect population structure. For example, infection by the parasitic barnacle, *Loxothylacus panopaei* (Gissler, 1884) [15,16]*,* will likely decline in response to climate change because of an offset in the thermal performance and tolerance between infected and uninfected hosts [4]. As the changes to host-parasite dynamics under climate change are expected to be complex [17], it is important to explore this topic across systems.

One of the challenges of incorporating parasites into ecological studies is that they are often small and hidden inside their hosts [1]. Externally visible parasites, such as rhizocephalans, are more easily quantified, and therefore make excellent study systems. The hermit crab *Pagurus hirsutiusculus* (Dana, 1851) [18,19] has been documented to have multiple externally visible parasites [20]. *Pagurus hirsutiusculus* is common in intertidal habitats throughout the west coast of North America, from California to Alaska [21]. Despite its high abundance and wide geographic spread, its parasites have not been well studied. The parasite species that have been previously recorded associated with *P. hirsutiusculus* include the rhizocephalan *Peltogaster paguri* Rathke, 1842 [22,23]*,* the colonial rhizocephalan *Peltogasterella gracilis* (Boschma 1927) [24,25]*,* and the bopyrid isopod *Eremitione giardi* (Calman, 1898) [20,26–30]. (S1 Fig). According to a review of the literature (S1 Methods and S2 Fig), rhizocephalans identified as *Peltogaster paguri* are distributed across much of the temperate northern hemisphere. However, the taxonomic identifications for *Peltogaster paguri* and *Peltogasterella gracilis* in BC and Oregon are based on morphological similarities to taxa from other ocean basins, and DNA barcoding (see Results section) suggests that taxonomic revision may be required. DNA Barcoding provides a powerful tool for species identification and can be especially useful for differentiating cryptic species that lack clear morphological differences and provide evidence for previously undescribed diversity in groups that require more taxonomic attention [31,32]. We will conservatively refer to each as *Peltogaster* sp. and *Peltogasterella* sp. within this manuscript.

One of the reasons *P. hirsutiusculus* and its externally visible parasites is such a compelling system to work in is that it allows for comparison of effects based on parasite life strategy. *Peltogaster* sp. is a rhizocephalan, which is a highly specialized parasitic barnacle with an internal root system and external reproductive portion known as an externa [14]. The lifecycle of these parasites includes dispersive larval stages and several stages of invasion of a single host [33]. These parasites absorb nutrition using their extensive root system and can manipulate the host’s endocrine and nervous systems [14]. In contrast, the isopod parasite found on *P. hirsutiusculus* (*Eremitione giardi*) is an ectoparasite and lives within the host’s branchial chamber where it feeds on hemolymph [34]. Its lifecycle is quite different compared to rhizocephalans, and involves an intermediate host (likely a copepod) and two separate free-living larval stages [34]. Because of this increased complexity, it is more difficult to understand prevalence fluctuations or make climate change predictions as there are more steps in its lifecycle that could contribute to any patterns seen. There have been field studies of *P. paguri* prevalence in Alaska [28] but other parasite records from this region consist of simple occurrence data [35,36]. There has yet to be a thorough investigation of the parasites present in hermit crabs in coastal British Columbia.

Coastal British Columbia experienced a record-breaking heat event in 2021, with air temperature anomalies reaching up to 20°C above normal [37]. These extreme temperatures caused mass mortality in many intertidal invertebrate species in the Salish Sea [38]. This event also provided an opportunity to examine how parasite prevalence in hermit crabs may be altered by future warming. As *P. hirsutiusculus* lives in the intertidal zone, they are already exposed to a wide range of temperatures over a relatively short period of time [21,39]. For example, a tidepool in BC on a hot day in the summer may reach a temperature of 32°C over the course of a tide [39]. Prior work on the thermal tolerance of *P. hirsutiusculus* [40], indicates that they may already experience stress near the edge of their tolerance levels in BC tidepools. Rhizocephalans in other systems have been shown to alter host thermal tolerance [4], but this has yet to be examined in *Pagurus hirsutiusculus*.

Given the general scarcity of detailed information on the distribution and prevalence of parasites in this host, the potential factors associated with those patterns, and the important but often overlooked relationship between global change and host-parasite dynamics, we have identified two main objectives for our research:

1. Determine the distribution and prevalence of rhizocephalan and bopyrid isopod parasites *Pagurus hirsutiusculus* in British Columbia (BC) in relation to potential related factors;
2. Explore the effect of *Peltogaster* sp. infection on host thermal performance and heat-related mortality.

To better understand parasite distribution and prevalence, we conducted field surveys to test the hypothesis that salinity, relative host abundance, and host size are associated with parasite prevalence. We predicted that the prevalence of parasites will be greater in high salinity environments than in low salinity environments based on previous evidence indicating that low salinity can be a refuge from rhizocephalan parasites [12], and salinity has been previously found positively correlated with prevalence of some bopyrid isopods [41]. We also expected to see a positive relationship between host relative abundance and parasite prevalence, as higher host density increases the likelihood of an individual coming into contact with a parasitized host or the transmission stage of the parasite, as has been observed generally [9] and in isopods [10]. Maximum density of rhizocephalans has also been found to be correlated with host density [42]. We predicted a higher rate of parasitization in larger crabs due to the probable older age of larger hosts resulting in a greater chance of eventually becoming parasitized which has been seen in rhizocephalans [11,42] and some bopyrid isopod species [43]. To understand the effect of parasites on *P. hirsutiusculus*, we examined how temperature affects the metabolic rate of hosts infected by *Peltogaster* sp., as well as how infection affects post-heat stress mortality. We predicted that infected crabs would have a lower metabolic rate at high temperatures compared to uninfected crabs, as a result of the potential increased energy burden from the parasite [44] which could make it more difficult for the host to maintain metabolic function under thermal stress. We also expected this to be reflected in higher mortality of infected individuals post experiment. This aligns with findings that other rhizocephalans have been shown to lower host thermal tolerance [4].

## Methods

### Field distribution methods

We surveyed *Pagurus hirsutiusculus* at 65 sites on the southwestern and central coasts of British Columbia, Canada, to document the presence of parasites, and determined the prevalence of parasitized hosts at 56 of those sites (S1 Table). We conducted timed searches (totaling up to 40 minutes) at each site to determine relative hermit crab abundance. We conducted searches at low tides of ≤ 1.5m that took place from the water line to approximately 2.5-3m above Canadian chart datum, suitable substrate dependent. We searched for hermit crabs in crevasses on bedrock and under rocks in cobbled areas as we found it to be the typical habitat for the species. Following collection, we examined crabs for external parasites by waiting for them to begin to emerge from their shell, and then gently pulling them out by hand to view their abdomen and carapace, exposing at least half their abdomen. This was sufficient to observe potential rhizocephalan externa as well whether the crabs were ovigerous. However, this method does not detect hermit crabs with internal infections, or early stage bopyrid parasitizations that lack a bulging carapace, so will likely result in some false negatives.

To estimate the size of the host, we measured the length of the left dactyl on their second walking leg to nearest 0.1 mm using calipers, which can be used as a proxy for overall body size [45]. We released hermit crabs after the measurements. We also took salinity samples at each site at a depth of 10–30 cm to determine whether salinity is associated with parasite prevalence. We measured the salinity in the lab using a refractometer (Atago S/Mill-E). We selected five sites for repeated surveys through time to better understand potential seasonal prevalence changes (S1 Table, S1 Methods). Sites were chosen based on geographic location, variance in parasite prevalence (three sites with relatively high prevalence of parasites, two with low/none) and high hermit crab density. Following the 2021 Western North American heat dome event [37], we also revisited some of the sites we had visited just prior to or during this weather event in order to examine whether an extreme heatwave affected parasite prevalence in the intertidal zone.

### DNA barcoding methods

A representative subset of parasite specimens was collected, photographed, and preserved from each location (S3 Table). In addition, tissue samples from *P. hirsutiusculus* were collected to confirm host species identity. All tissue samples were stored in >95% ethanol at −20°C. DNA extraction was performed using the DNeasy Blood & Tissue Kit (QIAGEN) following the manufacturer’s protocol with the exception that we used a final elution volume of 50 µL Buffer AL. DNA Barcoding was used to target a fragment of the mitochondrial cytochrome c oxidase subunit I (COI) gene using primers jgLCO1490: 5’ -TIT CIA CIA AYC AYA ARG AYA TTG G- 3’, and jgHCO2198: 5’ -TAI ACY TCI GGR TGI CCR AAR AAY CA- 3’ [46]. Each PCR reaction contained 0.6 µL of each primer, 12.5 µL of FroggaBio 2xtaq mastermix, 3.75 µL of BSA, 2 µL of DNA template, and 5.55 µL of nuclease free water. Thermocycling conditions used a polymerase activation of 94°C for 3 min followed by 38 cycles of denaturation and amplification at 94°C for 30 sec, 47°C for 45 sec and 72°C for 1 min, followed by final elongation step of 72°C for 8 min. PCR amplification product was visualized on a 1.5% agarose gel stained with Red Safe (FroggaBio). PCR product was submitted to Génome Québec Centre d’Expertise et de Services (Montréal, Canada) for Sanger sequencing. Some additional sequences targeting the same gene region were obtained by sending tissue samples to the Canadian Centre for DNA Barcoding. Sequences were obtained in both forward and reverse directions and edited using Geneious V.11.0.4 software. A consensus sequence was constructed by merging the forward and reverse sequences via de novo assembly. Taxonomic assignment was carried out using BLAST searches (blastn) of the NCBI nucleotide database (nr/nt) and the Barcode of Life Data Systems (BOLD). To better refine the taxonomic relationships of the *Peltogaster* spp., we constructed a neighbor-joining tree using our *Peltogaster* sequences along with representative sequences from each species that has publicly accessible COI sequence data. A publicly accessed sequence of *Balanus crenatus* Brugière, 1789 [47,48] (GenBank: KT208786.1) was used as the outgroup; this species was chosen as an outgroup because it represents a distantly related non-parasitic barnacle species. The accession numbers of publicly accessed sequences are included in S3 Fig. Host specimens were accessioned to the Beaty biodiversity museum (S3 Table). The parasite specimens used for DNA barcoding did not have enough remaining tissue after DNA extraction to be archived as museum specimens; photos and DNA sequences for these specimens were deposited in BOLD (dx.doi.org/10.5883/DS-GEHMAN). To provide a global context, a literature review of *Peltogaster paguri* worldwide distribution was conducted (supplemental methods).

### Thermal metabolic experiment methods

#### Animal collection and husbandry.

Experiments were performed with *Pagurus hirsutiusculus* that were uninfected or infected by the rhizocephalan *Peltogaster* sp. Infection was determined by the presence of a visible externa. Due to logistical constraints, we were not able to dissect hermit crabs to detect internal infections. We also collected ovigerous crabs, with this status determined by the presence of a visible egg clutch on the abdomen. We collected all hermit crabs used for the metabolic experiment at the beach on the end of Henson Rd. in Bowser, BC (49.424381, −124.647435) on June 26, 2021. Before the experiment, approximately 55 hermit crabs were held individually in glass jars (237 ml/8 oz) to prevent cannibalism and exposure to potential rhizocephalan larvae. As jars were limited, additional hermit crabs were kept in group housing in tanks (separated by infection and ovigerous status) and then transferred to jars when a jar became available due to a crab death (approximately 20 crabs were eventually replaced). Jars and tanks were kept in a flood table at a temperature of 12.2°C and filled with seawater that was sourced from the Vancouver Aquarium. Water changes and feeding were conducted once a week. Hermit crabs were starved for 4–8 days prior to the metabolic experiment. Crabs were kept in the lab for an acclimation period of approximately one month prior to beginning experiments to ensure consistent acclimation, as differing acclimations can affect thermal tolerance [49]. During this time, most of the ovigerous crabs released their eggs, and were then considered formerly ovigerous. Moulting and survival data was also taken from a previous collection and is included in the supplement.

#### Experimental methods.

To determine whether rhizocephalan infection alters host thermal performance, we examined the effects of temperature on oxygen consumption of crabs that were infected (externa present) and visually uninfected. Because we could not rule out the possibility that crabs lacking externally visible signs of infection were harboring early-stage internal infections, we also included a category for crabs that were ovigerous when collected, as this ensured that they were not infected by a castrating parasite. We measured oxygen consumption at five temperatures, 12°C, 17°C, 22°C, 27°C, and 32°C, maintained via a seawater bath. The temperature of the bath was held at 12°C for at least 15 minutes before beginning measurements and then was gradually increased from a starting temperature of 12°C to 32°C at a rate of approximately 4.3 ± 0.5 °C per hour. Crabs were held individually in open mason jars in the water bath while waiting to be measured, and individually in sealed 20 mL oxygen sensor vials during measurements (OXVIAL20, PyroScience). Four crabs were measured simultaneously, selecting at least one with each infection status, during the temperature ramping phase. Respirometry was performed using fiber optic sensors affixed to the glass of each vial. Sensors were calibrated using a two-point calibration (100% air saturation and 0% air saturation). We used a FireSting O_2_ meter to record oxygen consumption at each temperature setpoint for 10 minutes. Exact temperatures were recorded by the instruments and oxygen saturations were automatically adjusted by the instrument’s accompanying software. There was some deviation from set temperature, but measurements were typically within one degree of the target, except for one round reaching 1.5°C above the set temperature (32°C), which we removed from analysis. Upon completion of the last measurement, we removed the crabs from their shells and dried them with a paper towel to determine wet weight and confirmed their infection status. We removed crabs that had isopod parasites or hyperparasites from analysis. We completed a total of 15 ramps and measured 16 formerly ovigerous, 12 non ovigerous, and 24 infected individuals. We returned the crabs to their jars in the flood table after the temperature ramp. We then checked for survival after 1–5 days, and then again during the weekly water changes and at the completion of the experiments, 38 days after the first ramp day.

This study was carried out on non-cephalopod invertebrates, so was exempt from submitting a protocol to the UBC Animal Use Committee. We collected animals under a Fisheries and Oceans Canada permit (XR 196 2020). Field studies were conducted at the foreshore, which the public is licensed to use in British Columbia. When necessary, permits were obtained to gain access to the foreshore by transiting through Metro Vancouver parks after hours (PAC_Abbott_2021).

### Quantification and statistical analysis

Statistical analyses for the field distribution and thermal metabolic experiments were conducted in R (version 4.4.2) [50] using RStudio (2024.12.0 + 467) [51] and all model settings were left as defaults unless stated otherwise. We used Tidyverse (version 2.0.0.) [52] to assist with data management. We set alpha = 0.05 as our threshold for significance.

#### Field distribution analysis.

We used a generalized linear mixed effect model to determine probability of parasitization for an individual in relation to the fixed effects, salinity, date, dactyl, and host relative abundance for both *Peltogaster* sp. and *Eremitione giardi* during the surveys. In the case of sites being visited multiple times, we only included the data from the first visit in this analysis. If a salinity sample was missing, the site visit was removed from analysis. We calculated host relative abundance as the number of *P. hirsutiusculus* found on average per five-minute search. Site was set as a random effect to account for differences in individuals coming from different populations. The occurrence of the parasite within an individual was set as a binomial response variable. Date was set as a random effect to account for the effect of time across sites. Models were created using the glmmTMB function in the “glmmTMB” R package (version 1.1.9) [53]. Assumptions were examined using the VarCorr function in “lme4” (version 1.1.35.3) and the *plotresid* function in “RVAidememoire” (version 0.9.83.7) [54,55]. Probability of infection was not analyzed for *Peltogasterella* sp. due to limited observations of the parasite. Mixed model visualizations were created using “visreg” (version 2.7.0) [56]. Prevalence graphs were produced using “ggplot2” (version 3.5.1) and “RColorBrewer” (version 1.1.3) [57,58]. Maps were created using QGIS (version 3.34.4) [59]. Shapefiles were obtained from Statistics Canada and the United States Census Bureau [60–62].

#### Thermal metabolic experiment analysis.

During data processing, we removed the first 30 seconds to account for stabilization of the sensor after sealing the chamber. We screened and removed outlying values (i.e., positive slopes, clearly visually abnormal readings likely due to sensor issues or air bubbles). This resulted in some crabs having thermal performance curves fits with fewer than 5 measurements across the temperature range. We calculated the slope of change in oxygen concentration in each chamber by using a linear regression of oxygen over time; slopes were adjusted to account for microbial respiration by subtracting the slope values from chambers without a crab. The resulting slopes were normalized to chamber volume and crab biomass (wet weight) to give crab respiration rates. We used a linear mixed effects regression to determine the effect of temperature and group on metabolic rate. Infection status and temperature were fixed effects, with both temperature and temperature squared included to account for non-linearity of the model. Individual was included as a random effect on the intercept of the model. We created the model using the “lme4” package in R (version 1.1.35.3) [54]. Assumptions were examined using the *visreg* function in the “visreg” package (version 2.7.0), and the *plotresid* function in the “RVAideMemoire” package (version 0.9.83.7) [55,56]. We used a type III Anova in the “lmertest” package (version 3.1.3) and the Tukey method using the *glht* function in the “multcomp” package to compare the curves between the groups (version 1.4.25) [63,64].

Post thermal experiment crab survivorship was analyzed with a Cox proportional hazards analysis using the “survival” package in R (version 3.5.8) [65]. To determine whether mortality differed in formerly ovigerous, non-ovigerous, and infected groups. Graphs were created using the “survminer” R package (version 0.4.9) [66]. We tested the proportional hazards assumption using the *cox.zph* function in the “survival” package [65], and visually checked for nonlinearity and outliers with the *ggcoxdiagnostics* function in the “survminer” package [66].

## Results

### Field distribution

**Fig 1:**
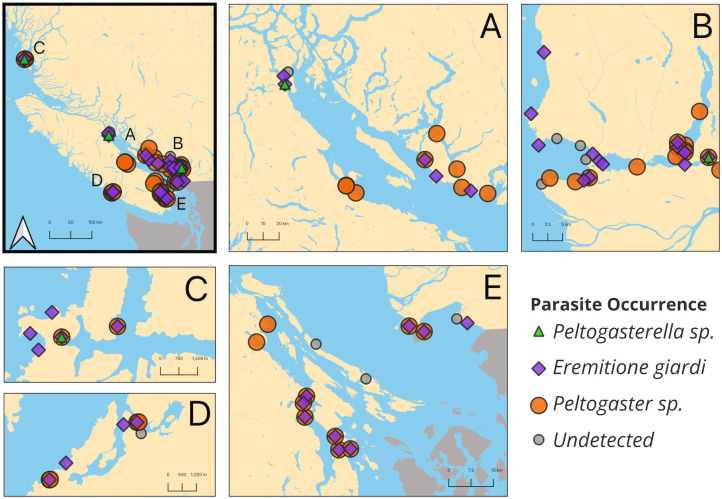
Map of all sites visited in BC, with orange dots indicating the presence of *Peltogaster* sp., purple diamonds indicating the presence of *E. giardi*, green triangles indicating the presence of *Peltogasterella* sp., and gray dots indicating no parasites detected. Panel A depicts the survey sites on east central Vancouver Island and the sunshine coast. Panel B depicts the sites in Burrard inlet and Howe Sound. Panel C depicts the sites on Calvert Island. Panel D depicts the sites in the Barkley Sound. Panel E depicts the sites on south Vancouver Island, the gulf islands, and boundary bay. Maps were created using shapefiles obtained from Stats Canada and the US Census Bureau [60–62].

A total of 4,221 crabs were surveyed for prevalence in this study, with 5–426 crabs examined (includes multiple site visits, mean number examined per site visit was 45.3871, with a standard deviation of 28.76669) per site at 65 sites. At least one parasite species was detected at most of the sites surveyed and parasites were found in all regions that were sampled (S1 Table and Fig 1). Both *Peltogaster* sp. and *E. giardi* were widespread. *E. giardi* was found at 37 sites, with prevalence ranging from 0% to 33.3%. *Peltogaster* sp. was found at 37 of the sites with prevalence ranging from 0% to 44.4%. *Peltogaster* sp. was rarely found with its hyperparasite *Liriopsis pygmaea* (Rathke, 1843) [67,68], with only 11 occurrences throughout the surveys at five sites, where its prevalence on *Peltogaster* sp. ranged from 7.6% to 50% (i.e., one of two *Peltogaster* sp. found) (S2 Table). *Peltogasterella* sp. was found at just three sites, with only one individual present at each.

**Fig 2:**
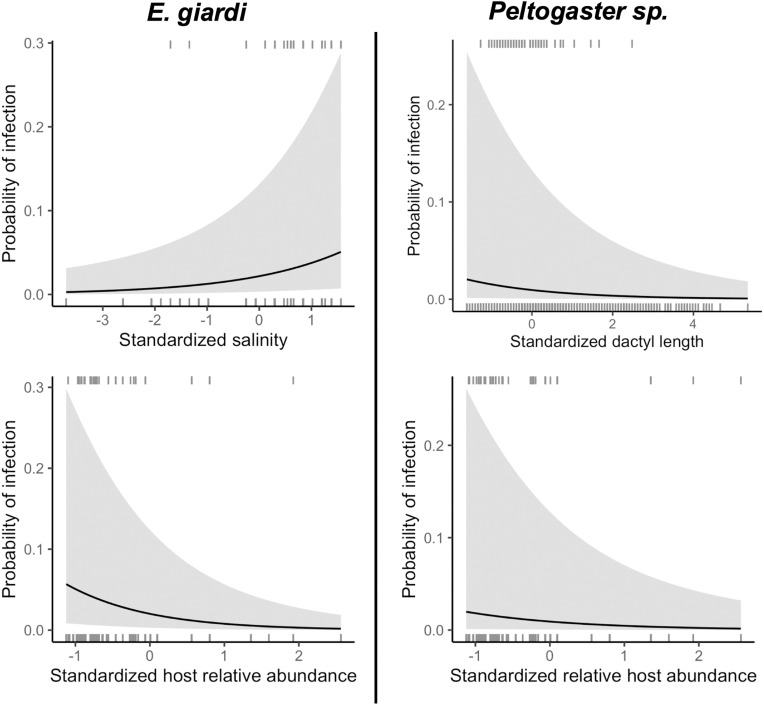
The probability of *P. hirsutiusculus* being parasitized by *E. giardi.* or *Peltogaster* sp. across each fixed effect that was found to be significant during the field surveys (salinity, host size (dactyl length), host relative abundance). Model predictions are shown as the black lines, and the spread of observations are depicted as tick marks along the bottom and tops of each graph, representing the presence or absence of parasites. Fixed effects are shown as the scaled values.

The prevalence of *Peltogaster* sp. was significantly negatively correlated to both host size and host relative abundance, but was not correlated to salinity (Table 1, Fig 2). In the case of *E. giardi*, prevalence was significantly positively related to salinity, negatively related to host relative abundance, and unrelated to host size (Table 1, Fig 2).

**Table 1:** The probability of *P. hirsutiusculus* being parasitized by *E. giardi.* or *Peltogaster* sp. during the BC surveys expressed as odds ratios in relation to salinity, dactyl length, date, and host relative abundance. Values were generated from a generalized linear mixed effect model with a binomial response variable. The odds ratio represents the strength and direction of association of the fixed effect and the probability of the host being parasitized, with an odds ratio of 1 being no association. 95% confidence intervals for the odds ratios are shown. σ^2^ represents the variance of the random effects. Marginal R^2^ represents the variation attributed to the fixed effects, and conditional R^2^ represents the variation attributed to both the fixed and random effects.

	*E. giardi*			*Peltogaster *sp*.*		
*Predictors*	*Odds Ratios*	*CI*	*p*	*Odds Ratios*	*CI*	*p*
(Intercept)	0.02	0.01–0.04	<0.001	0.01	0.01–0.03	<0.001
salinity	1.75	1.16–2.64	**0.008**	1.45	0.81–2.58	0.209
dactyl	1.13	0.90–1.43	0.290	0.62	0.46–0.83	**0.001**
host relative abundance	0.38	0.24–0.62	**<0.001**	0.50	0.29–0.88	**0.016**
**Random Effects**						
σ2	3.29			3.29		
τ00_site_	0.06			0.32		
τ00_date_	0.86			2.32		
ICC	0.22			0.44		
N_site_	51			51		
N_date_	39			39		
Observations	2125			2126		
Marginal R2/ Conditional R2	0.232/ 0.401			0.124/ 0.513		

**Fig 3:**
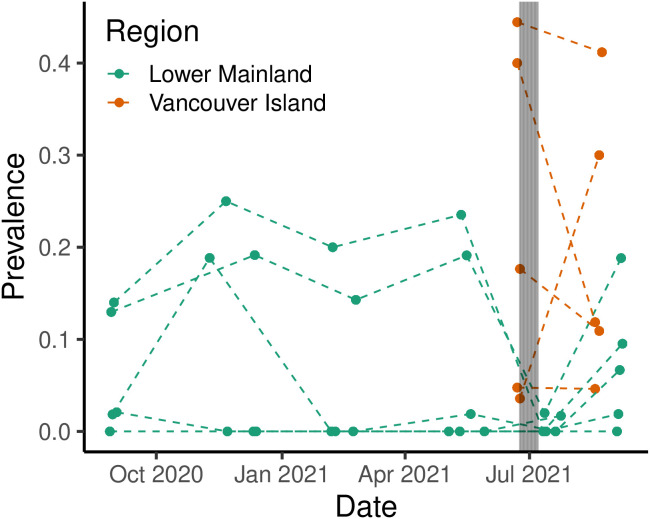
The prevalence of *Peltogaster* sp. through time. The June 2021 heatwave is indicated by a shaded area. Each dot represents a survey, and each survey from each respective site is connected by dashed lines.

We observed a decline in prevalence of *Peltogaster* sp. following the 2021 western North American heat dome event at most but not all of the sites (Fig 3). Specifically, we found a decline in prevalence at 8 of the sites, an increase at two of the sites and no change at two of the sites that started with 0 prevalence. The two initially high prevalence sites in the lower mainland sites dropped in prevalence to less than or equal 0.2% after the heat wave. By early September, we saw prevalence recover at these two sites. The Vancouver Island sites we visited approximately a month after the heat wave had more variable changes, with two sites that declined, one that slightly increased, and one site that saw an approximately 8-fold increase.

### DNA barcoding

We obtained sequences from six hermit crab specimens collected from Calvert Island. These were all > 99% similar to public reference sequences of *Pagurus hirsutiusculus*, which confirms the morphological identification of the host species (BIN: BOLD:AAF9920). We found three genera of externally visible parasites on *P. hirsutiusculus* as well as a hyperparasite during our field surveys in British Columbia.

We sequenced four specimens of an isopod parasite collected from Calvert Island that was morphologically identified as *Eremitione giardi* (Family Bopyridae). They appear to represent a single species (>98% sequence similarity among samples; BIN: BOLD:ABA9934), but did not match any publicly available reference sequences. There are no previous public reference sequences available for *E. giardi* so we are unable to confirm this identification genetically.

We sequenced two specimens of a hyperparasitic isopod found on *Peltogaster* sp. that was morphologically identified as *Liriopsis pygmaea* (Family Cryptoniscidae). These sequences are 98.5% similar to each other (BIN: BOLD:AEE2051). There were no close genetic matches between the sequences obtained for these specimens and public databases, and there are also no previous public reference sequences available for *L. pygmaea,* so we are unable to provide a genetic confirmation of the morphological identification.

From our collection of parasitic barnacles (S3 Table) we obtained sequence data from 13 *Peltogaster* spp. specimens collected from Calvert Island, a single specimen morphologically similar to *Peltogasterella* sp. from Quadra Island, and 9 *Peltogaster* spp. specimens found on a different host species, *Pagurus granosimanus* (Stimpson, 1859) [69,70], collected from Maple Bay. These data provide evidence for three distinct species (S3 Fig). Specimens collected from *P. hirsutiusculus* on Calvert Island (n = 13) form the first genetic group (>99% similar to each other; BIN: BOLD:AEE6401), and are referred to as *Peltogaster* sp.1 in S1 and S2 Tables. These sequences are only ~90% similar to the closest publicly available reference sequence (*P. paguri*; GenBank: KT208574.1), suggesting that they either belong to a species that lacks previous reference sequences, or they may be an undescribed species. Alternatively, it is notable that the *P. paguri* reference sequence used for this analysis is from the North Atlantic Ocean, so we cannot discount the possibility that the genetic distance between this group and *P. paguri* is attributed to intraspecific divergence across a large geographic distance. The second genetic group is composed of the specimens found infecting *Pagurus granosimanus*, collected from Maple Bay (n = 9). These specimens are 99% similar to each other (BIN: BOLD:ADU9408) and also 99% similar to a reference sequence of *Peltogaster boschmai* Reinhard, 1944 [36,71] collected in the San Juan Island (GenBank: MN138416.1; S4 Table). The third genetic group, referred to as *Peltogasterella* sp. consists of the single *Peltogasterella*-like specimen (BIN: BOLD:AEG6825) collected from Quadra Island. This specimen is only ~70% similar to the Calvert Island clade of *Peltogaster* sp. and is 90% similar to the nearest public reference sequence (*Peltogasterella gracilis*; GenBank: LC013686.1; S4 Table).

### Thermal metabolic experiment

**Fig 4:**
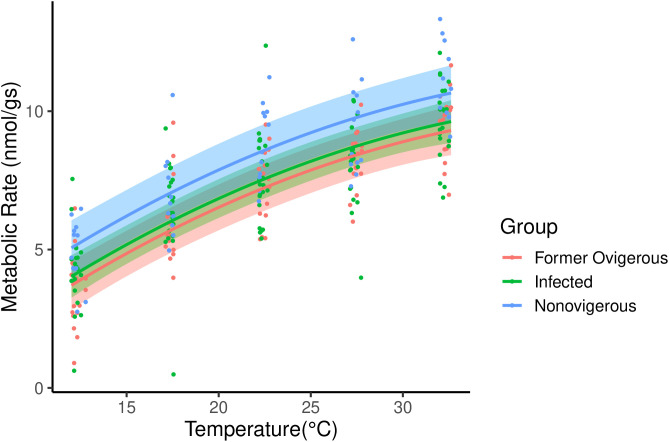
The metabolic rates (nmol O_2_ g^-1^s^-1^) for hermit crab individuals that were formerly ovigerous (red, n  = 16), infected with *Peltogaster* sp. (green, n = 23), or non-ovigerous (blue, n = 12). Each individual was measured at five temperatures from 12-32°C. The dots represent individual metabolic rate observations, and the lines depict the linear mixed effects model for each group.

We found a significant positive, curvilinear relationship between temperature and metabolic rate, and no differences in metabolic rate between the groups when compared via Tukey analysis (Fig 4, Tables 2–3)

**Table 2:** Type III analysis of variance table for the mixed linear models for each group across the temperature range.

	Sum of Squares	Mean of Squares	DF	F value	Pr(>F)
temperature	67.306	67.306	1	27.8084	3.501e-07 ***
I(temperature)	17.849	17.849	1	7.3745	0.007202 **
group	12.269	6.134	2	2.5345	0.08992

**Table 3:** Tukey analysis summary table comparing the linear models for each experimental group.

	Estimate	Std. Error	z value	Pr(>|z|)
infected – formerly ovigerous	0.3207	0.5297	0.605	0.8165
nonovigerous – formerly ovigerous	1.3534	0.6212	2.169	0.0744
nonovigerous – infected	1.0327	0.5791	1.783	0.1743

**Fig 5:**
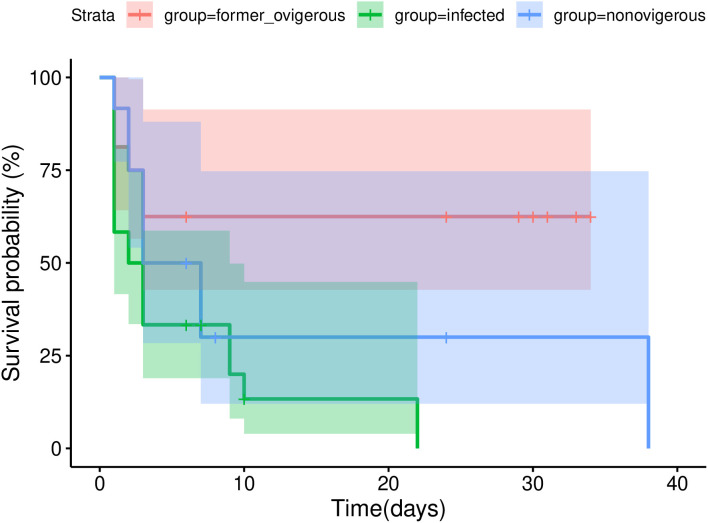
The probability of survival (determined by proportional hazards analysis) of *P. hirsutiusculus* post-temperature ramping metabolic experiment. Shaded areas represent the 95% confidence intervals. Censor points are depicted as dashes through the two solid lines.

There was a significant difference in the probability of survival between the *Peltogaster* sp*.* infected *P. hirsutiusculus* (n = 24, p = 0.005, hazard ratio = 1.48–9.6) compared to formerly ovigerous individuals (n = 16, reference, hazard ratio = 1), but not the non-ovigerous individuals (n = 12, p = 0.149, hazard ratio = 0.75–6.4) following the temperature ramping experiment, when compared using a Cox proportional hazard analysis (Fig 5). In all groups, the probability of survival decreased over time, with infected crabs having the lowest probability of survival after 10 days, and formerly ovigerous crabs having the highest probability of survival.

## Discussion

### Summary

Anthropogenic climate change is projected to have numerous environmental effects such as warming temperatures and salinity changes [72]. Understanding how species interactions will be altered as a result of these changes is essential for determining the threats of climate change to species and their communities [73,74]. As parasites are so ubiquitous and influential, it is important to consider them in ecological studies, especially in the context of environmental change [8,17]. Our research showcases the widespread presence of parasites in hermit crabs. Multiple species of externally visible parasites were found throughout British Columbia during our surveys. The two most common parasites of the hermit crab *Pagurus hirsutiusculus* in British Columbia are both relatively abundant and widespread but differ in both distribution patterns and the factors associated with these patterns. Although we did not find a significant effect of infection on metabolic rate, we did observe increased mortality in infected crabs following the heat stress metabolic experiment, which may partially explain the decrease in field prevalence seen at many sites after the BC heat wave. These results highlight the complexity and importance of studying the interplay between host-parasite interactions and environmental conditions.

### DNA barcoding

We found reports of *Peltogaster paguri* in Europe, Asia, North America, and South America (S1 Methods and S2 Fig) as well as records in 28 different host species (S5 Table). Taken at face value, these findings suggest that *Peltogaster* sp. has low host specificity in pagurid hermit crabs and a global distribution. However, our DNA barcoding on presumed *P. paguri* located in BC, Canada (S3 Fig) and other reports from Oregon, USA (R. Emlet personal communication) suggest that different species could be infecting *P. hirsutiusculus* in this region.

The high number of different recorded host species is also unexpected for rhizocephalans as they have internal stages and root systems within their hosts [14]. Therefore, they must be able to evade the host’s immune system and also avoid killing their host, which has been shown to limit the number of hosts they are able to successfully infect [75,76]. Given the extent of coevolution that is likely required to produce successful infections [75,76], it seems unlikely that “*P. paguri*” as described in the literature represents a single species. Molecular analysis of rhizocephalans in Korea found most species occurred on only one or two hosts and found evidence of multiple undescribed species of rhizocephalans [77]. These results suggest that additional molecular analyses should be completed on presumed *Peltogaster paguri* throughout their range to improve understanding of distributions within the genus *Peltogaster*, as well as to determine whether *P. paguri* represents a cryptic species complex. Further molecular investigations would improve our understanding of rhizocephalan diversity and help to resolve whether different parasitic species may have differing ecologies and effects on their host.

### *Peltogaster* sp. distribution and prevalence

*Peltogaster* sp. was found at about 53% of the sites that were surveyed in BC (Fig 1). Studies on *Peltogaster reticulata* Shiino, 1943 [78,79] found decreased larval survival at low salinities [80]. However, salinity was not a significant factor associated with *Peltogaster* sp. prevalence which may indicate that this parasite is relatively well adapted to low salinities. This suggests this parasite could be resilient to future salinity change in the Salish Sea (predicted to be toward saltier conditions [81]).

Host size (dactyl length) affected the probability of infection by *Peltogaster* sp. during the surveys. Host size is positively related to parasite infection in other rhizocephalan systems [11,42], but interestingly, we found the reverse relationship, in that the probability of infection was higher in smaller size classes. These results are especially interesting as a review of existing literature found that Rhizocephala in the family Peltogastridae (of which *Peltogaster* sp. is a member) tend to occur more frequently in larger hosts [82]. The lower infection rate in larger bodied hosts could indicate that the parasite has a significant impact on host mortality, decreasing the likelihood that they will survive long enough to reach maximum body size. We found that infection did not influence hermit crab mortality when housed in the lab under non stressful conditions (S4 Fig), but that following heat stress infected crabs did indeed have increased mortality.

*Peltogaster* sp. infection may also decrease growth rates, which could prevent their hosts from reaching large sizes. There are several species of rhizocephalan known to suppress host moulting, so it is conceivable that this species uses a similar mechanism [14]. However, lab observations found that moulting was similar between infected and uninfected individuals (S5 Fig and S1 Results). We only evaluated one moult cycle over a relatively short period, so further work evaluating long term growth rates is needed. O’Brien and van Wyk [82] suggested that smaller sizes of infected hosts might be the result of host feminization in species that are sexually dimorphic. Male *P. hirsutiusculus* do tend to be larger than females in the northern part of their range [83]. Sexual dimorphism may also help to explain this trend if females are more susceptible to infection than males, which has been seen in some *Peltogaster* species [84]. Future research in this system should sex *P. hirsutiusculus* during infection checks to examine this.

In contrast to our predictions, infection was more likely to be found at lower host densities. Because members of peltogastrid Rhizocephala are typically castrators [14], a high infection rate may serve to lower host abundance by serving as population control. Parasitic castrators acting as population regulators has been documented in snail-trematode host-parasite systems [2], and increasing prevalence of parasitic castrators, including the rhizocephalan *Sacculina carcini* Thompson, 1836 [85,86] has also been associated with decreasing host biomass [87]. However, given the lack of direct evidence of population regulation by rhizocephalans, and the likely high hermit crab larval dispersal distance due to the larval stage duration, this seems unlikely [88]. Another explanation is that this parasite is more likely to saturate host populations when the host is rare, if parasite infections are driven by parasite larval supply and host populations are limited by something else (e.g., shell availability [89]). Further research in this area, such as more detailed population modeling, is needed to better understand the relationship between prevalence and hermit crab host populations.

### *Eremitione giardi* distribution and prevalence

*Eremitione giardi* also has a widespread distribution and, like *Peltogaster* sp., *E. giardi* was found at 53% of the visited sites (Fig 1). The factors related to prevalence also differed between the two parasites. As we predicted, salinity had a significant effect on *E. giardi* prevalence, with a higher probability of the host being parasitized at higher salinities (Fig 2). This is similar to what has been found in other parasite systems [12]. *Eremitione giardi* itself might not be well adapted to low salinities [90], or it could be an effect this parasite has on its host. *Eremitione giardi* pierces the host to feed on hemolymph in the gill chamber, which is the primary site of ion exchange [91,92]. This lifestyle may cause a detrimental effect on the host’s ability to cope with low salinities [93] causing parasitized hosts to be unable to persist within low salinity environments. Salinity in the Strait of Georgia is highly influenced by outflow from the Fraser River, particularly during the freshet [94], and peak discharge rates are predicted to be lower in the future due to decreases in the available snowpack to supply the summer melt [95]. Historic trends also indicate increasing sea surface salinities in the Strait of Georgia [81], which could make this area more hospitable for this parasite. Experiments on the relationship between *E. giardi* prevalence and salinity are warranted to untangle whether this is a parasite-based response or an effect of parasitism on the host’s’ salinity tolerance. It is also possible that variability in other life stages could drive the observed salinity patterns (e.g., the intermediate copepod host stage for *E. giardi)*.

Dactyl length, a proxy for host size, was not found to have a significant effect on prevalence of *E. giardi.* Unlike rhizocephalans which can infect hosts that have already reached adulthood, *E. giardi* likely begins parasitizing hosts while they are in juvenile or megalopa stage [96,97]. Therefore, parasitization should not be more frequently found in larger/older hosts simply because of age, as increased age does not increase opportunity to become parasitized. The non-significance of host size (dactyl length) suggests that this parasite may not decrease growth rates or cause premature mortality. As with *Peltogaster* sp., we found a higher probability of the host being parasitized at lower host relative abundance. It is intriguing that both external parasites evaluated for this host show a similar unexpected pattern and suggests a fruitful venue for future research.

### Thermal metabolic experiment

As expected, we found a significant, positive relationship between temperature and metabolic rate in all crab groups at 12°C to 32°C. We did not find infection status (infected by *Peltogaster* sp.*,* formerly ovigerous, or non-ovigerous) to be related to a detectable difference in metabolic rate. The non-ovigerous group did have a non-significant trend towards higher metabolic rates. We measured the combined metabolic rate of host and parasite in this experiment, since separation would lead to mortality of the parasite, and possibly the host. As such, the overall lack of difference in metabolic rate between infected and uninfected/formerly ovigerous individuals could suggest that an infected host has the same metabolic resources available to it to supply both its own functions and the functions of the parasite. Recent research on lipids in shrimp rhizocephalans has shown that hosts may be able to compensate for resource use by the parasite [98]. However, it is also possible that this may come at a cost (see survivorship results). Additionally, other species in the *Peltogaster* genus have been observed to make up a substantial part (17.78%) of the volume of the host [99], so are likely to also make a significant contribution to the mass. If this is the case, we may not be correctly estimating the metabolic rate for infected crabs, as the biomass of the actual crab is lower. There may be a reduced metabolic capacity in infected crabs that could lead to reduced post marine heat wave survival. This possibility is supported both by our post heat stress experimental survival results, and the trends we observed in the field after the 2021 heat dome. However, there are several other possibilities which could have led to our observed field results. Infected crabs could have altered their hiding behaviour/tidal height preference during the heat event.

There may be a detrimental effect of infection on survival post-exposure to heat stress. In a previous experiment, we did not find a difference in survival between infected and uninfected crabs when held at 12.2°C under laboratory conditions (S4 Fig), however, in the post-heat ramp experiment we saw higher survival in formerly ovigerous (uninfected) than in the infected and non-ovigerous groups. There was no difference in survival between the infected and non-ovigerous groups, which could indicate that a proportion of the non-ovigerous crabs had internal early stages of infection. Alternatively, there may be physiological changes that occur in recently ovigerous crabs or sex differences (as other categories were likely a mix of sexes) that could make them more resilient to heat stress. We suggest that research on transcriptomic response should be conducted on healthy, formerly ovigerous, and infected hermit crabs to help elucidate the mechanism of these heat response results.

## Conclusions

Overall, we found parasite occurrences on *P. hirsutiusculus* to be frequent in BC. The two most common parasites of *Pagurus hirsutiusculus* in BC, *Peltogaster* sp*.* and *E. giardi*, had different factors related to prevalence. Exploring the interaction between abiotic and biotic factors for multiple types of parasites is critical to be able to understand and model future changes in populations. Interestingly, the prevalence of both *E. giardi* and *Peltogaster* sp. did not show the expected density dependence, suggesting that there could be some consistent unmeasured factor creating these patterns. Although we did not find a significant effect of infection on metabolic rate, our post heat stress survivorship results suggest that *Peltogaster* sp. infection may lead to decreased survival of infected crabs after thermal stress events. This is also supported by the trends we observed after the 2021 heat dome event. Further research in this area is important for a better understanding of how host parasite dynamics will be affected by future environmental change, and how communities will be shaped as a result.

## Supporting information

S1 Methods More details about the procedures for the lab and field methods. Also includes the methods for the earlier moulting and ambient temperature survival experiment as well as the literature review of *Peltogaster* sp. occurrences.
(DOCX)

S1 Results A summary of results from the moulting and ambient temperature survival experiment. (DOCX)

S1 Fig Parasites found during the BC field surveys. (A) *Peltogaster* sp. along with its hyperparasite *Liriopsis pygmaea* (LP), as indicated by the arrow. (B) the multiple externa of *Peltogasterella* sp. (C) *Eremitione giardi* after removal from *P. hirsutiusculus*. (D) externa of *Peltogaster* sp. (P1) on *P. hirsutiusculus*. (E) *P. hirsutiusculus* infected with *Peltogasterella* sp. (P2). (F) *P. hirsutiusculus* showing the bulging carapace indicative of parasitization by *E. giardi*, indicated by the arrow.
(DOCX)

S2 Fig Map of records of *Peltogaster paguri* obtained from the literature review of historic and modern occurrences of the species. Records are shown as red dots.
(DOCX)

S3 Fig A tree of the genetic relationships between *Peltogaster* specimens and selected publicly available sequences. Group I consists of *Peltogaster* sp. samples infecting *Pagurus hirsutiusculus*, group II consists of likely *Peltogaster boschmai* samples found on *Pagurus granosimanus,* and group III consists of a *Peltogasterella* sp. sample.
(DOCX)

S4 Fig The probability of survival over time for *P. hirsutiusculus* infected with *Peltogaster* sp. and *P. hirsutiusculus* without this infection, when held in the laboratory at 12.2°C. Shaded areas represent the 95% confidence intervals. Censor points are depicted as dashes through the two solid lines.
(DOCX)

S5 Fig Cumulative hazard over time (cumulative hazard calculated from time to first moult rather than time to death) for *P. hirsutiusculus* with *Peltogaster* sp. infection and those without this infection. Shaded areas represent the 95% confidence intervals. Censor points are depicted as dashes through the two solid lines.
(DOCX)

S1 Table The sites visited during this study, with our sample size and prevalence of the *Peltogaster* sp. and *E. giardi* parasites (or occurrence record, if applicable). (DOCX)

S2 Table *Liriopsis pygmaea* occurrences, prevalence and site information. (DOCX)

S3 Table Collection, voucher, and accession information for the specimens that were DNA barcoded. (XLSX)

S4 Table Genetic similarity within and among the three clades of *Peltogaster* sp. identified in this study. The top portion of this table shows all pairwise comparisons expressed as % similarity among the three clades. The bottom portion of this table compares the three clades to public data from several *Peltogaster* species obtained from GenBank.
(DOCX)

S5 Table The occurrences and prevalence of *Peltogaster paguri* found during the literature review. (DOCX)

S1 File Strikingimage. (TIFF)

## References

[1] Carlson CJ, Dallas TA, Alexander LW, Phelan AL, Phillips AJ. What would it take to describe the global diversity of parasites? Proc Biol Sci. 2020;287(1939):20201841. doi: 10.1098/rspb.2020.1841 33203333 PMC7739500

[2] Lafferty K. Effects of parasitic castration on growth reproduction and population dynamics of the marine snail Cerithidea californica. Mar Ecol Prog Ser. 1993;96:229–37.

[3] Lafferty KD, Dobson AP, Kuris AM. Parasites dominate food web links. Proc Natl Acad Sci U S A. 2006;103(30):11211–6. doi: 10.1073/pnas.0604755103 16844774 PMC1544067

[4] Gehman ALM, Hall RJ, Byers JE. Host and parasite thermal ecology jointly determine the effect of climate warming on epidemic dynamics. Proc Natl Acad Sci. 2018;115(4):744–9. 29311324 10.1073/pnas.1705067115 PMC5789902

[5] Hector TE, Sgrò CM, Hall MD. Thermal limits in the face of infectious disease: how important are pathogens?. Glob Chang Biol. 2021;27(19):4469–80. doi: 10.1111/gcb.15761 34170603

[6] Kirk D, Jones N, Peacock S, Phillips J, Molnár PK, Krkošek M, et al. Empirical evidence that metabolic theory describes the temperature dependency of within-host parasite dynamics. PLoS Biol. 2018;16(2):e2004608. doi: 10.1371/journal.pbio.2004608 29415043 PMC5819823

[7] Gehman AM, Satterfield DA, Keogh CL, McKay AF, Budischak SA. To improve ecological understanding, collect infection data. Ecosphere. 2019;10(6). doi: 10.1002/ecs2.2770

[8] Wood CL, Johnson PT. A world without parasites: exploring the hidden ecology of infection. Front Ecol Environ. 2015;13(8):425–34. doi: 10.1890/140368 28077932 PMC5222570

[9] Arneberg P, Skorping A, Grenfell B, Read AF. Host densities as determinants of abundance in parasite communities. Proc R Soc B Biol Sci. 1998;265(1403):1283–9.

[10] Blower SM, Roughgarden J. Parasites detect host spatial pattern and density: a field experimental analysis. Oecologia. 1989;78(1):138–41. doi: 10.1007/BF00377209 28311913

[11] Sloan LM, Anderson SV, Pernet B. Kilometer-scale spatial variation in prevalence of the rhizocephalan Lernaeodiscus porcellanae on the porcelain crab Petrolisthes cabrilloi. J Crustac Biol. 2010;30(2):159–66.

[12] Tolley SG, Winstead JT, Haynes L, Volety AK. Influence of salinity on prevalence of the parasite Loxothylacus panopaei in the xanthid Panopeus obesus in SW Florida. Dis Aquat Organ. 2006;70(3):243–50. doi: 10.3354/dao070243 16903236

[13] Alvarez F, Hines AH, Reaka-Kudla ML. The effects of parasitism by the barnacle Loxothylacus panopaei (Gissler) (Cirripedia: Rhizocephala) on growth and survival of the host crab Rhithropanopeus harrisii (Gould) (Brachyura: Xanthidae). J Exp Mar Biol Ecol. 1995;192(2):221–32.

[14] Høeg JT. The biology and life cycle of the Rhizocephala (Cirripedia). J Mar Biol Assoc U K. 1995;75(3):517–50.

[15] Gissler C. The crab parasite, Sacculina. Am Nat. 1884;18:225–9.

[16] WoRMS Editorial Board. *Loxothylacus panopaei* (Gissler, 1884) [Internet]. World Register of Marine Species. 2025 [cited 2025 July 31]. Available from: https://www.marinespecies.org/aphia.php?p=taxdetails&id=395096

[17] Byers JE. Marine parasites and disease in the era of global climate change. Ann Rev Mar Sci. 2021;13:397–420. doi: 10.1146/annurev-marine-031920-100429 32520636

[18] Dana J. Conspectus crustaceorum quae in orbis terrarum circumnavi gatione, Carolo Wilkes e classe Reipublicae Foederatae Duce, lexit et descripsit. Paguridea. Proc Acad Nat Sci Phila. 1851;5:267–72.

[19] WoRMS Editorial Board. *Pagurus hirsutiusculus* (Dana, 1851) [Internet]. World Register of Marine Species. 2025 [cited 2025 July 31]. Available from: https://www.marinespecies.org/aphia.php?p=taxdetails&id=366699

[20] McDermott JJ, Williams JD, Boyko CB. The unwanted guests of hermits: a global review of the diversity and natural history of hermit crab parasites. J Exp Mar Biol Ecol. 2010;394(1–2):2–44.

[21] Hart JFL. Crabs and their relatives of British Columbia. British Columbia: British Columbia Provincial Museum; 1984. p. 267.

[22] Rathke H. Beiträge zur vergleichenden Anatomie und Physiologie, Reisebemerkungen aus Skandinavien, nebst einem Anhange über die rückschreitende Metamorphose der Thiere. Nueste Schriften Naturforschenden Ges Danzing. 1842;3(4):1–162.

[23] WoRMS Editorial Board. *Peltogaster paguri* Rathke, 1842 [Internet]. World Register of Marine Species. 2025 [cited 2025 July 31]. Available from: https://www.marinespecies.org/aphia.php?p=taxdetails&id=134798

[24] Boschma H. On the larval forms of Rhizocephala. Proc Sect Sci K Akad Van Wet Te Amst. 1927;30:293–7.

[25] WoRMS Editorial Board. *Peltogasterella gracilis* (Boschma, 1927) [Internet]. World Register of Marine Species. 2025 [cited 2025 July 31]. Available from: https://www.marinespecies.org/aphia.php?p=taxdetails&id=463717

[26] Calman WT. ON a collection of crustacea from Puget Sound. Ann N Y Acad Sci. 1898;11(1):259–92. doi: 10.1111/j.1749-6632.1898.tb54972.x

[27] WoRMS Editorial Board. *Eremitione giardi* (Calman, 1898) [Internet]. World Register of Marine Species. 2025 [cited 2025 July 31]. Available from: https://www.marinespecies.org/aphia.php?p=taxdetails&id=1329201#sources

[28] Warrenchuk JJ, Shirley TC. Parasitism by the rhizocephalan *Peltogaster paguri* Rathke, 1842 and hyperparasitism by the bopyrid isopod *Liriopsis pygmaea* (Rathke, 1843) on *Pagurus hirsutiusculus* (Dana, 1851) in Southeastern Alaska. Crustaceana. 2000;73(8):971–7. doi: 10.1163/156854000505029

[29] Markham JC. Extension of range and new host records for the parasitic isopod Pseudione giardi Caiman in the northeastern Pacific. Wasmann J Biol. 1974;32(2):195–201.

[30] George RY, Stromberg JO. Some new species and new records of marine isopods from San Juan archipelago, Washington, U.S.A. Crustaceana. 1968;14(3):225–54.

[31] Hebert PDN, Penton EH, Burns JM, Janzen DH, Hallwachs W. Ten species in one: DNA barcoding reveals cryptic species in the neotropical skipper butterfly Astraptes fulgerator. Proc Natl Acad Sci U S A. 2004;101(41):14812–7. doi: 10.1073/pnas.0406166101 15465915 PMC522015

[32] Johnson SB, Warén A, Vrijenhoek RC. DNA barcoding of *Lepetodrilus* limpets reveals cryptic species. J Shellfish Res. 2008;27(1):43–51. doi: 10.2983/0730-8000(2008)27[43:dbollr]2.0.co;2

[33] Hoeg JT, Lutzen J. Life cycle and reproduction in the Cirripedia, Rhizocephala. Oceanogr Mar Biol Annu Rev. 1995;33:427–85.

[34] Williams JD, Boyko CB. The global diversity of parasitic isopods associated with crustacean hosts (Isopoda: Bopyroidea and Cryptoniscoidea). PLoS One. 2012;7(4):e35350. doi: 10.1371/journal.pone.0035350 22558143 PMC3338838

[35] Boschma H. Papers from Dr. Th. Mortensen’s Pacific expedition 1914–16. LV. Rhizocephala. Vidensk Meddelelser Fra Dan Naturhistoriske Foren. 1931;89:297–380.

[36] Reinhard EG. Rhizocephalan parasites of hermit crabs from the Northwest Pacific. J Wash Acad Sci. 1944;34(2):49–58.

[37] White RH, Anderson S, Booth JF, Braich G, Draeger C, Fei C, et al. The unprecedented Pacific Northwest heatwave of June 2021. Nat Commun. 2023;14(1):727. doi: 10.1038/s41467-023-36245-5 36759624 PMC9910268

[38] Raymond WW, Barber JS, Dethier MN, Hayford HA, Harley CDG, King TL, et al. Assessment of the impacts of an unprecedented heatwave on intertidal shellfish of the Salish Sea. Ecology. 2022;103(10):e3798. doi: 10.1002/ecy.3798 35726191 PMC9786359

[39] Brownlee G. Thermal Tolerance, Herbivory and Tide Pool Distributions of *Littorina sitkana* and *Littorina scutulata*: Implications for a Warming World [Bsc(hons). Thesis]. Vancouver, BC, CA: University of British Columbia; 2018.

[40] Taylor PR. Environmental resistance and the ecology of coexisting hermit crabs: thermal tolerance. J Exp Mar Biol Ecol. 1982;51:229–36.

[41] Baker KD, Fifield DA, Mullowney DRJ, Skanes KR. Ecology and epidemiology of the striped shrimp, Pandalus montagui Leach, 1814 (Decapoda: Caridea), in the northern Labrador Sea, Davis Strait, and Ungava Bay, Canada. J Crustac Biol. 2021;41(2):ruab024.

[42] Gehman A-LM, Grabowski JH, Hughes AR, Kimbro DL, Piehler MF, Byers JE. Predators, environment and host characteristics influence the probability of infection by an invasive castrating parasite. Oecologia. 2017;183(1):139–49. doi: 10.1007/s00442-016-3744-9 27722800

[43] Smith AE, Chapman JW, Dumbauld BR. Population structure and energetics of the bopyrid isopod parasite *Orthione griffenis* in mud shrimp Upogebia pugettensis. J Crustac Biol. 2008;28(2):228–33.

[44] Campos J, Ribas F, Bio A, Freitas V, Souza AT, van der Veer HW. Sacculina carcini impact on energy content of the shore crab *Carcinus maenas* L. Parasitology. 2023;149(12):1536–45. 10.1017/S0031182022000993 PMC11010527 35924593

[45] Abrams P. Shell selection and utilization in a terrestrial hermit crab, Coenobita compressus. J Exp Marine Biol Ecol. 1978;253:239–53. 10.1007/BF00345169 28309552

[46] Geller J, Meyer C, Parker M, Hawk H. Redesign of PCR primers for mitochondrial cytochrome c oxidase subunit I for marine invertebrates and application in all-taxa biotic surveys. Mol Ecol Resour. 2013;13(5):851–61. doi: 10.1111/1755-0998.12138 23848937

[47] Bruguière J. Encyclopédie méthodique ou par ordre de matières. 1789.

[48] WoRMS Editorial Board. *Balanus crenatus* Bruguière, 1789 [Internet]. World Register of Marine Species. 2025 [cited 2025 July 31]. Available from: https://www.marinespecies.org/aphia.php?p=taxdetails&id=106215

[49] Cuculescu M, Hyde D, Bowler K. Thermal tolerance of two species of marine crab, *Cancer pagurus* and *Carcinus maenas*. J Therm Biol. 1998;23(2):107–10.

[50] R Foundation for Statistical Computing. R: A language and environment for statistical computing. [Internet]. Vienna, Austria; 2024. Available from: https://www.R-project.org/

[51] Posit Team. RStudio: integrated development environment for R. Boston, MA: Posit Software, PBC; 2024.

[52] Wickham H, Averick M, Bryan J, Chang W, McGowan L, François R, et al. Welcome to the Tidyverse. JOSS. 2019;4(43):1686. doi: 10.21105/joss.01686

[53] Brooks ME, Kristensen K, Benthem KJ, van, Magnusson A, Berg CW, Nielsen A, et al. glmmTMB Balances Speed and Flexibility Among Packages for Zero-inflated Generalized Linear Mixed Modeling. R J. 2017;9(2):378. doi: 10.32614/rj-2017-066

[54] Bates D, Mächler M, Bolker B, Walker S. Fitting linear mixed-effects models using lme4. J Stat Softw. 2015;67(1).

[55] Maxime HERVE. RVAideMemoire: Testing and Plotting Procedures for Biostatistics. 2023; Available from: https://CRAN.R-project.org/package=RVAideMemoire

[56] Breheny P, Burchett W. Visualization of Regression Models Using visreg. R J. 2017;9(2):56–71.

[57] Wickham H. ggplot2: Elegant Graphics for Data Analysis. Springer-Verl N Y [Internet]. 2016; Available from: https://ggplot2.tidyverse.org

[58] Neuwirth E. RColorBrewer: ColorBrewer Palettes {R package version 1.1-3}. 2022; Available from: https://CRAN.R-project.org/package=RColorBrewer

[59] QGIS.org. QGIS Geographic Information System [Internet]. Open Source Geospatial Foundation Project; 2023. Available from: http://qgis.org

[60] Statistics Canada. 2016 Census-Boundary Files- Provinces [Internet]. 2016. Available from: https://www12.statcan.gc.ca/census-recensement/2011/geo/bound-limit/bound-limit-2016-eng.cfm

[61] Statistics Canada. 2016 Census-Boundary Files- Lakes and Rivers [Internet]. 2016. Available from: https://www12.statcan.gc.ca/census-recensement/2011/geo/bound-limit/bound-limit-2016-eng.cfm

[62] US Census Bureau. Cartographic Boundary Files – Shapefile. (division 500k) [Internet]. 2018. Available from: https://www.census.gov/geographies/mapping-files/time-series/geo/carto-boundary-file.html

[63] Kuznetsova A, Brockhoff PB, Christensen RHB. lmerTest Package: Tests in Linear Mixed Effects Models. J Stat Softw. 2017;82(13):1–26.

[64] Hothorn T, Bretz F, Westfall P. Simultaneous inference in general parametric models. Biom J. 2008;50(3):346–63. 18481363 10.1002/bimj.200810425

[65] Therneau TM. A Package for Survival Analysis in R. 2024 [cited 2024 July 26]; Available from: https://cran.r-project.org/web/packages/survival/citation.html

[66] Kassambara A, Kosinski M, Biecek P. survminer: Drawing Survival Curves using “ggplot2.” 2021; Available from: {https://CRAN.R-project.org/package=survminer}

[67] Rathke H. Beiträge zur Fauna Norwegens. Nova Acta Acad Caesareae Leopoldino-Carol Naturae Curiosorum. 1843;20:1–264.

[68] WoRMS Editorial Board. *Liriopsis pygmaea* (Rathke, 1843) [Internet]. World Register of Marine Species. 2025 [cited 2025 July 31]. Available from: https://www.marinespecies.org/aphia.php?p=taxdetails&id=147002#distributions

[69] Stimpson W. Notes on North American Crustacea, no. 1. Ann Lyceum Nat Hist N Y. 1859;7(11):49–93.

[70] WoRMS Editorial Board. *Pagurus granosimanus* (Stimpson, 1859) [Internet]. World Register of Marine Species. 2025 [cited 2025 July 31]. Available from: https://www.marinespecies.org/aphia.php?p=taxdetails&id=366694

[71] WoRMS Editorial Board. *Peltogaster boschmai* Reinhard, 1944 [Internet]. World Register of Marine Species. 2025 [cited 2025 July 31]. Available from: https://www.marinespecies.org/aphia.php?p=taxdetails&id=463708

[72] Pörtner HO, Roberts DC, Masson-Delmotte V, Zhai P, Tignor M, Poloczanska E, Weyer NM. The Ocean and Cryosphere in a Changing Climate: Special Report of the Intergovernmental Panel on Climate Change [Internet]. Cambridge University Press; 2019 [cited 2024 June 16]. Report No.: 1155. Available from: https://www.cambridge.org/core/product/identifier/9781009157964/type/book

[73] Kordas RL, Dudgeon S, Storey S, Harley CDG. Intertidal community responses to field‐based experimental warming. Oikos. 2014;124(7):888–98. doi: 10.1111/oik.00806

[74] Kordas RL, Harley CDG, O’Connor MI. Community ecology in a warming world: The influence of temperature on interspecific interactions in marine systems. J Exp Mar Biol Ecol. 2011;400(1–2):218–26.

[75] Kuris AM, Goddard JHR, Torchin ME, Murphy N, Gurney R, Lafferty KD. An experimental evaluation of host specificity: the role of encounter and compatibility filters for a rhizocephalan parasite of crabs. Int J Parasitol. 2007;37(5):539–45. doi: 10.1016/j.ijpara.2006.12.003 17275825

[76] Goddard JHR, Torchin ME, Kuris AM, Lafferty KD. Host specificity of Sacculina carcini, a potential biological control agent of the introduced European green crab Carcinus maenas in California. Biol Invasions. 2005;7(6):895–912. doi: 10.1007/s10530-003-2981-0

[77] Jung J. The host range and distribution pattern of rhizocephalan parasitic barnacles in Korean coasts and their relationship with geographical factors. Front Mar Sci. 2024;11.

[78] Shiino S. Rhizocephala of Japan. J Sigenkagaku Kenkyusyo. 1943;1:1–36.

[79] WoRMS Editorial Board. *Peltogaster reticulata* Shiino, 1943 [Internet]. World Register of Marine Species. 2025 [cited 2025 July 31]. Available from: https://www.marinespecies.org/aphia.php?p=taxdetails&id=842789

[80] Kashenko SD, Korn OM. Combined effects of seawater temperature and salinity on development of the larvae of the rhizocephalan Peltogaster reticulatus. Russ J Mar Biol. 2003;29(3).

[81] Iwabuchi BL, Gosselin LA. Long-term trends and regional variability in extreme temperature and salinity conditions experienced by coastal marine organisms on Vancouver Island, Canada. Bull Mar Sci. 2019;95(3):337–54.

[82] O’Brien J, Wyk PV. Effects of Crustacean Parasitic Castrators (Epicaridean Isopods and Rhizocephalan Barnacles) on Growth of Crustacean Hosts. In: Crustacean Issues 3. Routledge; 1985.

[83] Blackstone NW. Size, shell-living and carcinization in geographic populations of a hermit crab, Pagurus hirsutiusculus. J Zool Lond. 1989;217:477–90.

[84] Reinhard EG. Studies on the life history and host-parasite relationship of Peltogaster paguri. Biol Bull. 1942;83(3):401–15.

[85] Thompson J. Natural history and metamorphosis of an anomalous crustaceous parasite of Carcinus maenas, the Sacculina carcini. Entomol Mag. 1836;3:452–6.

[86] WoRMS Editorial Board. *Sacculina carcini* Thompson, 1836 [Internet]. World Register of Marine Species. 2025 [cited 2025 July 31]. Available from: https://www.marinespecies.org/aphia.php?p=taxdetails&id=134805

[87] Torchin ME, Lafferty KD, Kuris AM. Release from parasites as natural enemies: Increased performance of a globally introduced marine crab. Biological Invasions. 2001;3(4):333–45. doi: 10.1023/a:1015855019360

[88] Fitch BM, Lindgren EW. Larval development of *Pagurus hirsutiusculus* (Dana) reared in the laboratory. Biol Bull. 1979;156(1):76–92.

[89] Spight TM. Availability and use of shells by intertidal hermit crabs. Biol Bull. 1977;152(1):120–33.

[90] Studer A, Poulin R. Effects of salinity on an intertidal host-parasite system: is the parasite more sensitive than its host?. J Exp Mar Biol Ecol. 2012;412:110–6.

[91] Péqueux A. Osmotic Regulation in Crustaceans. J Crustac Biol. 1995;15(1):1–60.

[92] Bursey C. Histopathology of the parasitization of *Munida iris* (Decapoda: Galatheidae) by *Munidion irritans* (Isopoda: Bopyridae). Bull Mar Sci. 1978;28(3):566–70.

[93] Moles A, Pella JJ. Effects of parasitism and temperature on salinity tolerance of the kelp shrimp *Eualus suckleyi*. Trans Am Fish Soc. 1984;113:354–9.

[94] Cummins PF, Masson D. Climatic variability and trends in the surface waters of coastal British Columbia. Prog Oceanogr. 2014;120:279–90.

[95] Johannessen S, Macdonald R. Effects of local and global change on an inland sea: the Strait of Georgia, British Columbia, Canada. Clim Res. 2009;40:1–21.

[96] Pike RB. Observations on epicaridea obtained from hermit-crabs in British waters, with notes on the longevity of the host-species. Ann Mag Nat Hist. 1961;4(40):225–40.

[97] Strathmann MF. Reproduction and development of marine invertebrates of the Northern Pacific Coast: data and methods for the study of eggs, embryos, and larvae. University of Washington Press; 1987.

[98] Yoshioka RM, Brown S, Treneman NC, Schram JB, Galloway AWE. A Rhizocephalan Parasite Induces Pervasive Effects on Its Shrimp Host. Biol Bull. 2023;244(3):201–16. 38457679 10.1086/729497

[99] Nagler C, Hörnig MK, Haug JT, Noever C, Høeg JT, Glenner H. The bigger, the better? Volume measurements of parasites and hosts: Parasitic barnacles (Cirripedia, Rhizocephala) and their decapod hosts. PLoS One. 2017;12(7):e0179958. doi: 10.1371/journal.pone.0179958 28678878 PMC5497970

