# Supplementary material for: Parasites of the hermit crab Pagurus hirsutiusculus; distribution, prevalence, and thermal ecology
Source: PLoS One. 2025 Nov 19;20(11):e0335145. doi: 10.1371/journal.pone.0335145 (PMC12629492; doi:10.1371/journal.pone.0335145)
Supplement: S1 Methods — Also includes the methods for the earlier moulting and ambient temperature survival experiment as well as the literature review of Peltogaster sp. occurrences. (DOCX) [file pone.0335145.s001.docx]

**Supplementary Methods**

**Additional Animal collection and husbandry methods (Temperature Experiment)**

We were not able to age the crabs as they were collected from the field, but we measured their size and weight. An attempt was made to collect similar size ranges in both infected and uninfected categories, estimated by eye. Jars and tubing were rinsed between crabs, when jars opened up due to a crab death. A bubbling airstone was placed in each jar to ensure sufficient oxygenation. If the water level dropped due to over bubbling, they were topped off with fresh seawater. Hermit crabs were fed approximately 3-5 frozen brine shrimp per week. Mortality was recorded at least once a week.

**Laboratory Methods (Temperature Experiment)**

We placed four mason jars and four 20mL oxygen sensor vials in a large plastic container filled with seawater (originating from the Vancouver Aquarium) covered in styrofoam and foil insulation tape. Oxygen sensor vials were stabilized using terracotta plates with holes in the center. We placed aquarium pumps into the bath to ensure water movement and sufficient oxygenation. While not being measured, the crabs were placed individually into the mason jars and while being measured, the hermit crabs were placed in seawater-filled 20mL oxygen sensor vials (OXVIAL20). All vials were outfitted with a magnetic stirbar and stirring speeds were checked prior to experimentation using food colouring to ensure even mixing occurred within 15 seconds. Some metals are known to consume oxygen, thus we used plastic coated magnetic stirbars.

The order and placement of the crabs was randomized. We started by including one crab from each group and then randomly assigning the 4th vial. After Aug 10, we began using two infected and one from each of the formerly ovigerous and nonovigerous groups to ensure we had an adequate number of infected crabs. We placed the crabs on mesh shelves in the vials to prevent them from being injured by the stir bar (10mm). We kept the crabs with their shells as Shumway [[1]](https://www.zotero.org/google-docs/?quYuCP) found no difference in O_2_ consumption with and without shells at steady state conditions, and other hermit crab O_2_ consumption studies also allowed them to keep their shells [[2]](https://www.zotero.org/google-docs/?iXuBJZ). We weighed them without their shells after drying them with a paper towel following the treatment. To determine the exact volume of water in each vial, the empty vial, mesh shelf, and crab with shell were also weighed. After the treatment, the full vials with contents were weighed, with the difference in the two measurements representing the mass of the water. The volume of the contents of the vial without a crab was also determined to calculate the background respiration of the water. If the exact mass of the contents of the vial was not known (e.g.if the stir bars and mesh from the vials were mixed up), we used the average of the mass of the four stir bars and mesh to determine the volume.

The vials were connected to fiber optic cables through adapter rings, and attached to the O_2_ meter (FireStingO_2_, PyroScience). Sensors were calibrated using a 100% air saturation water sample created by bubbling an airstone in the water for 10 minutes, and a 0% air saturation water sample created by bubbling N_2_ until measurements reached stability. Sensors were recalibrated when time between experimental runs was greater than a week. The O_2_ meter was attached to a laptop computer with the “Pyroscience oxygen logger” software installed. The large plastic container was placed on top of four stir plates, and covered with a styrofoam and aluminum foil lid to reduce heat transfer and prevent the crabs from reacting to the sight of lab activity. The large container was filled with seawater. We placed coiled up irrigation tubing connected to a water bath (AC 200, with Haake A25) into the bath. We recirculated deionized water through the tubing and water bath to control the temperature in the large seawater container. Thermocouples (Type T) were placed in the seawater container to monitor the temperature. Thermocouple data was processed using the TC-08 thermocouple data logger and Pico logger 6 software. There was some variation in ramping rate due to the fact that ramping was done manually and room temperature varied. Temperature was measured continuously with a probe inserted into the seawater bath and connected to the instrument, allowing for automatic temperature compensation. Before measuring oxygen, we turned on the stir plate to ensure the water in the vial was well mixed. We removed visible air bubbles (if any) with a pipet prior to measuring. We kept caps in the seawater container for the duration of the experiment to limit the introduction of air bubbles. If the caps were left out accidentally, air bubbles were removed again.

At each temperature, we capped the vial, and measured the oxygen consumption in the water with no crab present for 3-5 minutes. After measuring microbial respiration in the chambers, a crab was introduced to the vial and the vial was resealed. We measured the oxygen consumption of the crab for 10 minutes. After 10 minutes of measurement, we returned crabs to their respective mason jars. This process was repeated at all temperature setpoints.

Upon completion of the last measurement, crabs were removed from their shells and weighed. We also confirmed their infection status (some crabs either developed externa during holding period, or were accidentally mixed), as well as measured their left second dactlyl, noted externa colour, and measured the externa. We then returned the crabs to their jars in the flood table. We checked for survival after 1-5 days, and then again during the weekly water changes. The vials with mesh and the containers were rinsed using 70% ethanol between ramp days.

**Field methods**

For logistical reasons, we conducted surveys using two different methods. For sites visited up until August 28 2020, we completed 3-4 sets of 10-minute searches. Timed searches were used as hermit crab distribution tends to be patchy, and this was the most effective way to obtain enough hermit crabs to examine parasite prevalence while still maintaining a measure of host density. Starting on August 28 2020, we began completing 2-8 sets of 5-minute searches to increase efficiency. Average abundance was determined as the number of crabs found on average in a 5-minute search period for a site. At sites visited before August 22nd 2020, we examined around 8-10 crabs from each search and counted the rest. This method led to potential issues with sampling bias as infected hermit crabs were potentially clustered in the order of examination. After Aug 22, we changed methods and examined all crabs found in each search. At each site, we examined enough of the searches (all searches were either examined completely, or counted) to reach 50 crabs when possible. Because of the bias in early sampling, data collected with the first sampling method was only used to detect parasite occurrence, rather than prevalence. Where possible, we included searches before Aug 22nd where all crabs in a search were examined. Sites were chosen based on geographic location, and available opportunity (such as joining another research sampling effort). Towards the end of the sampling, we placed a greater focus on getting a mix of sites with varying amounts of wave exposure/fetch.

If the left dactyl was missing or severely broken, we measured the right dactyl of the second walking leg, as we found them to be similar in length after measuring both dactyls in one site survey. We returned hermit crabs after the measurements. We selected Acadia beach, Belcarra, Kitsilano point, strathcona lookout, and lamborghini cove to resurvey every two-three months, due to proximity to UBC and the initially high prevalence at strathcona and Belcarra and low prevalence at Acadia, Kitsilano, and lamborghini cove.

**Global literature review**

In order to document the range of *Peltogaster paguri,* the presumed parasite found in this system, we conducted a literature review examining all occurrences of the parasite. We searched for the term “*Peltogaster pagur*i” in the Zoological Record database, Web of Science database, Google Scholar, and the Biodiversity Heritage Library database. We also searched these terms and downloaded datasets from OBIS and GBIF in order to obtain additional records, including museum specimens[[3,4]](https://www.zotero.org/google-docs/?Wgzu8Q). Citations for each datapoint or the DOI for the GBIF download are available in the datafile, submitted as a supplement and available on Zenodo upon publication of this manuscript. We recorded every occurrence of the *P. paguri* that included a location with the observation, regardless of host species. When possible, we included non-english papers if we were able to interpret them using google translate. If no geographic coordinates were provided, we searched the name of the location in google maps and recorded the output coordinates. If no date of collection was provided, the date of publication was used. In order to examine the prevalence of members of the *Peltogaster* genus across space and time, we searched google scholar for *“Peltogaster*” AND “prevalence”. We then recorded all prevalence data for this genus that included a location following the protocol above. The internet searches were conducted in summer of 2020.

**Ambient Survival and Moulting Experiment**

Hermit crabs were collected next to the pier at Belcarra Regional Park, B.C. (49.313080, -122.927862) on Oct 12, 2020. An attempt was made to collect similar size ranges in both infected and uninfected categories, estimated by eye. In this experiment, we did not use formerly ovigerous crabs, and simply considered crabs without externa to be uninfected. However, this does mean that there could be internal infection in this group. Hermit crabs were held individually in glass jars (237ml/8oz) to prevent cannibalism and exposure to potential rhizocephalan larvae. Jars were kept in a flood table and held at a consistent temperature of 12.2°C (54°F). They were not used in a manipulative experiment. Husbandry conditions were similar to the temperature experiment. Mortality was also recorded once a week to examine the effect of infection status on survival. Moulting was also recorded beginning on November 9th, 2020 in order to compare growth between infected and uninfected individuals. Survival and moulting were recorded until March 1st 2021.

**Quantification and Statistical Analysis**

All statistical analyses were conducted in R (version 4.4.0)[[5]](https://www.zotero.org/google-docs/?dTL0AS). using Rstudio (Version 2024.04.1+748)[[6]](https://www.zotero.org/google-docs/?pBO3bK) and all model settings were left as defaults unless stated otherwise. Survivorship of the crabs kept in the laboratory was analyzed using a Cox

proportional Hazards analysis using the “survival” package in R [[7]](https://www.zotero.org/google-docs/?pJwHaT) to

determine whether mortality differed in infected and uninfected groups. This statistical

method allowed us to account for the crabs that were placed into the metabolic

experiment and thus removed from the survival study (censusing). The same Cox

proportional hazard analysis was used to compare moulting incidence between infected

and uninfected crabs. To conduct this analysis, the “event” was set as the first moult, as

only one individual moulted twice. Graphs were created using the “survminer” R package [[8]](https://www.zotero.org/google-docs/?gmYogO).The Map for the literature review was created using QGIS [[9]](https://www.zotero.org/google-docs/?72Yoih) (version 3.34.4). Shapefiles were obtained from the website opendatasoft [[10]](https://www.zotero.org/google-docs/?fLD0VN).

**References**

[1. Shumway SE. Osmotic balance and respiration in the hermit crab, pagurus bernhardus, exposed to fluctuating salinities. J Mar Biol Assoc U K. 1978;58(4):869–76.](https://www.zotero.org/google-docs/?WQMeh4)

[2. Thatje S, Casburn L, Calcagno JA. Behavioural and respiratory response of the shallow-water hermit crab Pagurus cuanensis to hydrostatic pressure and temperature. J Exp Mar Biol Ecol. 2010;390(1):22–30.](https://www.zotero.org/google-docs/?WQMeh4)

[3. GBIF.org. GBIF Home Page. 2020; Available from: https://www.gbif.org](https://www.zotero.org/google-docs/?WQMeh4)

[4. OBIS. Ocean Biodiversity Information System. Intergov Oceanogr Comm UNESCO [Internet]. 2020; Available from: obis.org](https://www.zotero.org/google-docs/?WQMeh4)

[5. R Foundation for Statistical Computing. R: A language and environment for statistical computing. [Internet]. Vienna, Austria; 2024. Available from: https://www.R-project.org/](https://www.zotero.org/google-docs/?WQMeh4)

[6. Posit Team. RStudio: Integrated Development Environment for  R. Boston, MA: Posit Software, PBC; 2024.](https://www.zotero.org/google-docs/?WQMeh4)

[7. Terry M. Therneau. A Package for Survival Analysis in R. 2024 [cited 2024 Jul 26]; Available from: https://cran.r-project.org/web/packages/survival/citation.html](https://www.zotero.org/google-docs/?WQMeh4)

[8. Alboukadel Kassambara and Marcin Kosinski and Przemyslaw Biecek. survminer: Drawing Survival Curves using “ggplot2.” 2021; Available from: {https://CRAN.R-project.org/package=survminer}](https://www.zotero.org/google-docs/?WQMeh4)

[9. QGIS.org. QGIS Geographic Information System [Internet]. Open Source Geospatial Foundation Project; 2024. Available from: http://qgis.org](https://www.zotero.org/google-docs/?WQMeh4)

10. World Food Programme (UN agency). 2019. World Administrative Boundaries - Countries and Territories. Contains public sector information licensed under the Open Government Licence v3.0. Available from: https://public.opendatasoft.com/explore/dataset/world-administrative-boundaries/information/?dataChart=eyJxdWVyaWVzIjpbeyJjb25maWciOnsiZGF0YXNldCI6IndvcmxkLWFkbWluaXN0cmF0aXZlLWJvdW5kYXJpZXMiLCJvcHRpb25zIjp7fX0sImNoYXJ0cyI6W3siYWxpZ25Nb250aCI6dHJ1ZSwidHlwZSI6ImNvbHVtbiIsImZ1bmMiOiJDT1VOVCIsInNjaWVudGlmaWNEaXNwbGF5Ijp0cnVlLCJjb2xvciI6IiNGRjUxNUEifV0sInhBeGlzIjoic3RhdHVzIiwibWF4cG9pbnRzIjo1MCwic29ydCI6IiJ9XSwidGltZXNjYWxlIjoiIiwiZGlzcGxheUxlZ2VuZCI6dHJ1ZSwiYWxpZ25Nb250aCI6dHJ1ZX0%3D&location=2,42.07882,0.00845&basemap=jawg.light
