# Supplementary material for: Parasites of the hermit crab Pagurus hirsutiusculus; distribution, prevalence, and thermal ecology
Source: PLoS One. 2025 Nov 19;20(11):e0335145. doi: 10.1371/journal.pone.0335145 (PMC12629492; doi:10.1371/journal.pone.0335145)
Supplement: S1 Results — (DOCX) [file pone.0335145.s002.docx]

**Supplementary Results**

**Global Literature Review**

Our literature review revealed that the presumed *Peltogaster paguri* has a distribution covering much of the northern hemisphere (S. Fig 1) with a large cluster of records concentrated in northern Europe, near the type locality (Norway, Rathke 1842). There was a single record in the southern hemisphere on the coast of Argentina. *P. paguri* was recorded in 28 different host species. The review of prevalence for the genus *Peltogaster* found a range of prevalence from 0-57%. However, the record of 57% was from a sample size of seven crabs and the next highest prevalence of 33.3% had a sample size of just six crabs. The third highest record with a 31.7% prevalence had a sample size of 131.

**Ambient Survival**

There was no significant difference (p=0.957) in the probability of survival

between the uninfected (N=34, HR: 0.98, 95% CI=0.45-2.1) and infected groups (N=25,

reference, hazard ratio=1) when compared using a Cox proportional hazard analysis

(S. Fig 3), when crabs were held at 12.2°C in the lab. In both groups, the probability of survival decreased over time. After about 100 days in the lab, a relatively sharp increase in mortality was observed in both of the groups.

**Moutling**

There was no significant difference (p=0.290) in moutling incidence between

groups infected (N=22, reference, hazard ratio=1) with *Peltogaster sp.* and those that

were not infected (N=33, hazard ratio=0.64, 95% CI=0.28-1.5) when compared using a

Cox proportional hazard analysis (S. Fig 4). The incidence of moulting was slightly higher in the infected group compared to the uninfected group
