# Supplementary material for: Parasites of the hermit crab Pagurus hirsutiusculus; distribution, prevalence, and thermal ecology
Source: PLoS One. 2025 Nov 19;20(11):e0335145. doi: 10.1371/journal.pone.0335145 (PMC12629492; doi:10.1371/journal.pone.0335145)
Supplement: S1 Fig — (A) Peltogaster sp. along with its hyperparasite Liriopsis pygmaea (LP), as indicated by the arrow. (B) the multiple externa of Peltogasterella sp. (C) Eremitione giardi after removal from P. hirsutiusculus. (D) externa of Peltogaster sp. (P1) on P. hirsutiusculus. (E) P. hirsutiusculus infected with Peltogasterella sp. (P2). (F) P. hirsutiusculus showing the bulging carapace indicative of parasitization by E. giardi, indicated by the arrow. (DOCX) [file pone.0335145.s003.docx]

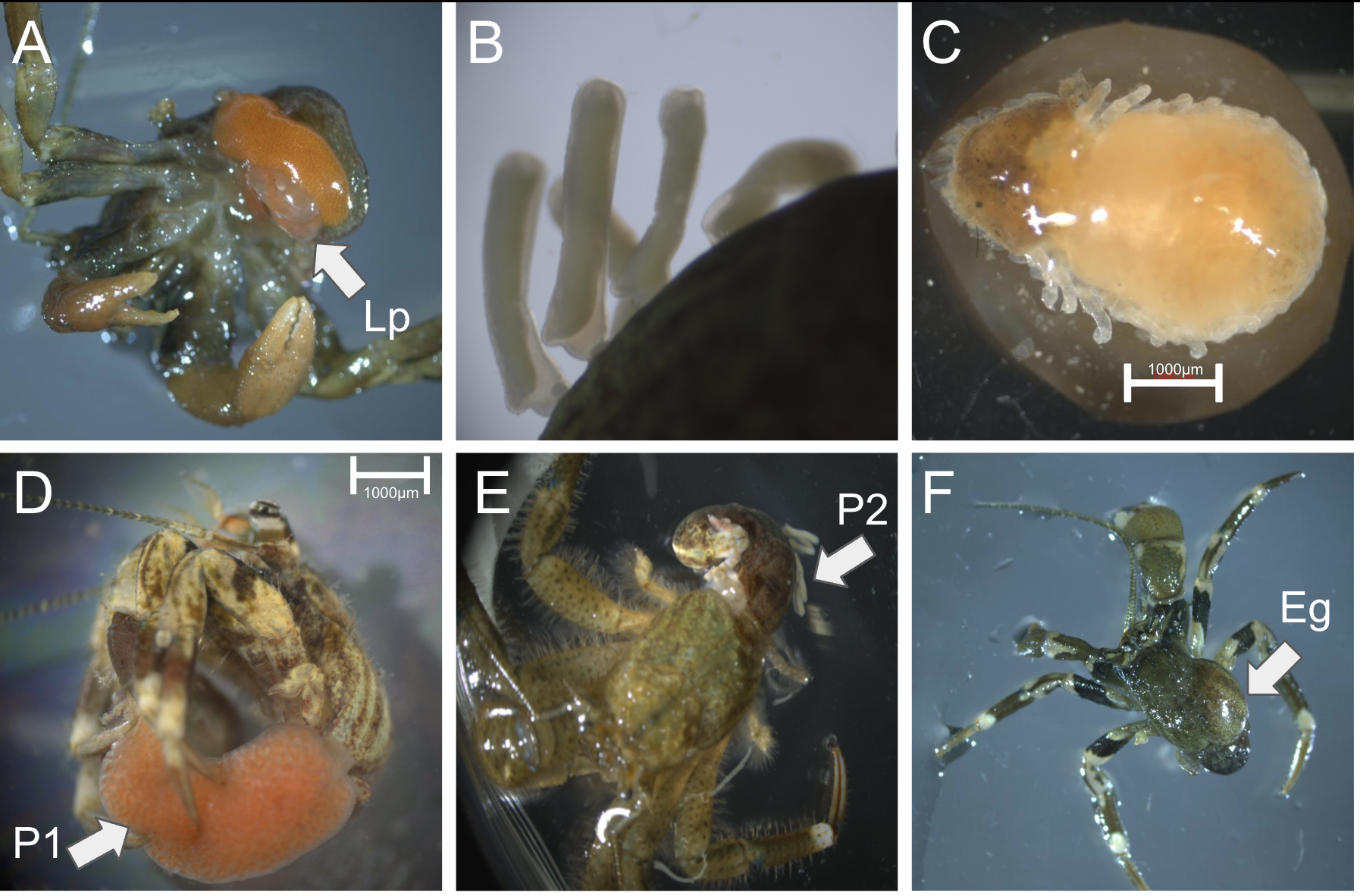


**Figure S1.** Parasites found during the BC field surveys. (A) *Peltogaster* sp. along with its hyperparasite *Liriopsis* *pygmaea*. (LP), as indicated by the arrow. (B) the multiple externa of *Peltogasterella* sp. (C) *Eremitione giardi* after removal from *P. hirsutiusculus*. (D) externa of *Peltogaster* sp. (P1) on *P. hirsutiusculus*. (E) *P. hirsutiusculus* infected with *Peltogasterella* sp. (P2). (F) *P. hirsutiusculus* showing the bulging carapace indicative of parasitization by *E. giardi*, indicated by the arrow with (Eg). Exposure and contrasts were modified in some images to increase clarity.
