# Supplementary material for: Parasites of the hermit crab Pagurus hirsutiusculus; distribution, prevalence, and thermal ecology
Source: PLoS One. 2025 Nov 19;20(11):e0335145. doi: 10.1371/journal.pone.0335145 (PMC12629492; doi:10.1371/journal.pone.0335145)
Supplement: S2 Fig — Records are shown as red dots. (DOCX) [file pone.0335145.s004.docx]

**
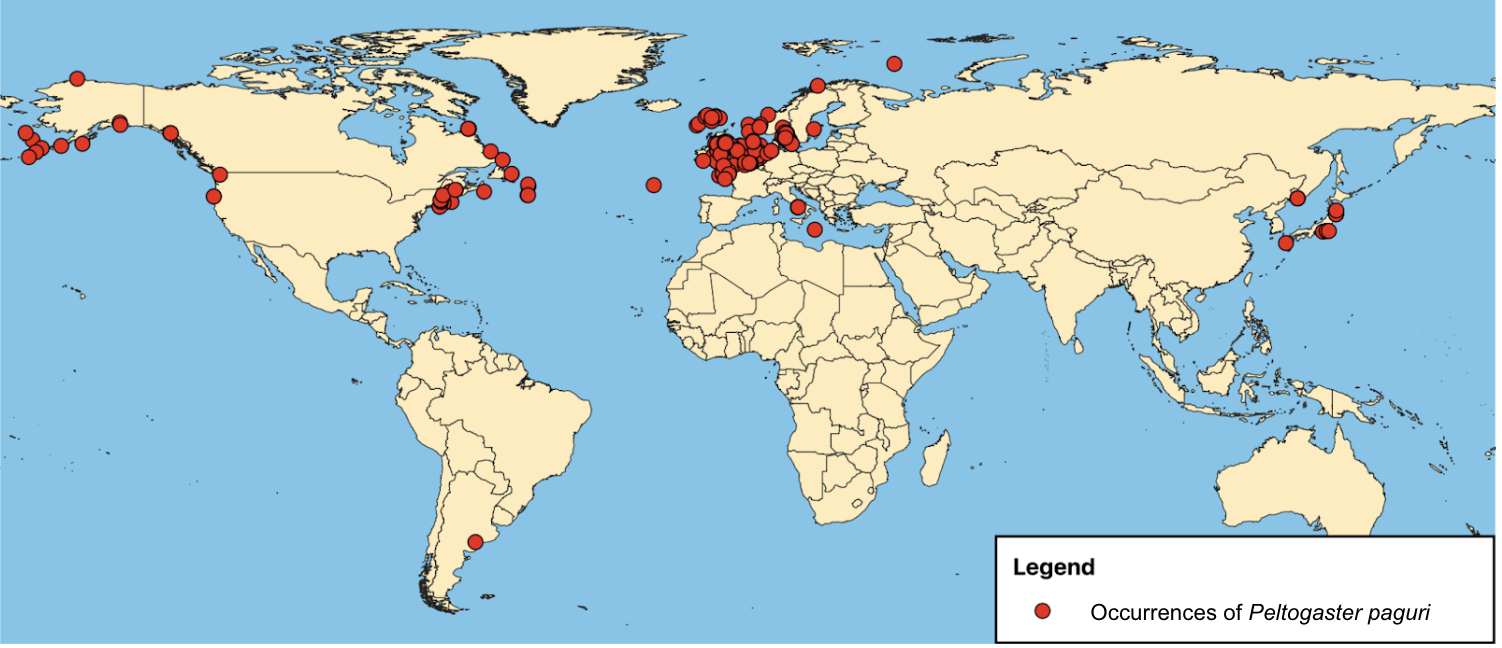
**

**Figure S2.** Map of records of *Peltogaster paguri* obtained from the literature review of historic and modern occurrences of the species. Records are shown as red dots. This map was created in QGIS using shapefiles obtained from the website “opendatasoft” [1].

1. World Food Programme (UN agency). 2019. World Administrative Boundaries - Countries and Territories. Contains public sector information licensed under the Open Government Licence v3.0. Available from: https://public.opendatasoft.com/explore/dataset/world-administrative-boundaries/information/?dataChart=eyJxdWVyaWVzIjpbeyJjb25maWciOnsiZGF0YXNldCI6IndvcmxkLWFkbWluaXN0cmF0aXZlLWJvdW5kYXJpZXMiLCJvcHRpb25zIjp7fX0sImNoYXJ0cyI6W3siYWxpZ25Nb250aCI6dHJ1ZSwidHlwZSI6ImNvbHVtbiIsImZ1bmMiOiJDT1VOVCIsInNjaWVudGlmaWNEaXNwbGF5Ijp0cnVlLCJjb2xvciI6IiNGRjUxNUEifV0sInhBeGlzIjoic3RhdHVzIiwibWF4cG9pbnRzIjo1MCwic29ydCI6IiJ9XSwidGltZXNjYWxlIjoiIiwiZGlzcGxheUxlZ2VuZCI6dHJ1ZSwiYWxpZ25Nb250aCI6dHJ1ZX0%3D&location=2,42.07882,0.00845&basemap=jawg.light
