# Supplementary material for: Parasites of the hermit crab Pagurus hirsutiusculus; distribution, prevalence, and thermal ecology
Source: PLoS One. 2025 Nov 19;20(11):e0335145. doi: 10.1371/journal.pone.0335145 (PMC12629492; doi:10.1371/journal.pone.0335145)
Supplement: S3 Fig — Group I consists of Peltogaster sp. samples infecting Pagurus hirsutiusculus, group II consists of likely Peltogaster boschmai samples found on Pagurus granosimanus, and group III consists of a Peltogasterella sp. sample. (DOCX) [file pone.0335145.s005.docx]

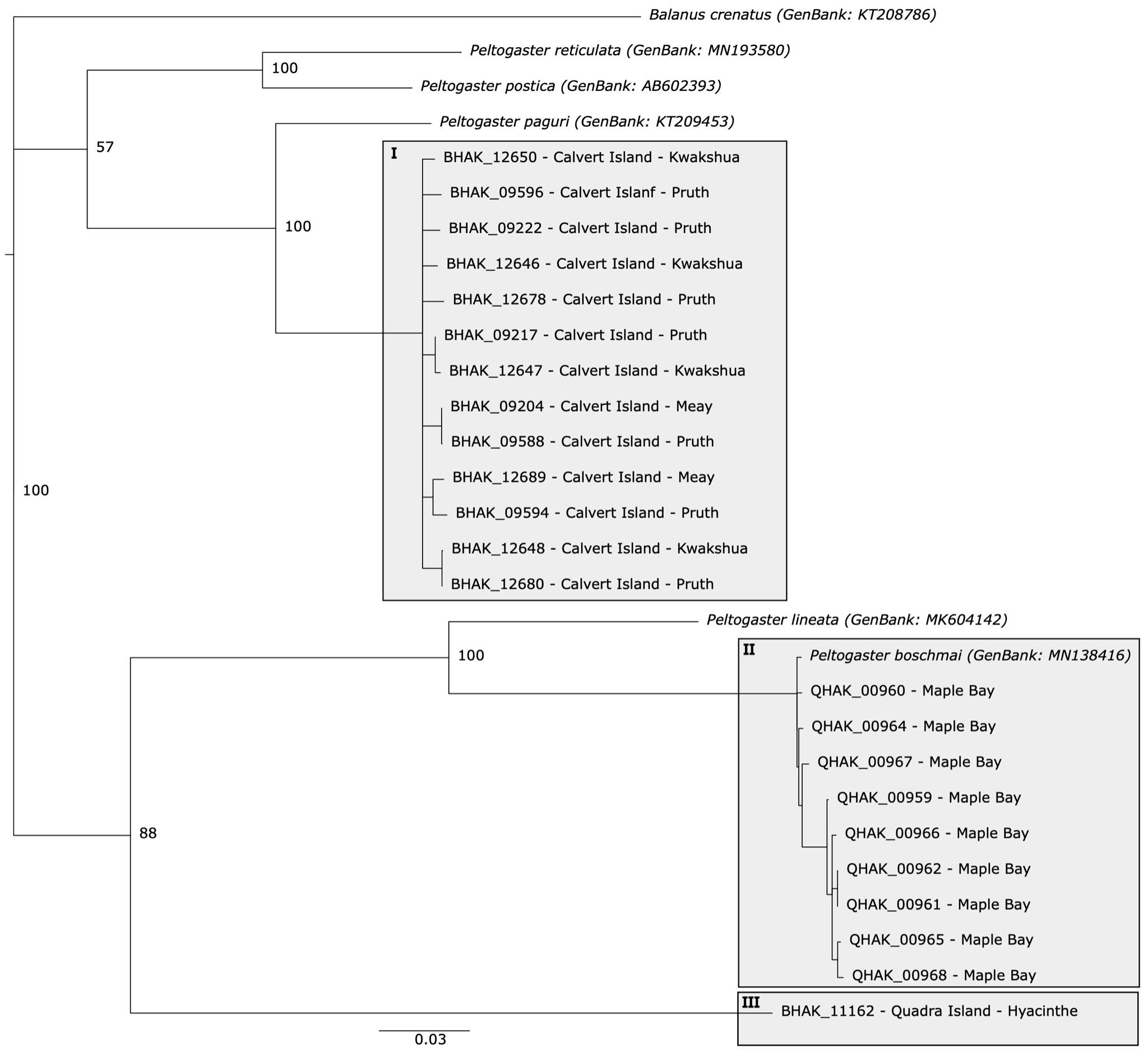


**Figure S3.** A tree of the genetic relationships between *Peltogaster* specimens and selected publically available sequences. Group I consists of *Peltogaster* sp*.* samples infecting *Pagurus hirsutiusculus*, group II consists of likely *Peltogaster boschmai* samples found on *Pagurus granosimanus,* and group III consists of a *Peltogasterella* sp. sample.
