# Supplementary material for: Parasites of the hermit crab Pagurus hirsutiusculus; distribution, prevalence, and thermal ecology
Source: PLoS One. 2025 Nov 19;20(11):e0335145. doi: 10.1371/journal.pone.0335145 (PMC12629492; doi:10.1371/journal.pone.0335145)
Supplement: S4 Fig — Shaded areas represent the 95% confidence intervals. Censor points are depicted as dashes through the two solid lines. (DOCX) [file pone.0335145.s006.docx]

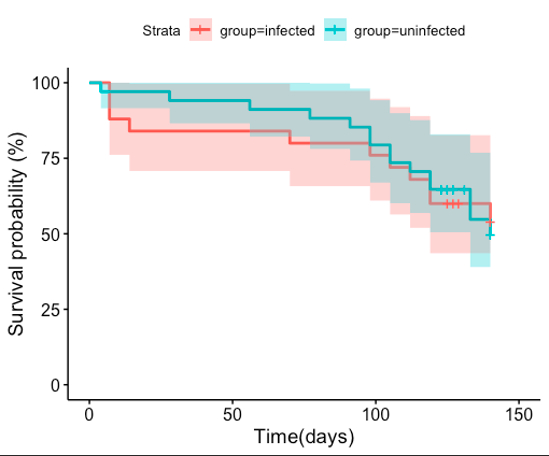


**Figure S4.** The probability of survival over time for *P. hirsutiusculus* infected with *Peltogaster* sp. *and P. hirsutiusculus* without this infection, when held at 12.2^o^C at consistent laboratory conditions. Shaded areas represent the 95% confidence intervals. Censor points are depicted as dashes through the two solid lines.
