# Supplementary material for: Parasites of the hermit crab Pagurus hirsutiusculus; distribution, prevalence, and thermal ecology
Source: PLoS One. 2025 Nov 19;20(11):e0335145. doi: 10.1371/journal.pone.0335145 (PMC12629492; doi:10.1371/journal.pone.0335145)
Supplement: S5 Fig — Shaded areas represent the 95% confidence intervals. Censor points are depicted as dashes through the two solid lines. (DOCX) [file pone.0335145.s007.docx]

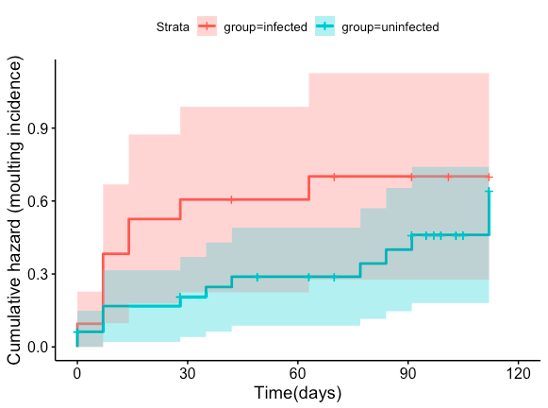


**Figure S5** Cumulative hazard over time (cumulative hazard calculated from time to first moult rather than time to death) for *P. hirsutiusculus* with *Peltogaster* sp. infection and those without this infection. Shaded areas represent the 95% confidence intervals.

Censor points are depicted as dashes through the two solid lines.
