# Supplementary material for: Parasites of the hermit crab Pagurus hirsutiusculus; distribution, prevalence, and thermal ecology
Source: PLoS One. 2025 Nov 19;20(11):e0335145. doi: 10.1371/journal.pone.0335145 (PMC12629492; doi:10.1371/journal.pone.0335145)
Supplement: S1 Table — (DOCX) [file pone.0335145.s008.docx]

**Table S1** The sites visited during this study, with our sample size and prevalence of the *Peltogaster* sp. and *E. giardi* parasites (or occurrence record, if applicable)

| Site | N | | *Peltogaster* sp. | | *E. giardi* | Latitude | | Longitude | |
| --- | --- | --- | --- | --- | --- | --- | --- | --- | --- |
| 9 o'Clock Gun | | 25 | | 0% | 8% | 49.297757 | -123.11849 | |  |
| Acadia | | 274* | | 1% | 0% | 49.279927 | -123.243031 | |  |
| Armours Beach | | NA | | occurrence | NA | 49.403391 | -123.502724 | |  |
| Baker Beach | | 65 | | 7.69% | 7.69% | 49.604606 | -124.040125 | |  |
| Bamfield Boat Launch | | 16 | | 0% | 0% | 48.828832 | -125.127738 | |  |
| Barnet Marine | | NA | | NA | occurrence | 49.29261 | -122.929343 | |  |
| Bedwell Bay | | 80 | | 0% | 0% | 49.31404 | -122.9202 | |  |
| Belcarra | | 360* | | 15.00%** | 0.83% | 49.310169 | -122.927898 | |  |
| Belcarra Pier | | 55 | | 20% | 0% | 49.313051 | -122.927898 | |  |
| Bird's Eye | | 68* | | 19.12% | 1.47% | 48.801389 | -123.601696 | |  |
| Blue Heron Park | | 60* | | 41.67% | 0% | 49.044303 | -123.753372 | |  |
| Bluestone Point | | 12 | | 0 | 8.33% | 48.819545 | -125.16436 | |  |
| Boundary Bay | | 124* | | 0.81% | 6.45% | 49.004715 | -123.035276 | |  |
| Brunswick Beach | | NA | | NA | occurrence | 49.47049 | -123.24428 | |  |
| Cabin Beach | | 47 | | 0% | 0% | 48.978943 | -123.534409 | |  |
| Campbell River | | 12 | | 0 | 8.33% | 50.046361 | -125.251838 | |  |
| Copper Cove | | 30 | | 0 | 10% | 49.378525 | -123.279621 | |  |
| Crescent Beach South | | 5 | | 0 | 0 | 49.038893 | -122.8824 | |  |
| Davis Bay | | 56 | | 2% | 1.79% | 49.440813 | -123.726622 | |  |
| Deep Bay | | 86* | | 4.65% | 0% | 49.466036 | -124.729818 | |  |
| Deep Cove | | 55 | | 9.09% | 1.81% | 49.32639 | -122.943608 | |  |
| Denman Flow Zone | | 84* | | 20%** | 0% | 49.468755 | -124.72909 | |  |
| Dunbar | | 26 | | 3.85% | 0 | 49.273282 | -123.183484 | |  |
| Egmont | | 10 | | 40% | 0% | 49.750105 | -123.930201 | |  |
| Foggy Cove | | 16 | | 0% | 6.25% | 51.65154 | -128.14273 | |  |
| Genoa Bay | | 47 | | 11% | 2.13% | 48.758776 | -123.596877 | |  |
| Gibson's Wharf | | 22 | | 5% | 0% | 49.401618 | -123.504266 | |  |
| Girl in a Wetsuit | | 43* | | 0.00% | 9.30% | 49.303003 | -123.126414 | |  |
| Half Moon Bay | | NA | | NA | occurrence | 49.50937 | -123.94733 | |  |
| Henson Road | | 49 | | 16.33%** | 0% | 49.425548 | -124.646588 | |  |
| Kingfisher | | 48 | | 14.58% | 20.83% | 48.696627 | -123.461576 | |  |
| Kitsilano Point | | 212* | | 1%** | 0.47% | 49.278116 | -123.151149 | |  |
| Lamborghini Cove | | 426* | | 3.76% | 0% | 49.30126 | -122.87349 | |  |
| Lighthouse Park | | NA | | NA | occurrence | 49.33017 | -123.26519 | |  |
| Little Cates | | NA | | occurrence | NA | 49.304929 | -122.948051 | |  |
| Meay Channel | | 53 | | 1.89% | 15.09% | 51.660905 | -128.096704 | |  |
| Navvy Jack Point | | not detected | | NA | NA | 49.327076 | -123.168592 | |  |
| New Brighton | | 16 | | 13% | 0% | 49.291615 | -123.039552 | |  |
| North Beach | | 83 | | 0.00% | 6.02% | 51.66528 | -128.13552 | |  |
| Nova Harvest | | 44 | | 0.00% | 2.27% | 48.831743 | -125.136247 | |  |
| Nudibranch Point | | 124* | | 2% | 2.42% | 48.814503 | -125.171903 | |  |
| Patricia Bay | | 114 | | 1% | 0.88% | 48.655775 | -123.446775 | |  |
| Peter Point | | 106* | | 14% | 1.89% | 48.82161 | -123.586457 | |  |
| Porteau Cove | | 11 | | 0.00% | 0% | 49.559787 | -123.234638 | |  |
| Prospect Point | | 55 | | 0% | 3.64% | 49.313821 | -123.140853 | |  |
| Pruth Lagoon | | 127 | | 13%** | 4.72% | 51.655891 | -128.130308 | |  |
| Quadra | | 18 | | 0% | 11.11% | 50.092796 | -125.261335 | |  |
| Quadra Cove | | 6 | | 0% | 0% | 50.11588 | -125.222595 | |  |
| Roberts Creek | | NA | | Na | occurrence | 49.42062 | -123.65004 | |  |
| Rocky Point Park | | 55 | | 3.63% | 0% | 49.28117 | -122.84848 | |  |
| Sharon Cove | | not detected | | NA | NA | 49.339231 | -123.2222 | |  |
| Shore Access 11 | | 46 | | 0.00% | 0% | 48.868114 | -123.310812 | |  |
| Sidney Kayak | | 24 | | 8.33% | 25% | 48.657068 | -123.393778 | |  |
| Strathcona Lookout | | 260* | | 14.23% | 3.85% | 49.318202 | -122.943706 | |  |
| Strawberry Point | | 89* | | 2% | 4.49% | 48.832512 | -125.128799 | |  |
| Third Beach | | 30 | | 0% | 0% | 49.305879 | -123.1568 | |  |
| Thwaytes Landing | | 18 | | 6% | 0% | 49.371196 | -122.889375 | |  |
| Tower Beach | | 5 | | 0.00% | 0% | 49.271461 | -123.261531 | |  |
| Trafalgar | | 98 | | 0% | 3.06% | 49.274214 | -123.163664 | |  |
| Transfer Beach | | 50 | | 10.00% | 0% | 48.991195 | -123.807486 | |  |
| Tsawwassen Beach | | 65* | | 2% | 0% | 49.021842 | -123.106953 | |  |
| Tuwanek | | 62 | | 4.83% | 0% | 49.54563 | -123.7641 | |  |
| West Beach | | 170 | | 0% | 5.29% | 51.657363 | -128.147823 | |  |
| Whiskey Cove | | 73 | | 10.96% | 0% | 49.320868 | -122.92675 | |  |
| White Rock | | 6 | | 0.00% | 33.33% | 49.02481 | -122.8347 | |  |
|  |  |  |  |  |  |  |  |  |  |

*indicates multiple site visits

** indicates occurrence of *Liriopsis pygmaea*
