# Supplementary material for: Parasites of the hermit crab Pagurus hirsutiusculus; distribution, prevalence, and thermal ecology
Source: PLoS One. 2025 Nov 19;20(11):e0335145. doi: 10.1371/journal.pone.0335145 (PMC12629492; doi:10.1371/journal.pone.0335145)
Supplement: S2 Table — (DOCX) [file pone.0335145.s009.docx]

**Table S2**  *Liriopsis pygmaea* occurrences, prevalence and site information.

| Site | N | *Peltogaster* sp. | *Liriopsis pygmaea* | Date |
| --- | --- | --- | --- | --- |
| Belcarra | 68 | 13 | 1 (7.6%) | 2021-5-16 |
| Denman Flow Zone | 25 | 10 | 1 (10%) | 2021-6-22 |
| Henson Road | Occurrence only | Occurrence only | Occurrence only | 2021-6-26 |
| Pruth Lagoon | 124 | 16 | 2 (12.5%) | 2020-8-22 |
| Kitsilano point | 16 | 2 | 1 (50%) | 2020-05-09 |

*other noted occurrences of *Liriopsis* were just observations, not from prevalence surveys
