# Supplementary material for: Parasites of the hermit crab Pagurus hirsutiusculus; distribution, prevalence, and thermal ecology
Source: PLoS One. 2025 Nov 19;20(11):e0335145. doi: 10.1371/journal.pone.0335145 (PMC12629492; doi:10.1371/journal.pone.0335145)
Supplement: S4 Table — The top portion of this table shows all pairwise comparisons expressed as % similarity among the three clades. The bottom portion of this table compares the three clades to public data from several Peltogaster species obtained from GenBank. (DOCX) [file pone.0335145.s011.docx]

**Table S4.** Average genetic similarity within and among the three clades of *Peltogaster* sp. identified in this study. The top portion of this table shows all pairwise comparisons expressed as % similarity among the three clades. The bottom portion of this table compares the three clades to public data from several *Peltogaster* species obtained from GenBank.

| Taxon | *Peltogaster*  Sp.1  (n=13) | *Peltogaster* sp.2  (n=1) | *Peltogaster boschmai* (n=9) |
| --- | --- | --- | --- |
| *Peltogaster* sp.1 (n=13) | 99.3 | 70.4 | 69.4 |
| *Peltogaster* sp.2 (n=1) | 70.4 | NA | 66.7 |
| *Peltogaster boschmai* (n=9) | 69.1 | 66.7 | 99.1 |
|  |  |  |  |
| MN138416 *Peltogaster boschmai* | 69.8 | 67.1 | 99.1 |
| LC013686 *Peltogaster gracilis* | 69.8 | 90.0 | 66.7 |
| KT209453 *Peltogaster paguri* | 90.3 | 69.6 | 69.1 |
| MN193580 *Peltogaster reticulata* | 77.7 | 69.0 | 71.8 |
| AB602393 *Peltogaster postica* | 79.5 | 67.6 | 68.5 |
| MK604142 *Peltogaster lineatus* | 72.0 | 68.8 | 82.0 |
| KT208786 *Balanus crenatus* | 72.9 | 66.0 | 65.0 |
