# Supplementary material for: Parasites of the hermit crab Pagurus hirsutiusculus; distribution, prevalence, and thermal ecology
Source: PLoS One. 2025 Nov 19;20(11):e0335145. doi: 10.1371/journal.pone.0335145 (PMC12629492; doi:10.1371/journal.pone.0335145)
Supplement: S5 Table — (DOCX) [file pone.0335145.s012.docx]

**Table S5.** The occurrences and prevalence of *Peltogaster paguri* found during the literature review.

| **Prevalence** | **Infected** | **Uninfected** | **Host genus** | **Host Species** | **Site Name** | **Year** | **Latitude** | **Longitude** | **Country** | **Continent** | **Reference** |
| --- | --- | --- | --- | --- | --- | --- | --- | --- | --- | --- | --- |
| 12.6 | 11 | 76 | *Pagurus* | *hirsutiusculus* | Auke Bay | 1996 | 58.36667 | -134.6667 | USA | North_America | Warrenchuk, J. J., T. S. Crustaceana. (2000). PARASITISM BY THE RHIZOCEPHALAN PELTOGASTER PAGURI RATHKE, 1842 AND HYPERPARASITISM BY THE BOPYRID ISOPOD LIRIOPSIS PYGMAEA (RATHKE). 73(8), 971–977. |
| 13.4 | 11 | 71 | *Pagurus* | *hirsutiusculus* | Auke Bay | 1996 | 58.36667 | -134.6667 | USA | North_America | Warrenchuk, J. J., T. S. Crustaceana. (2000). PARASITISM BY THE RHIZOCEPHALAN PELTOGASTER PAGURI RATHKE, 1842 AND HYPERPARASITISM BY THE BOPYRID ISOPOD LIRIOPSIS PYGMAEA (RATHKE). 73(8), 971–977. |
| NA | NA | NA | *Pagurus* | *hirsutiusculus* | Auke Bay | 1996 | 58.36667 | -134.6667 | USA | North_America | Warrenchuk, J. J., T. S. Crustaceana. (2000). PARASITISM BY THE RHIZOCEPHALAN PELTOGASTER PAGURI RATHKE, 1842 AND HYPERPARASITISM BY THE BOPYRID ISOPOD LIRIOPSIS PYGMAEA (RATHKE). 73(8), 971–977. |
| 0 | 0 | 0 | *Pagarus* | *acadianus* | Frenchman Bay | 1939 | 44.432688 | -68.290556 | USA | North_America | Reinhard, E. G. (1939). Rediscovery of the Rhizocephalan Peltogaster paguri on the north american coast. Science 89(2300), 80–81. |
| NA | 24 | NA | *Pagurus* | *bernhardus* | Starcross | 1884 | 50.627306 | -3.447022 | UK | Europe | London, H. B. J. O. T. L. S. O., (1933). (n.d.). The Rhizocephala in the collection of the British Museum. Limnology and Oceanography. |
| NA | 2 | NA | *Pagurus* | *bernhardus* | Off Sunderland | 1863 | 54.908841 | -1.351054 | UK | Europe | London, H. B. J. O. T. L. S. O., (1933). (n.d.). The Rhizocephala in the collection of the British Museum. Limnology and Oceanography. |
| NA | 1 | NA | *Pagurus* | *bernhardus* | Firth of Forth | 1861 | 56.01569 | -3.357225 | UK | Europe | London, H. B. J. O. T. L. S. O., 1933. (n.d.). The Rhizocephala in the collection of the British Museum. Limnology and Oceanography. |
| NA | 1 | NA | *Pagurus* | *pubescens* | Molde | 1858 | 62.736188 | 7.161378 | Norway | Europe | Lilljeborg, W. (1858). XXXIV. On the Genera Peltogaster and Liriope of Rathke. Page 173 in The Annals and magazine of natural history; zoology, botany and geology. |
| NA | 1 | NA | *Pagurus* | *pubescens* | Eastport | 1866 | 44.905151 | -66.983898 | USA | North_America | Reinhard, E. G. (1942). Studies on the Life History and Host-Parasite Relationship of Peltogaster Paguri. Biological Bulletin 83(2), 401–415. |
| 25.2 | 86 | 255 | *Pagurus* | *pubescens* | Googin's Ledge | 1938 | 44.445547 | -68.287987 | USA | North_America | Reinhard, E. G. (1942). Studies on the Life History and Host-Parasite Relationship of Peltogaster Paguri. Biological Bulletin 83(2), 401–415. |
| 10.3 | 89 | 777 | *Pagurus* | *pubescens* | Googin's Ledge | 1939 | 44.445547 | -68.287987 | USA | North_America | Reinhard, E. G. (1942). Studies on the Life History and Host-Parasite Relationship of Peltogaster Paguri. Biological Bulletin 83(2), :401–415. |
| 15 | 89 | 503 | *Pagurus* | *pubescens* | Googin's Ledge | 1940 | 44.445547 | -68.287987 | USA | North_America | Reinhard, E. G. (1942). Studies on the Life History and Host-Parasite Relationship of Peltogaster Paguri. Biological Bulletin 83(2), 401–415. |
| 15.9 | 103 | 542 | *Pagurus* | *pubescens* | Googin's Ledge | 1941 | 44.445547 | -68.287987 | USA | North_America | Reinhard, E. G. (1942). Studies on the Life History and Host-Parasite Relationship of Peltogaster Paguri. Biological Bulletin 83(2).401–415. |
| 7.5 | 22 | 269 | *Pagurus* | *pubescens* | Frenchman Bay | 1939 | 44.432688 | -68.290556 | USA | North_America | Reinhard, E. G. (1942). Studies on the Life History and Host-Parasite Relationship of Peltogaster Paguri. Biological Bulletin 83(2), 401–415. |
| 10.3 | 9 | 78 | *Pagurus* | *pubescens* | Frenchman Bay | 1940 | 44.432688 | -68.290556 | USA | North_America | Reinhard, E. G. (1942). Studies on the Life History and Host-Parasite Relationship of Peltogaster Paguri. Biological Bulletin 83(2), 401–415. |
| 9.3 | 25 | 245 | *Pagurus* | *pubescens* | Frenchman Bay | 1941 | 44.432688 | -68.290556 | USA | North_America | Reinhard, E. G. (1942). Studies on the Life History and Host-Parasite Relationship of Peltogaster Paguri. Biological Bulletin 83(2), 401–415. |
| NA | NA | NA | *Pagurus* | *pubescens* | Frenchman Bay | 1938 | 44.445547 | -68.287987 | USA | North_America | Reinhard, E. G. (1942). Studies on the Life History and Host-Parasite Relationship of Peltogaster Paguri. Biological Bulletin 83(2), 401–415. |
| NA | 6 | NA | *Pagurus* | *capillatus* | Coal Harbor | 1872 | 55.336944 | -160.604167 | USA | North_America | Reinhard, E. G. (1944). Rhizocephalan parasites of hermit crabs from the Northwest Pacific. Journal of the Washington Academy of Sciences, 34(2), 49–58. |
| NA | 1 | NA | *Pagurus* | *hirsutiusculus* | Unalaska | 1937 | 53.883535 | -166.53301 | USA | North_America | Reinhard, E. G. (1944). Rhizocephalan parasites of hermit crabs from the Northwest Pacific. Journal of the Washington Academy of Sciences, 34(2), 49–58. |
| 2.4 | 20 | 800 | *Pagurus* | *bernhardus* | southern North Sea | 1974 | 53.793668 | 2.961472 | Netherlands | Europe | Adema, J. P. H. M., & Huwae, P. H. M. (1982). New and supplementary records of marine isopoda for the Netherlands and the southern North Sea since 1956, with a note on Peltogaster Paguri (Crustacea, Cirripedia). Zoologische Bijdragen, 28(3), 33–57. |
| NA | 1 | NA | *Pagurus* | *bernhardus* | southern North Sea | 1974 | 55.433333 | 0.483333 | Netherlands | Europe | Adema, J. P. H. M., & Huwae, P. H. M. (1982). New and supplementary records of marine isopoda for the Netherlands and the southern North Sea since 1956, with a note on Peltogaster Paguri (Crustacea, Cirripedia). Zoologische Bijdragen, 28(3), 33–57. |
| NA | 1 | NA | *Pagurus* | *bernhardus* | southern North Sea | 1974 | 53.583333 | 4.401389 | Netherlands | Europe | Adema, J. P. H. M., & Huwae, P. H. M. (1982). New and supplementary records of marine isopoda for the Netherlands and the southern North Sea since 1956, with a note on Peltogaster Paguri (Crustacea, Cirripedia). Zoologische Bijdragen, 28(3), 33–57. |
| NA | 1 | NA | *Pagurus* | *bernhardus* | southern North Sea | 1975 | 53.816667 | 4.1 | Netherlands | Europe | Adema, J. P. H. M., & Huwae, P. H. M. (1982). New and supplementary records of marine isopoda for the Netherlands and the southern North Sea since 1956, with a note on Peltogaster Paguri (Crustacea, Cirripedia). Zoologische Bijdragen, 28(3), 33–57. |
| NA | 1 | NA | *Pagurus* | *bernhardus* | southern North Sea | 1975 | 54.483333 | 3.55 | Netherlands | Europe | Adema, J. P. H. M., & Huwae, P. H. M. (1982). New and supplementary records of marine isopoda for the Netherlands and the southern North Sea since 1956, with a note on Peltogaster Paguri (Crustacea, Cirripedia). Zoologische Bijdragen, 28(3), 33–57. |
| NA | 1 | NA | *Pagurus* | *bernhardus* | southern North Sea | 1975 | 53.266667 | 3.816667 | Netherlands | Europe | Adema, J. P. H. M., & Huwae, P. H. M. (1982). New and supplementary records of marine isopoda for the Netherlands and the southern North Sea since 1956, with a note on Peltogaster Paguri (Crustacea, Cirripedia). Zoologische Bijdragen, 28(3), 33–57. |
| NA | 1 | NA | *Pagurus* | *bernhardus* | southern North Sea | 1975 | 53.283333 | 2.516667 | Netherlands | Europe | Adema, J. P. H. M., & Huwae, P. H. M. (1982). New and supplementary records of marine isopoda for the Netherlands and the southern North Sea since 1956, with a note on Peltogaster Paguri (Crustacea, Cirripedia). Zoologische Bijdragen, 28(3), 33–57. |
| NA | 1 | NA | *Pagurus* | *bernhardus* | southern North Sea | 1975 | 53.483333 | 2.95 | Netherlands | Europe | Adema, J. P. H. M., & Huwae, P. H. M. (1982). New and supplementary records of marine isopoda for the Netherlands and the southern North Sea since 1956, with a note on Peltogaster Paguri (Crustacea, Cirripedia). Zoologische Bijdragen, 28(3), 33–57. |
| NA | 1 | NA | *Pagurus* | *bernhardus* | southern North Sea | 1975 | 52.633333 | 3.866667 | Netherlands | Europe | Adema, J. P. H. M., & Huwae, P. H. M. (1982). New and supplementary records of marine isopoda for the Netherlands and the southern North Sea since 1956, with a note on Peltogaster Paguri (Crustacea, Cirripedia). Zoologische Bijdragen, 28(3), 33–57. |
| NA | 1 | NA | *Pagurus* | *bernhardus* | southern North Sea | 1975 | 52.55 | 3.933333 | Netherlands | Europe | Adema, J. P. H. M., & Huwae, P. H. M. (1982). New and supplementary records of marine isopoda for the Netherlands and the southern North Sea since 1956, with a note on Peltogaster Paguri (Crustacea, Cirripedia). Zoologische Bijdragen, 28(3), 33–57. |
| NA | 2 | NA | *Pagurus* | *bernhardus* | southern North Sea | 1975 | 52.533333 | 3.483333 | Netherlands | Europe | Adema, J. P. H. M., & Huwae, P. H. M. (1982). New and supplementary records of marine isopoda for the Netherlands and the southern North Sea since 1956, with a note on Peltogaster Paguri (Crustacea, Cirripedia). Zoologische Bijdragen, 28(3), 33–57. |
| NA | 1 | NA | *Pagurus* | *bernhardus* | southern North Sea | 1975 | 52.4 | 2.75 | Netherlands | Europe | Adema, J. P. H. M., & Huwae, P. H. M. (1982). New and supplementary records of marine isopoda for the Netherlands and the southern North Sea since 1956, with a note on Peltogaster Paguri (Crustacea, Cirripedia). Zoologische Bijdragen, 28(3), 33–57. |
| NA | 3 | NA | *Pagurus* | *bernhardus* | southern North Sea | 1975 | 52.883333 | 2.766667 | Netherlands | Europe | Adema, J. P. H. M., & Huwae, P. H. M. (1982). New and supplementary records of marine isopoda for the Netherlands and the southern North Sea since 1956, with a note on Peltogaster Paguri (Crustacea, Cirripedia). Zoologische Bijdragen, 28(3), 33–57. |
| NA | 1 | NA | *Pagurus* | *bernhardus* | southern North Sea | 1975 | 52.966667 | 2.966667 | Netherlands | Europe | Adema, J. P. H. M., & Huwae, P. H. M. (1982). New and supplementary records of marine isopoda for the Netherlands and the southern North Sea since 1956, with a note on Peltogaster Paguri (Crustacea, Cirripedia). Zoologische Bijdragen, 28(3), 33–57. |
| NA | 1 | NA | *Pagurus* | *bernhardus* | southern North Sea | 1975 | 53.016667 | 3.183333 | Netherlands | Europe | Adema, J. P. H. M., & Huwae, P. H. M. (1982). New and supplementary records of marine isopoda for the Netherlands and the southern North Sea since 1956, with a note on Peltogaster Paguri (Crustacea, Cirripedia). Zoologische Bijdragen, 28(3), 33–57. |
| NA | 1 | NA | *Pagurus* | *bernhardus* | southern North Sea | 1976 | 53.833333 | 4.05 | Netherlands | Europe | Adema, J. P. H. M., & Huwae, P. H. M. (1982). New and supplementary records of marine isopoda for the Netherlands and the southern North Sea since 1956, with a note on Peltogaster Paguri (Crustacea, Cirripedia). Zoologische Bijdragen, 28(3), 33–57. |
| NA | 2 | NA | *Pagurus* | *bernhardus* | southern North Sea | 1976 | 52.95 | 2.983333 | Netherlands | Europe | Adema, J. P. H. M., & Huwae, P. H. M. (1982). New and supplementary records of marine isopoda for the Netherlands and the southern North Sea since 1956, with a note on Peltogaster Paguri (Crustacea, Cirripedia). Zoologische Bijdragen, 28(3), 33–57. |
| NA | 1 | NA | *Pagurus* | *bernhardus* | southern North Sea | 1976 | 52.883333 | 2.766667 | Netherlands | Europe | Adema, J. P. H. M., & Huwae, P. H. M. (1982). New and supplementary records of marine isopoda for the Netherlands and the southern North Sea since 1956, with a note on Peltogaster Paguri (Crustacea, Cirripedia). Zoologische Bijdragen, 28(3), 33–57. |
| NA | 1 | NA | *Pagurus* | *bernhardus* | southern North Sea | 1976 | 52.8 | 2.566667 | Netherlands | Europe | Adema, J. P. H. M., & Huwae, P. H. M. (1982). New and supplementary records of marine isopoda for the Netherlands and the southern North Sea since 1956, with a note on Peltogaster Paguri (Crustacea, Cirripedia). Zoologische Bijdragen, 28(3), 33–57. |
| NA | 1 | NA | *Pagurus* | *bernhardus* | southern North Sea | 1976 | 52.683333 | 2.633333 | Netherlands | Europe | Adema, J. P. H. M., & Huwae, P. H. M. (1982). New and supplementary records of marine isopoda for the Netherlands and the southern North Sea since 1956, with a note on Peltogaster Paguri (Crustacea, Cirripedia). Zoologische Bijdragen, 28(3), 33–57. |
| NA | 1 | NA | *Pagurus* | *bernhardus* | southern North Sea | 1976 | 52.55 | 2.716667 | Netherlands | Europe | Adema, J. P. H. M., & Huwae, P. H. M. (1982). New and supplementary records of marine isopoda for the Netherlands and the southern North Sea since 1956, with a note on Peltogaster Paguri (Crustacea, Cirripedia). Zoologische Bijdragen, 28(3), 33–57. |
| NA | 4 | NA | *Pagurus* | *bernhardus* | southern North Sea | 1976 | 52.4 | 2.783333 | Netherlands | Europe | Adema, J. P. H. M., & Huwae, P. H. M. (1982). New and supplementary records of marine isopoda for the Netherlands and the southern North Sea since 1956, with a note on Peltogaster Paguri (Crustacea, Cirripedia). Zoologische Bijdragen, 28(3), 33–57. |
| NA | 2 | NA | *Pagurus* | *bernhardus* | southern North Sea | 1976 | 52.516667 | 3.033333 | Netherlands | Europe | Adema, J. P. H. M., & Huwae, P. H. M. (1982). New and supplementary records of marine isopoda for the Netherlands and the southern North Sea since 1956, with a note on Peltogaster Paguri (Crustacea, Cirripedia). Zoologische Bijdragen, 28(3), 33–57. |
| NA | 1 | NA | *Pagurus* | *bernhardus* | southern North Sea | 1976 | 52.433333 | 3.716667 | Netherlands | Europe | Adema, J. P. H. M., & Huwae, P. H. M. (1982). New and supplementary records of marine isopoda for the Netherlands and the southern North Sea since 1956, with a note on Peltogaster Paguri (Crustacea, Cirripedia). Zoologische Bijdragen, 28(3), 33–57. |
| NA | 2 | NA | *Pagurus* | *bernhardus* | southern North Sea | 1976 | 52.451389 | 4.2 | Netherlands | Europe | Adema, J. P. H. M., & Huwae, P. H. M. (1982). New and supplementary records of marine isopoda for the Netherlands and the southern North Sea since 1956, with a note on Peltogaster Paguri (Crustacea, Cirripedia). Zoologische Bijdragen, 28(3), 33–57. |
| NA | 1 | NA | *Pagurus* | *bernhardus* | southern North Sea | 1976 | 52.734722 | 3.118056 | Netherlands | Europe | Adema, J. P. H. M., & Huwae, P. H. M. (1982). New and supplementary records of marine isopoda for the Netherlands and the southern North Sea since 1956, with a note on Peltogaster Paguri (Crustacea, Cirripedia). Zoologische Bijdragen, 28(3), 33–57. |
| NA | 1 | NA | *Pagurus* | *bernhardus* | southern North Sea | 1976 | 53.716667 | 4.233333 | Netherlands | Europe | Adema, J. P. H. M., & Huwae, P. H. M. (1982). New and supplementary records of marine isopoda for the Netherlands and the southern North Sea since 1956, with a note on Peltogaster Paguri (Crustacea, Cirripedia). Zoologische Bijdragen, 28(3), 33–57. |
| NA | 1 | NA | *Pagurus* | *bernhardus* | southern North Sea | 1977 | 53.793668 | 2.961472 | Netherlands | Europe | Adema, J. P. H. M., & Huwae, P. H. M. (1982). New and supplementary records of marine isopoda for the Netherlands and the southern North Sea since 1956, with a note on Peltogaster Paguri (Crustacea, Cirripedia). Zoologische Bijdragen, 28(3), 33–57. |
| NA | NA | NA | *Pagurus* | *bernhardus* | southern North Sea | 1974 | 55.316667 | 0.7 | Netherlands | Europe | Adema, J. P. H. M., & Huwae, P. H. M. (1982). New and supplementary records of marine isopoda for the Netherlands and the southern North Sea since 1956, with a note on Peltogaster Paguri (Crustacea, Cirripedia). Zoologische Bijdragen, 28(3), 33–57. |
| NA | 3 | NA | *Pagurus* | *pubescens* | off Newfoundland | 1885 | 45.733333 | -49.75 | Canada | North_America | Reinhard, E. (1946). Rhizocephala from New England and the Grand Banks. Journal of The Washington Academy of Sciences, 36(4), 127–131. |
| NA | 8 | NA | *Pagurus* | *pubescens* | off Nova Scotia | 1885 | 44.466667 | -60.254167 | Canada | North_America | Reinhard, E. (1946). Rhizocephala from New England and the Grand Banks. Journal of The Washington Academy of Sciences, 36(4), 127–131. |
| NA | 2 | NA | *Pagurus* | *pubescens* | off Nova Scotia | 1885 | 43.616667 | -49.941667 | Canada | North_America | Reinhard, E. (1946). Rhizocephala from New England and the Grand Banks. Journal of The Washington Academy of Sciences, 36(4), 127–131. |
| NA | 1 | NA | *Pagurus* | *pubescens* | Off Cape Cod | 1883 | 42.016667 | -68.008333 | USA | North_America | Reinhard, E. (1946). Rhizocephala from New England and the Grand Banks. Journal of The Washington Academy of Sciences, 36(4), 127–131. |
| 6.5 | NA | NA | *Anapagurus* | *laevis* | BIOFAR area | 1987 | 62.059406 | -6.830135 | Faroe Islands | Europe | Lange, S., & Båmstedt, U. (1999). Rhizocephala (Crustacea, Cirripedia) of the Faroe Islands. Sarsia, 84(1), 79–83.<https://doi.org/10.1080/00364827.1999.10420453> |
| 3 | NA | NA | *Pagurus* | *bernhardus* | BIOFAR area | 1987 | 62.059406 | -6.830135 | Faroe Islands | Europe | Lange, S., & Båmstedt, U. (1999). Rhizocephala (Crustacea, Cirripedia) of the Faroe Islands. Sarsia, 84(1), 79–83.<https://doi.org/10.1080/00364827.1999.10420454> |
| 0.5 | NA | NA | *Pagurus* | *pubescens* | BIOFAR area | 1987 | 62.059406 | -6.830135 | Faroe Islands | Europe | Lange, S., & Båmstedt, U. (1999). Rhizocephala (Crustacea, Cirripedia) of the Faroe Islands. Sarsia, 84(1), 79–83.<https://doi.org/10.1080/00364827.1999.10420455> |
| NA | 2 | NA | *Anapagurus* | *laevis* | Stn 006 | 1987 | 62.253667 | -5.586167 | Faroe Islands | Europe | Lange, S., & Båmstedt, U. (1999). Rhizocephala (Crustacea, Cirripedia) of the Faroe Islands. Sarsia, 84(1), 79–83.<https://doi.org/10.1080/00364827.1999.10420456> |
| NA | 1 | NA | *Pagurus* | *pubescens* | Stn 006 | 1987 | 62.253667 | -5.586167 | Faroe Islands | Europe | Lange, S., & Båmstedt, U. (1999). Rhizocephala (Crustacea, Cirripedia) of the Faroe Islands. Sarsia, 84(1), 79–83.<https://doi.org/10.1080/00364827.1999.10420457> |
| NA | 3 | NA | *Anapagurus* | *laevis* | Stn 007 | 1987 | 62.266667 | -5.633333 | Faroe Islands | Europe | Lange, S., & Båmstedt, U. (1999). Rhizocephala (Crustacea, Cirripedia) of the Faroe Islands. Sarsia, 84(1), 79–83.<https://doi.org/10.1080/00364827.1999.10420458> |
| NA | 1 | NA | *Anapagurus* | *laevis* | Stn 100 | 1987 | 61.579333 | -6.284167 | Faroe Islands | Europe | Lange, S., & Båmstedt, U. (1999). Rhizocephala (Crustacea, Cirripedia) of the Faroe Islands. Sarsia, 84(1), 79–83.<https://doi.org/10.1080/00364827.1999.10420459> |
| NA | 1 | NA | *Pagurus* | *pubescens* | Stn 304 | 1988 | 60.178333 | -9.803333 | Faroe Islands | Europe | Lange, S., & Båmstedt, U. (1999). Rhizocephala (Crustacea, Cirripedia) of the Faroe Islands. Sarsia, 84(1), 79–83.<https://doi.org/10.1080/00364827.1999.10420460> |
| NA | 1 | NA | *Pagurus* | *pubescens* | Stn 324 | 1988 | 60.581667 | -9.363333 | Faroe Islands | Europe | Lange, S., & Båmstedt, U. (1999). Rhizocephala (Crustacea, Cirripedia) of the Faroe Islands. Sarsia, 84(1), 79–83.<https://doi.org/10.1080/00364827.1999.10420461> |
| NA | 1 | NA | *Pagurus* | *bernhardus* | Stn 351 | 1988 | 62.248333 | -7.768333 | Faroe Islands | Europe | Lange, S., & Båmstedt, U. (1999). Rhizocephala (Crustacea, Cirripedia) of the Faroe Islands. Sarsia, 84(1), 79–83.<https://doi.org/10.1080/00364827.1999.10420462> |
| NA | 1 | Na | *Anapagurus* | *laevis* | Stn 356 | 1988 | 62.423333 | -5.326667 | Faroe Islands | Europe | Lange, S., & Båmstedt, U. (1999). Rhizocephala (Crustacea, Cirripedia) of the Faroe Islands. Sarsia, 84(1), 79–83.<https://doi.org/10.1080/00364827.1999.10420463> |
| NA | 1 | NA | *Anapagurus* | *laevis* | Stn 357 | 1988 | 62.068333 | -4.726667 | Faroe Islands | Europe | Lange, S., & Båmstedt, U. (1999). Rhizocephala (Crustacea, Cirripedia) of the Faroe Islands. Sarsia, 84(1), 79–83.<https://doi.org/10.1080/00364827.1999.10420464> |
| NA | 1 | NA | *Anapagurus* | *laevis* | Stn 359 | 1988 | 61.955 | -4.366667 | Faroe Islands | Europe | Lange, S., & Båmstedt, U. (1999). Rhizocephala (Crustacea, Cirripedia) of the Faroe Islands. Sarsia, 84(1), 79–83.<https://doi.org/10.1080/00364827.1999.10420465> |
| NA | 1 | NA | *Pagurus* | *pubescens* | Stn 364 | 1988 | 62.248333 | -5.573333 | Faroe Islands | Europe | Lange, S., & Båmstedt, U. (1999). Rhizocephala (Crustacea, Cirripedia) of the Faroe Islands. Sarsia, 84(1), 79–83.<https://doi.org/10.1080/00364827.1999.10420466> |
| NA | 1 | NA | *Pagurus* | *pubescens* | Stn 368 | 1988 | 62.363333 | -6.736667 | Faroe Islands | Europe | Lange, S., & Båmstedt, U. (1999). Rhizocephala (Crustacea, Cirripedia) of the Faroe Islands. Sarsia, 84(1), 79–83.<https://doi.org/10.1080/00364827.1999.10420467> |
| NA | 1 | NA | *Pagurus* | *bernhardus* | Stn 371 | 1988 | 62.283333 | -6.916667 | Faroe Islands | Europe | Lange, S., & Båmstedt, U. (1999). Rhizocephala (Crustacea, Cirripedia) of the Faroe Islands. Sarsia, 84(1), 79–83.<https://doi.org/10.1080/00364827.1999.10420468> |
| NA | 1 | NA | *Anapagurus* | *laevis* | Stn 456 | 1989 | 62.633 | -7.2575 | Faroe Islands | Europe | Lange, S., & Båmstedt, U. (1999). Rhizocephala (Crustacea, Cirripedia) of the Faroe Islands. Sarsia, 84(1), 79–83.<https://doi.org/10.1080/00364827.1999.10420469> |
| NA | 1 | NA | *Pagurus* | *bernhardus* | Stn 606 | 1990 | 61.258333 | -6.286667 | Faroe Islands | Europe | Lange, S., & Båmstedt, U. (1999). Rhizocephala (Crustacea, Cirripedia) of the Faroe Islands. Sarsia, 84(1), 79–83.<https://doi.org/10.1080/00364827.1999.10420470> |
| NA | 1 | NA | *Anapagurus* | *laevis* | Stn 606 | 1990 | 61.258333 | -6.286667 | Faroe Islands | Europe | Lange, S., & Båmstedt, U. (1999). Rhizocephala (Crustacea, Cirripedia) of the Faroe Islands. Sarsia, 84(1), 79–83.<https://doi.org/10.1080/00364827.1999.10420471> |
| 0.3 | 9 |  | *Pagurus* | *bernhardus* | Roscoff | 1960??? | 48.727089 | -3.987611 | France | Europe | Bourdon, R. (1963). Epicarides et Rhizocéphales de Roscoff. Cahiers de Biologie Marine, 4(4), 415–434. |
| 0.1 | 1 |  | *Pagurus* | *cuanensis* | Roscoff | 1960??? | 48.727089 | -3.987611 | France | Europe | Bourdon, R. (1963). Epicarides et Rhizocéphales de Roscoff. Cahiers de Biologie Marine, 4(4), 415–434. |
| 0.2 | 3 | 1787 | *Pagurus* | *bernhardus* | Elbow | 1983 | 55.7225 | -4.983167 | UK | Europe | Moore, P. G., & Nickell, T. D. (1990). A Note on the Occurrence of Parasitic Crustacea on Certain Epibenthic Hermit Crabs and Prawns (Decapoda Anomura et Caridea) from the Clyde Sea, Scotland. Crustaceana, 59(3), 303–306. |
| 0 | 0 | 4 | *Anapagurus* | *laevis* | Elbow | 1983 | 55.7225 | -4.983167 | UK | Europe | Moore, P. G., & Nickell, T. D. (1990). A Note on the Occurrence of Parasitic Crustacea on Certain Epibenthic Hermit Crabs and Prawns (Decapoda Anomura et Caridea) from the Clyde Sea, Scotland. Crustaceana, 59(3), 303–306. |
| 1.2 | 1 | 84 | *Pagurus* | *bernhardus* | Clach | 1983 | 55.746833 | -4.9375 | UK | Europe | Moore, P. G., & Nickell, T. D. (1990). A Note on the Occurrence of Parasitic Crustacea on Certain Epibenthic Hermit Crabs and Prawns (Decapoda Anomura et Caridea) from the Clyde Sea, Scotland. Crustaceana, 59(3), 303–306. |
| NA | 1 | NA | *Pagurus* | *pubescens* | Henley Harbour | 1882 | 52.011634 | -55.869607 | Canada | North_America | Smith, S. I. (1883). List of the Crustacea dredged on the coast of Labrador by the expedition under the direction of W. A. Stearns, in 1882. Proceedings of the United States National Museum, 6(374), 218–222.<https://doi.org/10.1126/science.9.231.776> |
| NA | 1 | NA | *Pagurus* | *bernhardus* | Hunstanton Scaup | 1963 | 52.935667 | 0.481113 | UK | Europe | Hamond, R. (1997). The Cirripedia, Branchiura and marine Cladocera of Norfolk. Transactions of the Norfolk and Norwich Naturalists’ Society, 31(1), 56–57. |
| NA | 1 | NA | *Pagurus* | *bernhardus* | Hunstanton Scaup | 1963 | 52.935667 | 0.481113 | UK | Europe | Hamond, R. (1997). The Cirripedia, Branchiura and marine Cladocera of Norfolk. Transactions of the Norfolk and Norwich Naturalists’ Society, 31(1), 56–57. |
| NA | 1 | NA | *Pagurus* | *bernhardus* | Freshes Lays | 1963 | 52.973836 | 0.956362 | UK | Europe | Hamond, R. (1997). The Cirripedia, Branchiura and marine Cladocera of Norfolk. Transactions of the Norfolk and Norwich Naturalists’ Society, 31(1), 56–57. |
| NA | 1 | NA | *Pagurus* | *bernhardus* | West Runton | 1964 | 52.942293 | 1.244453 | UK | Europe | Hamond, R. (1997). The Cirripedia, Branchiura and marine Cladocera of Norfolk. Transactions of the Norfolk and Norwich Naturalists’ Society, 31(1), 56–57. |
| NA | 1 | NA | *Pagurus* | *bernhardus* | Slip Pier Beach | 1955 | 51.703347 | -5.15071 | UK | Europe | Bassindale, R., & Barret, J. H. (1955). The Dale Fort Marine Fauna. 29, 270.<http://www.biodiversitylibrary.org/item/178299> |
| NA | 1 | NA | *Pagurus* | *bernhardus* | Stat 24 | 1928 | 56.951034 | 11.307781 | Denmark | Europe | Boschma, H. (1928). Rhizocephala of the North Atlantic Region. The Danish Ingolf Expedition, 3c, 1–49. |
| NA | 1 | NA | *Pagurus* | *bernhardus* | Stat 114 | 1884 | 57.3134 | 10.987986 | Denmark | Europe | Boschma, H. (1928). Rhizocephala of the North Atlantic Region. The Danish Ingolf Expedition, 3c, 1–49. |
| NA | 2 | NA | *Pagurus* | *bernhardus* | Stat 115 | 1884 | 57.3134 | 10.987986 | Denmark | Europe | Boschma, H. (1928). Rhizocephala of the North Atlantic Region. The Danish Ingolf Expedition, 3c, 1–49. |
| NA | 1 | NA | *Pagurus* | *bernhardus* | Stat 226 | 1928 | 56.951034 | 11.307781 | Denmark | Europe | Boschma, H. (1928). Rhizocephala of the North Atlantic Region. The Danish Ingolf Expedition, 3c, 1–49. |
| NA | 1 | NA | *Pagurus* | *bernhardus* | Frederikshavn | 1884 | 57.459984 | 10.540263 | Denmark | Europe | Boschma, H. (1928). Rhizocephala of the North Atlantic Region. The Danish Ingolf Expedition, 3c, 1–49. |
| NA | 1 | NA | *Pagurus* | *bernhardus* | Hellebaek | 1864 | 56.069328 | 12.56127 | Denmark | Europe | Boschma, H. (1928). Rhizocephala of the North Atlantic Region. The Danish Ingolf Expedition, 3c, 1–49. |
| NA | 1 | NA | *Pagurus* | *bernhardus* | off Aalbaek | 1928 | 57.591544 | 10.53211 | Denmark | Europe | Boschma, H. (1928). Rhizocephala of the North Atlantic Region. The Danish Ingolf Expedition, 3c, 1–49. |
| NA | 1 | NA | *Pagurus* | *bernhardus* | off Anholt | 1928 | 56.74101 | 11.560751 | Denmark | Europe | Boschma, H. (1928). Rhizocephala of the North Atlantic Region. The Danish Ingolf Expedition, 3c, 1–49. |
| NA | 1 | NA | *Pagurus* | *bernhardus* | Ymuiden | 1897 | 52.45706 | 4.551375 | Netherlands | Europe | Boschma, H. (1928). Rhizocephala of the North Atlantic Region. The Danish Ingolf Expedition, 3c, 1–49. |
| NA | 1 | NA | *Pagurus* | *bernhardus* | Helder | 1928 | 52.963952 | 4.753456 | Netherlands | Europe | Boschma, H. (1928). Rhizocephala of the North Atlantic Region. The Danish Ingolf Expedition, 3c, 1–49. |
| NA | 1 | NA | *Pagurus* | *cuanensis* | Stat. 56 | 1928 | 56.951034 | 11.307781 | Denmark | Europe | Boschma, H. (1928). Rhizocephala of the North Atlantic Region. The Danish Ingolf Expedition, 3c, 1–49. |
| NA | 1 | NA | *Pagurus* | *cuanensis* | off Læsø | 1923 | 57.3134 | 10.987986 | Denmark | Europe | Boschma, H. (1928). Rhizocephala of the North Atlantic Region. The Danish Ingolf Expedition, 3c, 1–49. |
| NA | 1 | NA | *Anapagurus* | *chrioacanthus* | Stat. 243 | 1928 | 56.951034 | 11.307781 | Denmark | Europe | Boschma, H. (1928). Rhizocephala of the North Atlantic Region. The Danish Ingolf Expedition, 3c, 1–49. |
| 11.4 | 4 | 31 | *Pagurus* | *bernhardus* | Koksijde | 1981 | 51.128251 | 2.647765 | Belgium | Europe | Desender, K. (1981). Heremietkreeften en hun parasieten. De Strandvlo, 1, 60–64. |
| 0 | 0 | 47 | *Pagurus* | *bernhardus* | Koksijde | 1981 | 51.128251 | 2.647765 | Belgium | Europe | Desender, K. (1981). Heremietkreeften en hun parasieten. De Strandvlo, 1, 60–64. |
| 5.1 | 2 | 37 | *Pagurus* | *bernhardus* | Koksijde | 1981 | 51.128251 | 2.647765 | Belgium | Europe | Desender, K. (1981). Heremietkreeften en hun parasieten. De Strandvlo, 1, 60–64. |
| 57 | 4 | 3 | *Pagurus* | *bernhardus* | Koksijde | 1981 | 51.128251 | 2.647765 | Belgium | Europe | Desender, K. (1981). Heremietkreeften en hun parasieten. De Strandvlo, 1, 60–64. |
| 18 | 150 | 682 | *Pagurus* | *arcuatus* | St. Chads | 1971 | 48.694784 | -53.745263 | Canada | North_America | Squires, H. J., Ennis, G. P., & Dawe, G. (2001). On biology of two sympatric species of Hermit Crab (Crustacea, Decapoda, Paguridae) at St. Chads, Newfoundland. NAFO Scientific Council Studies, 34, 7–17. |
| 0.2 | 1 | 473 | *Pagurus* | *acadianus* | St. Chads | 1971 | 48.694784 | -53.745263 | Canada | North_America | Squires, H. J., Ennis, G. P., & Dawe, G. (2001). On biology of two sympatric species of Hermit Crab (Crustacea, Decapoda, Paguridae) at St. Chads, Newfoundland. NAFO Scientific Council Studies, 34, 7–17. |
| 4.7 | 14 | 283 | *Pagurus* | *maculosus* | Sanriku | 1995 | 39.097321 | 141.86773 | Japan | Asia | Nagasawa, K., Lützen, J., & Kado, R. (1996). Parasitic Cirripedia (Rhizocephala) and Isopoda from Brachyuran and Anomuran Crabs of the Pacific Coast of Northern Honshu, Japan. Bulletin of the Biogeographical Society of Japan, 51(2), 1–6. |
| 0 | 0 | 87 | *Pagurus* | *filholi* | Sanriku | 1995 | 39.097321 | 141.86773 | Japan | Asia | Nagasawa, K., Lützen, J., & Kado, R. (1996). Parasitic Cirripedia (Rhizocephala) and Isopoda from Brachyuran and Anomuran Crabs of the Pacific Coast of Northern Honshu, Japan. Bulletin of the Biogeographical Society of Japan, 51(2), 1–6. |
| 0 | 0 | 3 | *Pagurus* | *middendorffii* | Sanriku | 1995 | 39.097321 | 141.86773 | Japan | Asia | Nagasawa, K., Lützen, J., & Kado, R. (1996). Parasitic Cirripedia (Rhizocephala) and Isopoda from Brachyuran and Anomuran Crabs of the Pacific Coast of Northern Honshu, Japan. Bulletin of the Biogeographical Society of Japan, 51(2), 1–6. |
| 0 | 0 | 11107 | *Pagurus* | *minutus* | Vostok Bay | 2014 | 42.880832 | 132.742867 | Russia | Asia | Kornienko, E. S., Korn, O. M., & Selin, N. I. (2018). The Parasitic Fauna of Common Species of Hermit Crabs of Vostok Bay (Sea of Japan). Russian Journal of Marine Biology, 44(2), 94–99.<https://doi.org/10.1134/S1063074018020062> |
| 6.5 | 176 | 176 | *Pagurus* | *brachiomastus* | Vostok Bay | 2014 | 42.880832 | 132.742867 | Russia | Asia | Kornienko, E. S., Korn, O. M., & Selin, N. I. (2018). The Parasitic Fauna of Common Species of Hermit Crabs of Vostok Bay (Sea of Japan). Russian Journal of Marine Biology, 44(2), 94–99.<https://doi.org/10.1134/S1063074018020063> |
| 0 | 0 | 231 | *Pagurus* | *pectinatus* | Vostok Bay | 2014 | 42.880832 | 132.742867 | Russia | Asia | Kornienko, E. S., Korn, O. M., & Selin, N. I. (2018). The Parasitic Fauna of Common Species of Hermit Crabs of Vostok Bay (Sea of Japan). Russian Journal of Marine Biology, 44(2), 94–99.<https://doi.org/10.1134/S1063074018020064> |
| 0.4 | 3 | 805 | *Pagurus* | *ochotensis* | Vostok Bay | 2014 | 42.880832 | 132.742867 | Russia | Asia | Kornienko, E. S., Korn, O. M., & Selin, N. I. (2018). The Parasitic Fauna of Common Species of Hermit Crabs of Vostok Bay (Sea of Japan). Russian Journal of Marine Biology, 44(2), 94–99.<https://doi.org/10.1134/S1063074018020065> |
| 0 | 0 | 144 | *Pagurus* | *middendorffii* | Vostok Bay | 2014 | 42.880832 | 132.742867 | Russia | Asia | Kornienko, E. S., Korn, O. M., & Selin, N. I. (2018). The Parasitic Fauna of Common Species of Hermit Crabs of Vostok Bay (Sea of Japan). Russian Journal of Marine Biology, 44(2), 94–99.<https://doi.org/10.1134/S1063074018020066> |
| 8.5 | 17 | 182 | *Pagurus* | *exilis* | estuary of Bahia Blanca | 2011 | -38.786549 | -62.303054 | Argentina | South_America | Dibo, M., & Tanzola, D. (2017). Parisites and epibonts of the hermit crab Pagurus exilis(Crustacea:Anomura) from the estuary of Bahía Blanca. Neotropical Helminthology, 11(1), 77–84. |
| NA | 1 | NA | *Pagurus* | *bernhardus* | Bembridge | 1963 | 50.690828 | -1.075436 | UK | Europe | (Juniper 1963) in Herbert, R. J. H., & Muxagata, E. (2009). Barnacles (Crustacea: Cirripeidia) of the Solent and Isle of Wight. Proceedings of The Isle of Wight Natural History & Archaeological Society.<https://doi.org/10.1017/CBO9781107415324.004> |
| NA | 2 | NA | *Pagurus* | *prideaux* | Bay of Naples | 1876 | 40.698542 | 14.150914 | Italy | Europe | Øksenbjerg, B. (2000). The Rhizocephala (Crustacea: Cirripedia) of the Mediterraean and Black seas: taxonomy, biogeography, and ecology. Israel Journal of Zoology, 46, 1–102. |
| NA | 1 | NA | *Pagurus* | *excavatus* | NA | 1904 | NA | NA | NA | NA | Øksenbjerg, B. (2000). The Rhizocephala (Crustacea: Cirripedia) of the Mediterraean and Black seas: taxonomy, biogeography, and ecology. Israel Journal of Zoology, 46, 1–102. |
| NA | 87 | NA | *Anapagurus* | *laevis* | Clyde sea | 1970 | 55.633257 | -4.778415 | UK | Europe | Nielsen, S. O. (1970). The effects of the rhizocephalan parasites peltogaster paguri rathke and gemmosaccus sulcatus (lilljeborg) on five species of paguridan hosts (crustacea decapoda). Sarsia, 42(1), 17–32.<https://doi.org/10.1080/00364827.1970.10411160> |
| NA | 50 | NA | *Pagurus* | *bernhardus* | Clyde sea | 1970 | 55.633257 | -4.778415 | UK | Europe | Nielsen, S. O. (1970). The effects of the rhizocephalan parasites peltogaster paguri rathke and gemmosaccus sulcatus (lilljeborg) on five species of paguridan hosts (crustacea decapoda). Sarsia, 42(1), 17–32.<https://doi.org/10.1080/00364827.1970.10411161> |
| NA | 5 | NA | *Pagurus* | *bernhardus* | Skagerrak | 1970 | 58.543059 | 11.239991 | Norway | Europe | Nielsen, S. O. (1970). The effects of the rhizocephalan parasites peltogaster paguri rathke and gemmosaccus sulcatus (lilljeborg) on five species of paguridan hosts (crustacea decapoda). Sarsia, 42(1), 17–32.<https://doi.org/10.1080/00364827.1970.10411162> |
| NA | 4 | NA | *Pagurus* | *bernhardus* | Oresund | 1970 | 55.766423 | 12.730704 | Sweden | Europe | Nielsen, S. O. (1970). The effects of the rhizocephalan parasites peltogaster paguri rathke and gemmosaccus sulcatus (lilljeborg) on five species of paguridan hosts (crustacea decapoda). Sarsia, 42(1), 17–32.<https://doi.org/10.1080/00364827.1970.10411163> |
| NA | 1 | NA | *Pagurus* | *bernhardus* | Espegrend | 1970 | 60.269298 | 5.221351 | Norway | Europe | Nielsen, S. O. (1970). The effects of the rhizocephalan parasites peltogaster paguri rathke and gemmosaccus sulcatus (lilljeborg) on five species of paguridan hosts (crustacea decapoda). Sarsia, 42(1), 17–32.<https://doi.org/10.1080/00364827.1970.10411164> |
| NA | 4 | NA | *Pagurus* | *prideaux* | might be citing another paper- watch for the other paper later, just occurrence, dont know how many | 1970 | 55.633257 | -4.778415 | UK | Europe | Nielsen, S. O. (1970). The effects of the rhizocephalan parasites peltogaster paguri rathke and gemmosaccus sulcatus (lilljeborg) on five species of paguridan hosts (crustacea decapoda). Sarsia, 42(1), 17–32.<https://doi.org/10.1080/00364827.1970.10411165> |
| NA | 2 | NA | *Pagurus* | *prideaux* | Espegrend | 1970 | 60.269298 | 5.221351 | Norway | Europe | Nielsen, S. O. (1970). The effects of the rhizocephalan parasites peltogaster paguri rathke and gemmosaccus sulcatus (lilljeborg) on five species of paguridan hosts (crustacea decapoda). Sarsia, 42(1), 17–32.<https://doi.org/10.1080/00364827.1970.10411166> |
| NA | 2 | NA | *Pagurus* | *pubescens* | Smögen | 1970 | 58.348952 | 11.199081 | Sweden | Europe | Nielsen, S. O. (1970). The effects of the rhizocephalan parasites peltogaster paguri rathke and gemmosaccus sulcatus (lilljeborg) on five species of paguridan hosts (crustacea decapoda). Sarsia, 42(1), 17–32.<https://doi.org/10.1080/00364827.1970.10411167> |
| NA | 2 | NA | *Pagurus* | *pubescens* | Gullmaren mouth | 1970 | 58.249207 | 11.335767 | Sweden | Europe | Nielsen, S. O. (1970). The effects of the rhizocephalan parasites peltogaster paguri rathke and gemmosaccus sulcatus (lilljeborg) on five species of paguridan hosts (crustacea decapoda). Sarsia, 42(1), 17–32.<https://doi.org/10.1080/00364827.1970.10411168> |
| NA | 1 | NA | *Pagurus* | *bernhardus* | Teignmouth Bay | 1906 | 50.545421 | -3.493535 | UK | Europe | Norman, A. M., & Scott, T. (1906). The Crustacea of Devon and Cornwall. Wesley, 226.<https://doi.org/10.1038/075387a0> |
| NA | 1 | NA | *Pagurus* | *bernhardus* | Hilbre Swash | 1885 | 53.416667 | -3.333333 | UK | Europe | Marine Biological Station at Port Erin. (1903). Proceedings and Transactions of the Liverpool Biological Society., 17, 40–41.<http://www.biodiversitylibrary.org/item/129204> |
| NA | 1 | NA | *Pagurus* | *bernhardus* | Clyde sea | 1886 | 55.52664 | -4.923062 | UK | Europe | Elliot, G. F. S., Laurie, M., Murdoch, J. B., & for the Advancement of Science., B. A. (1901). Fauna, flora & geology of the Clyde area,.<http://www.biodiversitylibrary.org/bibliography/1392> |
| NA | 1 | NA | *Pagurus* | *bernhardus* | Saunton Sands | 1906 | 51.091503 | -4.22667 | UK | Europe | Page, W. (1906). A History of Devonshire. The Victoria History of the Counties of England, Devonshire, 1, 276.<https://doi.org/10.5962/bhl.title.70357> |
| NA | 1 | NA | *Pagurus* | *bernhardus* | Bergen | 1859 | 60.39378 | 5.308735 | Norway | Europe | Sars, G. O. (1899). An account of the Crustacea of Norway, with short descriptions and figures of all the species. By G.O. Sars ...<https://doi.org/10.5962/bhl.title.1164> |
| NA | 1 | NA | *Pagurus* | *bernhardus* | Firth of Forth, Joppa | 1881 | 55.949162 | -3.094708 | UK | Europe | Leslie, G., & Herdman, W. H. (1881). The invertebrate fauna of the Firth of Forth. |
| NA | 1 | NA | *Pagurus* | *bernhardus* | East of May Island | 1885 | 56.187167 | -2.528588 | UK | Europe | Scott, T. (1885). A revised list of the crustacea of the Firth of Forth.<https://doi.org/10.5962/bhl.title.53620> |
| NA | 1 | NA | *Pagurus* | NA | East Loch Tarbert | 1897 | 55.865977 | -5.409805 | UK | Europe | Scott, T. (1897). The marine fishes and invertebrates of Loch Fyne. Fifteenth Annual Report of the Fisheries Board for Scotland, 156.<https://doi.org/10.5962/bhl.title.53611> |
| NA | 1 | NA | *Pagurus* | *bernhardus* | station 9 | 1898 | 56.131415 | -2.468179 | UK | Europe | Pearcy, F. G. (1903). Notes on the marine deposits of the Firth of Forth and their relation to its animal life. Transactions of the Natural History Society of Glasgow, 6, 217–251.<http://www.biodiversitylibrary.org/item/44889> |
| NA | 1 | NA | *Pagurus* | *samuelis* | Sunset Bay | 1994 | 43.333307 | -124.373456 | USA | North_America | Torchin, M. E. (1994). The effects of parasitism on the hermocyanin of an intertidal hermit crab. University of Oregon. |
| NA | 1 | NA | *Pagurus* | *samuelis* | Cape Arago | 1994 | 43.30686 | -124.402353 | USA | North_America | Torchin, M. E. (1994). The effects of parasitism on the hermocyanin of an intertidal hermit crab. University of Oregon. |
| NA | 1 | NA | *Pagurus* | *bernhardus* | South Coast of Devon | 1883 | 50.606812 | -3.357863 | UK | Europe | D’Urban, W. S. M. (1884). Crustacea on the South Coast of Devon. The Zoologist : A Monthly Journal of Natural History, ser.3 8(85), 150-152 |
| NA | 1 | NA | NA | NA | Bohuslän | 1859 | 58.319717 | 11.394636 | Sweden | Europe | Lilljeborg, W. (1861). Supplementary Memoir on the Genera Liriope and Peltogaster, Rathke. The Annals and Magazine of Natural History; Zoology, Botany, and Geology, ser.3 7(37), 47–56.<https://doi.org/10.1038/001189d0> |
| NA | 1 | NA | *Pagurus* | *bernhardus* | Off Sunderland | 1864 | 54.916913 | -1.240334 | UK | Europe | Norman, A. M. (1864). Report on the Crustacea. Transactions of the Tyneside Naturalists’ Field Club., 6, 183–187.<http://www.biodiversitylibrary.org/item/40570> |
| NA | 1 | NA | *Pagurus* | *bernhardus* | St. Andrews | 1874 | 56.344539 | -2.790215 | UK | Europe | M’Inctosh, W. M. (1874). Dr. W.C. M'Intosh on the Crustacea of St. Andrews. Annals and Magazine of Natural History; Zoology, Botany, and Geology, ser. 4 14(83), 337–348.<https://doi.org/10.1038/001189d0> |
| NA | 1 | NA | NA | NA | Off Durham | 1863 | 54.805605 | -1.256716 | UK | Europe | Norman, A. M. (1867). Rev. A.M. Norman on the Crustacea. Transactions of the Natural History Society of Northumberland and Durham, 1, 12–29. |
| NA | 1 | NA | *Pagurus* | *bernhardus* | Firth of Forth | 1855 | 56.01569 | -3.357225 | UK | Europe | Anderson, J. (1858). On the Genus Peltogaster(Rathke); an animal form parasitic on the abdomen of crabs. Proceedings of the Royal Physical Society of Edinburgh, 1, 412–414. |
| NA | 1 | NA | NA | NA | Heligoland | 1846 | 54.181868 | 7.879956 | Germany | Europe | Leuckart. (1869). Observations on the genus Sacculina, Thompson (Pachybdella, Diesing; Peltogaster, Rathke). The Annals and Magazine of Natural History; Zoology, Botany, and Geology, ser. 3 4(24), 422–429.<https://doi.org/10.1038/001189d0> |
| NA | 0 | NA | *Pagurus* | *NA* | Firth of Forth | 1861 | 56.08197 | -2.973264 | UK | Europe | Logan, G. (1863). Report of the Committee on Marine Zoology; with a Notice of the Sprat Fishing in the Firth of Forth. Proceedings of the Royal Physical Society of Edinburgh, 2, 240–243. |
| NA | 1 | NA | NA | NA | St. Andrews | 2016 | 56.33618 | -2.77715 | UK | Europe | [GBIF.org](http://gbif.org) (17 June 2020) GBIF Occurrence Download<https://doi.org/10.15468/dl.5btacz> |
| NA | 1 | NA | NA | NA | KBN02 | 2014 | 51.331 | 2.681 | Belgium | Europe | [GBIF.org](http://gbif.org) (17 June 2020) GBIF Occurrence Download<https://doi.org/10.15468/dl.5btacz> |
| NA | 1 | NA | NA | NA | North sea | 2013 | 55.459 | 5.245 | Denmark | Europe | [GBIF.org](http://gbif.org) (17 June 2020) GBIF Occurrence Download<https://doi.org/10.15468/dl.5btacz> |
| NA | 1 | NA | NA | NA | North sea | 2013 | 60.37 | 2.478 | Norway | Europe | [GBIF.org](http://gbif.org) (17 June 2020) GBIF Occurrence Download<https://doi.org/10.15468/dl.5btacz> |
| NA | 1 | NA | NA | NA | NA | 2012 | 58.744 | 2.564 | Norway | Europe | [GBIF.org](http://gbif.org) (17 June 2020) GBIF Occurrence Download<https://doi.org/10.15468/dl.5btacz> |
| NA | 1 | NA | NA | NA | Morgat | 2010 | 48.227223 | -4.497777 | France | Europe | [GBIF.org](http://gbif.org) (17 June 2020) GBIF Occurrence Download<https://doi.org/10.15468/dl.5btacz> |
| NA | 1 | NA | NA | NA | LNP.09 | 2009 | 51.2414 | 2.7219 | Belgium | Europe | [GBIF.org](http://gbif.org) (17 June 2020) GBIF Occurrence Download<https://doi.org/10.15468/dl.5btacz> |
| NA | 2 | NA | NA | NA | 1-TD.04 | 2005 | 52.94655 | -5.89449 | Ireland | Europe | [GBIF.org](http://gbif.org) (17 June 2020) GBIF Occurrence Download<https://doi.org/10.15468/dl.5btacz> |
| NA | 1 | NA | NA | NA | Heda | 2003 | 34.970831 | 138.762045 | Japan | Asia | [GBIF.org](http://gbif.org) (17 June 2020) GBIF Occurrence Download<https://doi.org/10.15468/dl.5btacz> |
| NA | 1 | NA | NA | NA | Misaki | 2002 | 35.140123 | 139.613323 | Japan | Asia | [GBIF.org](http://gbif.org) (17 June 2020) GBIF Occurrence Download<https://doi.org/10.15468/dl.5btacz> |
| NA | 1 | NA | NA | NA | Misaki | 2001 | 35.140123 | 139.613323 | Japan | Asia | [GBIF.org](http://gbif.org) (17 June 2020) GBIF Occurrence Download<https://doi.org/10.15468/dl.5btacz> |
| NA | 1 | NA | NA | NA | Amatsukominato | 2001 | 35.119643 | 140.157615 | Japan | Asia | [GBIF.org](http://gbif.org) (17 June 2020) GBIF Occurrence Download<https://doi.org/10.15468/dl.5btacz> |
| NA | 1 | NA | NA | NA | Amakusa | 2000 | 32.246512 | 129.972367 | Japan | Asia | [GBIF.org](http://gbif.org) (17 June 2020) GBIF Occurrence Download<https://doi.org/10.15468/dl.5btacz> |
| NA | 1 | NA | NA | NA | Sanriku | 1996 | 39.972204 | 141.953746 | Japan | Asia | [GBIF.org](http://gbif.org) (17 June 2020) GBIF Occurrence Download<https://doi.org/10.15468/dl.5btacz> |
| NA | 1 | NA | *Pagurus* | *bernardus* | Rotterdam | 1993 | 51.98 | 4.13 | Netherlands | Europe | [GBIF.org](http://gbif.org) (17 June 2020) GBIF Occurrence Download<https://doi.org/10.15468/dl.5btacz> |
| NA | 1 | NA | NA | NA | Hallangstangen | 1993 | 59.681495 | 10.616962 | Norway | Europe | [GBIF.org](http://gbif.org) (17 June 2020) GBIF Occurrence Download<https://doi.org/10.15468/dl.5btacz> |
| NA | 1 | NA | NA | NA | Aber to Gorad y Gyt (Menai Strait) | 1992 | 53.23736 | -4.12239 | UK | Europe | [GBIF.org](http://gbif.org) (17 June 2020) GBIF Occurrence Download<https://doi.org/10.15468/dl.5btacz> |
| NA | 1 | NA | NA | NA | Church Island to Pwll Fanogl (Menai Strait) | 1992 | 53.22299 | -4.17112 | UK | Europe | [GBIF.org](http://gbif.org) (17 June 2020) GBIF Occurrence Download<https://doi.org/10.15468/dl.5btacz> |
| NA | 1 | NA | NA | NA | North sea | 1989 | 55.27328 | -1.340022 | UK | Europe | [GBIF.org](http://gbif.org) (17 June 2020) GBIF Occurrence Download<https://doi.org/10.15468/dl.5btacz> |
| NA | 1 | NA | NA | NA | Puzzlehole | 1981 | 54.433333 | 4.416667 | UK | Europe | [GBIF.org](http://gbif.org) (17 June 2020) GBIF Occurrence Download<https://doi.org/10.15468/dl.5btacz> |
| NA | 1 | NA | NA | NA | North sea | 1980 | 52.633333 | 5.35 | Netherlands | Europe | [GBIF.org](http://gbif.org) (17 June 2020) GBIF Occurrence Download<https://doi.org/10.15468/dl.5btacz> |
| NA | 1 | NA | NA | NA | st. 78.03 | 1978 | 69.616667 | 18.916667 | Norway | Europe | [GBIF.org](http://gbif.org) (17 June 2020) GBIF Occurrence Download<https://doi.org/10.15468/dl.5btacz> |
| NA | 1 | NA | NA | NA | North sea | 1976 | 56.510671 | 3.515545 | Netherlands | Europe | [GBIF.org](http://gbif.org) (17 June 2020) GBIF Occurrence Download<https://doi.org/10.15468/dl.5btacz> |
| NA | 1 | NA | NA | NA | Traeth Bychan | 1974 | 53.339552 | -4.230409 | UK | Europe | [GBIF.org](http://gbif.org) (17 June 2020) GBIF Occurrence Download<https://doi.org/10.15468/dl.5btacz> |
| NA | 1 | NA | NA | NA | North sea | 1971 | 53.183333 | 4.25 | Netherlands | Europe | [GBIF.org](http://gbif.org) (17 June 2020) GBIF Occurrence Download<https://doi.org/10.15468/dl.5btacz> |
| NA | 1 | NA | NA | NA | Stångehuvuds | 1971 | 58.266667 | 11.416667 | Sweden | Europe | [GBIF.org](http://gbif.org) (17 June 2020) GBIF Occurrence Download<https://doi.org/10.15468/dl.5btacz> |
| NA | 1 | NA | NA | NA | off Whitby | 1971 | 54.519151 | -0.590839 | UK | Europe | [GBIF.org](http://gbif.org) (17 June 2020) GBIF Occurrence Download<https://doi.org/10.15468/dl.5btacz> |
| NA | 1 | NA | NA | NA | Gåsö | 1967 | 58.233333 | 11.416667 | Sweden | Europe | [GBIF.org](http://gbif.org) (17 June 2020) GBIF Occurrence Download<https://doi.org/10.15468/dl.5btacz> |
| NA | 1 | NA | NA | NA | Aber, St. Efflan en Penpoull | 1965 | 48.66927 | -3.604772 | France | Europe | [GBIF.org](http://gbif.org) (17 June 2020) GBIF Occurrence Download<https://doi.org/10.15468/dl.5btacz> |
| NA | 2 | NA | NA | NA | Penpoull | 1965 | 47.571844 | -3.01073 | France | Europe | [GBIF.org](http://gbif.org) (17 June 2020) GBIF Occurrence Download<https://doi.org/10.15468/dl.5btacz> |
| NA | 2 | NA | *Diogenes* | *pugilator* | St. Efflan | 1965 | 48.669854 | -3.593134 | France | Europe | [GBIF.org](http://gbif.org) (17 June 2020) GBIF Occurrence Download<https://doi.org/10.15468/dl.5btacz> |
| NA | 3 | NA | NA | NA | St. Efflan | 1965 | 48.669854 | -3.593134 | France | Europe | [GBIF.org](http://gbif.org) (17 June 2020) GBIF Occurrence Download<https://doi.org/10.15468/dl.5btacz> |
| NA | 1 | NA | NA | NA | Gann Flat | 1960 | 51.71491 | -5.16803 | UK | Europe | [GBIF.org](http://gbif.org) (17 June 2020) GBIF Occurrence Download<https://doi.org/10.15468/dl.5btacz> |
| NA | 1 | NA | NA | NA | Dale Roads | 1960 | 51.71323 | -5.16357 | UK | Europe | [GBIF.org](http://gbif.org) (17 June 2020) GBIF Occurrence Download<https://doi.org/10.15468/dl.5btacz> |
| NA | 1 | NA | NA | NA | Slip Pier Beach | 1960 | 51.70453 | -5.15283 | UK | Europe | [GBIF.org](http://gbif.org) (17 June 2020) GBIF Occurrence Download<https://doi.org/10.15468/dl.5btacz> |
| NA | 1 | NA | NA | NA | Kitterland | 1952 | 54.15463 | -4.88589 | Isle of Man | Europe | [GBIF.org](http://gbif.org) (17 June 2020) GBIF Occurrence Download<https://doi.org/10.15468/dl.5btacz> |
| NA | 3 | NA | NA | NA | Kitterland | 1952 | 54.16162 | -4.89404 | Isle of Man | Europe | [GBIF.org](http://gbif.org) (17 June 2020) GBIF Occurrence Download<https://doi.org/10.15468/dl.5btacz> |
| NA | 11 | NA | NA | NA | Bradda Head | 1950 | 54.14944 | -4.91463 | Isle of Man | Europe | [GBIF.org](http://gbif.org) (17 June 2020) GBIF Occurrence Download<https://doi.org/10.15468/dl.5btacz> |
| NA | 1 | NA | *Pagurus* | *pubescens* | Labrador, Off Coast | 1950 | 54.0217 | -58.695 | Canada | North_America | [GBIF.org](http://gbif.org) (17 June 2020) GBIF Occurrence Download<https://doi.org/10.15468/dl.5btacz> |
| NA | 1 | NA | *Pagurus* | *hirsutiusculus* | Fishermen's Bay | 1950 | 48.510297 | -122.914161 | USA | North_America | [GBIF.org](http://gbif.org) (17 June 2020) GBIF Occurrence Download<https://doi.org/10.15468/dl.5btacz> |
| NA | 1 | NA | *Pagurus* | *pubescens* | Seven Islands Bay | 1950 | 59.39 | -64.0533 | Canada | North_America | [GBIF.org](http://gbif.org) (17 June 2020) GBIF Occurrence Download<https://doi.org/10.15468/dl.5btacz> |
| NA | 3 | NA | *Pagurus* | *pubescens* | Labrador, Off Coast | 1950 | 59.4 | -63.85 |  | North_America | [GBIF.org](http://gbif.org) (17 June 2020) GBIF Occurrence Download<https://doi.org/10.15468/dl.5btacz> |
| NA | 3 | NA | *Pagurus* | *pubescens* | Kongulaksiarvik | 1950 | 59.4 | -64.02 |  | North_America | [GBIF.org](http://gbif.org) (17 June 2020) GBIF Occurrence Download<https://doi.org/10.15468/dl.5btacz> |
| NA | 1 | NA | NA | NA | Scheveningen | 1949 | 52.112635 | 4.275621 | Netherlands | Europe | [GBIF.org](http://gbif.org) (17 June 2020) GBIF Occurrence Download<https://doi.org/10.15468/dl.5btacz> |
| NA | 1 | NA | NA | NA | Cross Houses, Millport, Firth of Clyde | 1949 | 55.752136 | -4.927219 | UK | Europe | [GBIF.org](http://gbif.org) (17 June 2020) GBIF Occurrence Download<https://doi.org/10.15468/dl.5btacz> |
| NA | 1 | NA | NA | NA | Katwijk, paal 86 | 1949 | 52.200823 | 4.387546 | Netherlands | Europe | [GBIF.org](http://gbif.org) (17 June 2020) GBIF Occurrence Download<https://doi.org/10.15468/dl.5btacz> |
| NA | 1 | NA | NA | NA | Scheveningen, paal 99 | 1949 | 52.112635 | 4.275621 | Netherlands | Europe | [GBIF.org](http://gbif.org) (17 June 2020) GBIF Occurrence Download<https://doi.org/10.15468/dl.5btacz> |
| NA | 1 | NA | NA | NA | Yorkshire | 1949 | 54.203849 | -0.160697 | UK | Europe | [GBIF.org](http://gbif.org) (17 June 2020) GBIF Occurrence Download<https://doi.org/10.15468/dl.5btacz> |
| NA | 1 | NA | NA | NA | Jersey | 1949 | 49.253706 | -2.133759 | Jersey | Europe | [GBIF.org](http://gbif.org) (17 June 2020) GBIF Occurrence Download<https://doi.org/10.15468/dl.5btacz> |
| NA | 1 | NA | NA | NA | Cross House, Millport, Cumbrae | 1949 | 55.752136 | -4.927219 | UK | Europe | [GBIF.org](http://gbif.org) (17 June 2020) GBIF Occurrence Download<https://doi.org/10.15468/dl.5btacz> |
| NA | 1 | NA | NA | NA | Cross House, Millport, Cumbrae | 1949 | 55.752136 | -4.927219 | UK | Europe | [GBIF.org](http://gbif.org) (17 June 2020) GBIF Occurrence Download<https://doi.org/10.15468/dl.5btacz> |
| NA | 1 | NA | NA | NA | Yorkshire | 1949 | 54.203849 | -0.160697 | UK | Europe | [GBIF.org](http://gbif.org) (17 June 2020) GBIF Occurrence Download<https://doi.org/10.15468/dl.5btacz> |
| NA | 1 | NA | NA | NA | Kattegatt off Falkenberg | 1948 | 56.846054 | 12.213912 | UK | Europe | [GBIF.org](http://gbif.org) (17 June 2020) GBIF Occurrence Download<https://doi.org/10.15468/dl.5btacz> |
| NA | 1 | NA | NA | NA | √ñresund, Svinb√•dan E | 1948 | 56.148785 | 12.499002 | UK | Europe | [GBIF.org](http://gbif.org) (17 June 2020) GBIF Occurrence Download<https://doi.org/10.15468/dl.5btacz> |
| NA | 1 | NA | NA | NA | Anglesey | 1938 | 53.33356 | -4.17223 | UK | Europe | [GBIF.org](http://gbif.org) (17 June 2020) GBIF Occurrence Download<https://doi.org/10.15468/dl.5btacz> |
| NA | 1 | NA | NA | NA | Anglesey | 1938 | 53.33356 | -4.17223 | UK | Europe | [GBIF.org](http://gbif.org) (17 June 2020) GBIF Occurrence Download<https://doi.org/10.15468/dl.5btacz> |
| NA | 1 | NA | NA | NA | West Den Helder | 1935 | 52.963204 | 4.735406 | Netherlands | Europe | [GBIF.org](http://gbif.org) (17 June 2020) GBIF Occurrence Download<https://doi.org/10.15468/dl.5btacz> |
| NA | 1 | NA | NA | NA | Naples | 1930 | 40.831819 | 14.235735 | Italy | Europe | [GBIF.org](http://gbif.org) (17 June 2020) GBIF Occurrence Download<https://doi.org/10.15468/dl.5btacz> |
| NA | 1 | NA | NA | NA | St Jacut de la Mer | 1922 | 48.598845 | -2.195552 | France | Europe | [GBIF.org](http://gbif.org) (17 June 2020) GBIF Occurrence Download<https://doi.org/10.15468/dl.5btacz> |
| NA | 1 | NA | NA | NA | Gullmaren | 1921 | 58.343469 | 11.566176 | Sweden | Europe | [GBIF.org](http://gbif.org) (17 June 2020) GBIF Occurrence Download<https://doi.org/10.15468/dl.5btacz> |
| NA | 1 | NA | NA | NA | Oresund | 1913 | 55.771032 | 12.746697 | Sweden | Europe | [GBIF.org](http://gbif.org) (17 June 2020) GBIF Occurrence Download<https://doi.org/10.15468/dl.5btacz> |
| NA | 1 | NA | *Pagurus* | *cuanensis* | Belgium | 1912 | 51.256473 | 2.935267 | Belgium | Europe | [GBIF.org](http://gbif.org) (17 June 2020) GBIF Occurrence Download<https://doi.org/10.15468/dl.5btacz> |
| NA | 1 | NA | *Pagurus* | *bernardus* | Belgium | 1908 | 51.256473 | 2.935267 | Belgium | Europe | [GBIF.org](http://gbif.org) (17 June 2020) GBIF Occurrence Download<https://doi.org/10.15468/dl.5btacz> |
| NA | 1 | NA | *Pagurus* | *bernardus* | Belgium | 1907 | 51.256473 | 2.935267 | Belgium | Europe | [GBIF.org](http://gbif.org) (17 June 2020) GBIF Occurrence Download<https://doi.org/10.15468/dl.5btacz> |
| NA | 1 | NA | *Pagurus* | *bernardus* | Belgium | 1907 | 51.256473 | 2.935267 | Belgium | Europe | [GBIF.org](http://gbif.org) (17 June 2020) GBIF Occurrence Download<https://doi.org/10.15468/dl.5btacz> |
| NA | 1 | NA | *Pagurus* | *bernardus* | Belgium | 1907 | 51.256473 | 2.935267 | Belgium | Europe | [GBIF.org](http://gbif.org) (17 June 2020) GBIF Occurrence Download<https://doi.org/10.15468/dl.5btacz> |
| NA | 1 | NA | *Pagurus* | *bernardus* | Belgium | 1907 | 51.256473 | 2.935267 | Belgium | Europe | [GBIF.org](http://gbif.org) (17 June 2020) GBIF Occurrence Download<https://doi.org/10.15468/dl.5btacz> |
| NA | 1 | NA | NA | NA | Belgium | 1907 | 51.256473 | 2.935267 | Belgium | Europe | [GBIF.org](http://gbif.org) (17 June 2020) GBIF Occurrence Download<https://doi.org/10.15468/dl.5btacz> |
| NA | 1 | NA | *Pagurus* | *bernardus* | Belgium | 1907 | 51.256473 | 2.935267 | Belgium | Europe | [GBIF.org](http://gbif.org) (17 June 2020) GBIF Occurrence Download<https://doi.org/10.15468/dl.5btacz> |
| NA | 1 | NA | NA | NA | Belgium | 1906 | 51.256473 | 2.935267 | Belgium | Europe | [GBIF.org](http://gbif.org) (17 June 2020) GBIF Occurrence Download<https://doi.org/10.15468/dl.5btacz> |
| NA | 1 | NA | *Pagurus* | *bernhardus* | Belgium | 1905 | 51.256473 | 2.935267 | Belgium | Europe | [GBIF.org](http://gbif.org) (17 June 2020) GBIF Occurrence Download<https://doi.org/10.15468/dl.5btacz> |
| NA | 1 | NA | *Pagurus* | *cuanensis* | Belgium | 1905 | 51.256473 | 2.935267 | Belgium | Europe | [GBIF.org](http://gbif.org) (17 June 2020) GBIF Occurrence Download<https://doi.org/10.15468/dl.5btacz> |
| NA | 2 | NA | *Pagurus* | *bernhardus* | Belgium | 1903 | 51.256473 | 2.935267 | Belgium | Europe | [GBIF.org](http://gbif.org) (17 June 2020) GBIF Occurrence Download<https://doi.org/10.15468/dl.5btacz> |
| NA | 1 | NA | *Pagurus* | *hirsutiusculus* | Virgin Bay, Prince William Sound | 1899 | 60.891833 | -146.702279 | USA | North_America | [GBIF.org](http://gbif.org) (17 June 2020) GBIF Occurrence Download<https://doi.org/10.15468/dl.5btacz> |
| NA | 1 | NA | *Pagurus* | *kennerlyi* | Juneau | 1899 | 58.351336 | -134.616507 | USA | North_America | [GBIF.org](http://gbif.org) (17 June 2020) GBIF Occurrence Download<https://doi.org/10.15468/dl.5btacz> |
| NA | 1 | NA | NA | NA | Pribilof Islands, East of Islands | 1895 | 56.75 | -167.417 | USA | North_America | [GBIF.org](http://gbif.org) (17 June 2020) GBIF Occurrence Download<https://doi.org/10.15468/dl.5btacz> |
| NA | 2 | NA | *Pagurus* | *trigonocheirus* | Pribilof Islands, North of Islands | 1893 | 58.45 | -169.017 | USA | North_America | [GBIF.org](http://gbif.org) (17 June 2020) GBIF Occurrence Download<https://doi.org/10.15468/dl.5btacz> |
| NA | 1 | NA | *Pagurus* | *capillatus* | Unimak Pass | 1890 | 54.8 | -165.225 | USA | North_America | [GBIF.org](http://gbif.org) (17 June 2020) GBIF Occurrence Download<https://doi.org/10.15468/dl.5btacz> |
| NA | 1 | NA | *Anapagurus* | *chiroacanthus* | Bohuslän N. Gåsörännan | 1890 | 58.235742 | 11.419524 | Sweden | Europe | [GBIF.org](http://gbif.org) (17 June 2020) GBIF Occurrence Download<https://doi.org/10.15468/dl.5btacz> |
| NA | 1 | NA | NA | NA | Barents sea | 1889 | 74.826351 | 37.068095 | Russia | Asia | [GBIF.org](http://gbif.org) (17 June 2020) GBIF Occurrence Download<https://doi.org/10.15468/dl.5btacz> |
| NA | 3 | NA | *Pagurus* | *pubescens* | Grand Banks | 1885 | 46.1583 | -49.8083 | Canada | North_America | [GBIF.org](http://gbif.org) (17 June 2020) GBIF Occurrence Download<https://doi.org/10.15468/dl.5btacz> |
| NA | 2 | NA | *Pagurus* | *pubescens* | Grand Banks, South of | 1885 | 43.6333 | -49.825 | Canada | North_America | [GBIF.org](http://gbif.org) (17 June 2020) GBIF Occurrence Download<https://doi.org/10.15468/dl.5btacz> |
| NA | 1 | NA | NA | NA | Starcross | 1883 | 50.627155 | -3.447025 | UK | Europe | [GBIF.org](http://gbif.org) (17 June 2020) GBIF Occurrence Download<https://doi.org/10.15468/dl.5btacz> |
| NA | 1 | NA | *Pagurus* | *arcuatus* | Cape Cod, off Nausett Lights | 1882 | 41.86396 | -69.880939 | USA | North_America | [GBIF.org](http://gbif.org) (17 June 2020) GBIF Occurrence Download<https://doi.org/10.15468/dl.5btacz> |
| NA | 1 | NA | NA | NA | Cape Cod, off Nausett Lights | 1882 | 41.86396 | -69.880939 | USA | North_America | [GBIF.org](http://gbif.org) (17 June 2020) GBIF Occurrence Download<https://doi.org/10.15468/dl.5btacz> |
| NA | 3 | NA | *Pagurus* | *trigonocheirus* | Cape Smyth | 1882 | 71.293701 | -156.789627 | USA | North_America | [GBIF.org](http://gbif.org) (17 June 2020) GBIF Occurrence Download<https://doi.org/10.15468/dl.5btacz> |
| NA | 1 | NA | *Pagurus* | *cuanensis* | Bohuslan Gullmaren | 1882 | 58.343134 | 11.566722 | Sweden | Europe | [GBIF.org](http://gbif.org) (17 June 2020) GBIF Occurrence Download<https://doi.org/10.15468/dl.5btacz> |
| NA | 1 | NA | *Pagurus* | *pubescens* | Bohuslän Wäderöarna | 1882 | 58.571838 | 11.075711 | Sweden | Europe | [GBIF.org](http://gbif.org) (17 June 2020) GBIF Occurrence Download<https://doi.org/10.15468/dl.5btacz> |
| NA | 1 | NA | *Pagurus* | *pubescens* | Crab Ledge, off Chatham Lights | 1881 | 41.665919 | -69.905198 | USA | North_America | [GBIF.org](http://gbif.org) (17 June 2020) GBIF Occurrence Download<https://doi.org/10.15468/dl.5btacz> |
| NA | 1 | NA | NA | NA | Martha's Vineyard | 1881 | 40.9 | -70.8083 | USA | North_America | [GBIF.org](http://gbif.org) (17 June 2020) GBIF Occurrence Download<https://doi.org/10.15468/dl.5btacz> |
| NA | 3 | NA | NA | NA | Martha's Vineyard | 1881 | 40.92 | -70.8 | USA | North_America | [GBIF.org](http://gbif.org) (17 June 2020) GBIF Occurrence Download<https://doi.org/10.15468/dl.5btacz> |
| NA | 2 | NA | *Pagurus* | *pubescens* | Cape Cod Bay, mouth of | 1879 | 42.0458 | -70.48 | USA | North_America | [GBIF.org](http://gbif.org) (17 June 2020) GBIF Occurrence Download<https://doi.org/10.15468/dl.5btacz> |
| NA | 1 | NA | NA | NA | Gloucester Harbor, off Cape Ann | 1878 | 42.63 | -70.475 | USA | North_America | [GBIF.org](http://gbif.org) (17 June 2020) GBIF Occurrence Download<https://doi.org/10.15468/dl.5btacz> |
| NA | 3 | NA | NA | NA | Massachusetts Bay, off Thatcher's Island Light and Eastern Point | 1878 | 42.4 | -70.5583 | USA | North_America | [GBIF.org](http://gbif.org) (17 June 2020) GBIF Occurrence Download<https://doi.org/10.15468/dl.5btacz> |
| NA | 1 | NA | *Pagurus* | *pubescens* | Gloucester Harbor, off Thatcher's Island and Eastern Point | 1878 | 42.58 | -70.5 | USA | North_America | [GBIF.org](http://gbif.org) (17 June 2020) GBIF Occurrence Download<https://doi.org/10.15468/dl.5btacz> |
| NA | 1 | NA | NA | NA | Massachusetts Bay, Off Halfway Rock And Eastern Point Light | 1877 | 42.4583 | -70.6 | USA | North_America | [GBIF.org](http://gbif.org) (17 June 2020) GBIF Occurrence Download<https://doi.org/10.15468/dl.5btacz> |
| NA | 1 | NA | *Pagurus* | *capillatus* | Aleutian Islands, Fox Islands, Unalaska Island, Captains Harbor, Outside Of A Ridge | 1874 | 53.847778 | -166.578407 | USA | North_America | [GBIF.org](http://gbif.org) (17 June 2020) GBIF Occurrence Download<https://doi.org/10.15468/dl.5btacz> |
| NA | 1 | NA | *Pagurus* | *kennerlyi* | Alaska Peninsula, Chirikof Island | 1874 | 55.83895 | -155.565061 | USA | North_America | [GBIF.org](http://gbif.org) (17 June 2020) GBIF Occurrence Download<https://doi.org/10.15468/dl.5btacz> |
| NA | 1 | NA | *Pagurus* | *beringanus* | Port Etches, Prince William Sound | 1874 | 60.333782 | -146.581943 | USA | North_America | [GBIF.org](http://gbif.org) (17 June 2020) GBIF Occurrence Download<https://doi.org/10.15468/dl.5btacz> |
| NA | 1 | NA | *Pagurus* | *pubescens* | Casco Bay | 1873 | 43.63 | -70.05 | USA | North_America | [GBIF.org](http://gbif.org) (17 June 2020) GBIF Occurrence Download<https://doi.org/10.15468/dl.5btacz> |
| NA | 1 | NA | NA | NA | Firth of Forth | 1862 | 56.028713 | -3.257395 | UK | Europe | [GBIF.org](http://gbif.org) (17 June 2020) GBIF Occurrence Download<https://doi.org/10.15468/dl.5btacz> |
| NA | 2 | NA | NA | NA | Naples | NA | 40.831948 | 14.234372 | Italy | Europe | [GBIF.org](http://gbif.org) (17 June 2020) GBIF Occurrence Download<https://doi.org/10.15468/dl.5btacz> |
| NA | 1 | NA | NA | NA | st. 150 | NA | 58.216667 | 11.416667 | Sweden | Europe | [GBIF.org](http://gbif.org) (17 June 2020) GBIF Occurrence Download<https://doi.org/10.15468/dl.5btacz> |
| NA | 1 | NA | NA | NA | Southern North Sea | NA | 54.283874 | 4.022422 | NA |  | [GBIF.org](http://gbif.org) (17 June 2020) GBIF Occurrence Download<https://doi.org/10.15468/dl.5btacz> |
| NA | 1 | NA | NA | NA | Southern North Sea | NA | 54.283874 | 4.022422 | NA |  | [GBIF.org](http://gbif.org) (17 June 2020) GBIF Occurrence Download<https://doi.org/10.15468/dl.5btacz> |
| NA | 1 | NA | NA | NA | Southern North Sea | NA | 54.283874 | 4.022422 | NA |  | [GBIF.org](http://gbif.org) (17 June 2020) GBIF Occurrence Download<https://doi.org/10.15468/dl.5btacz> |
| NA | 3 | NA | NA | NA | Eysturoy | NA | 62.05 | -6.25 | Faroe Islands | Europe | [GBIF.org](http://gbif.org) (17 June 2020) GBIF Occurrence Download<https://doi.org/10.15468/dl.5btacz> |
| NA | 1 | NA | NA | NA | st. 133 | NA | 58.25 | 11.45 | Sweden | Europe | [GBIF.org](http://gbif.org) (17 June 2020) GBIF Occurrence Download<https://doi.org/10.15468/dl.5btacz> |
| NA | 1 | NA | NA | NA | North sea | NA | 56.336765 | 3.58607 | NA |  | [GBIF.org](http://gbif.org) (17 June 2020) GBIF Occurrence Download<https://doi.org/10.15468/dl.5btacz> |
| NA | 1 | NA | NA | NA | North sea | NA | 56.336765 | 3.58607 | NA |  | [GBIF.org](http://gbif.org) (17 June 2020) GBIF Occurrence Download<https://doi.org/10.15468/dl.5btacz> |
| NA | 1 | NA | NA | NA | st. 149 | NA | 58.233333 | 11.416667 | NA |  | [GBIF.org](http://gbif.org) (17 June 2020) GBIF Occurrence Download<https://doi.org/10.15468/dl.5btacz> |
| NA | 1 | NA | NA | NA | North sea | NA | 56.336765 | 3.58607 | NA |  | [GBIF.org](http://gbif.org) (17 June 2020) GBIF Occurrence Download<https://doi.org/10.15468/dl.5btacz> |
| NA | 1 | NA | NA | NA | North sea | NA | 56.336765 | 3.58607 | NA |  | [GBIF.org](http://gbif.org) (17 June 2020) GBIF Occurrence Download<https://doi.org/10.15468/dl.5btacz> |
| NA | 1 | NA | NA | NA | North sea | NA | 56.336765 | 3.58607 | NA |  | [GBIF.org](http://gbif.org) (17 June 2020) GBIF Occurrence Download<https://doi.org/10.15468/dl.5btacz> |
| NA | 1 | NA | NA | NA | North sea | NA | 56.336765 | 3.58607 | NA |  | [GBIF.org](http://gbif.org) (17 June 2020) GBIF Occurrence Download<https://doi.org/10.15468/dl.5btacz> |
| NA | 1 | NA | NA | NA | North sea | NA | 56.336765 | 3.58607 | NA |  | [GBIF.org](http://gbif.org) (17 June 2020) GBIF Occurrence Download<https://doi.org/10.15468/dl.5btacz> |
| NA | 1 | NA | NA | NA | st.169 | NA | 58.266667 | 11.416667 | NA |  | [GBIF.org](http://gbif.org) (17 June 2020) GBIF Occurrence Download<https://doi.org/10.15468/dl.5btacz> |
| NA | 1 | NA | NA | NA | North sea | NA | 56.336765 | 3.58607 | NA |  | [GBIF.org](http://gbif.org) (17 June 2020) GBIF Occurrence Download<https://doi.org/10.15468/dl.5btacz> |
| NA | 1 | NA | NA | NA | North sea | NA | 56.336765 | 3.58607 | NA |  | [GBIF.org](http://gbif.org) (17 June 2020) GBIF Occurrence Download<https://doi.org/10.15468/dl.5btacz> |
| NA | 1 | NA | NA | NA | North sea | NA | 56.336765 | 3.58607 | NA |  | [GBIF.org](http://gbif.org) (17 June 2020) GBIF Occurrence Download<https://doi.org/10.15468/dl.5btacz> |
| NA | 1 | NA | *Pagurus* | *bernhardus* | Kristineberg | NA | 59.337305 | 17.997838 | NA |  | [GBIF.org](http://gbif.org) (17 June 2020) GBIF Occurrence Download<https://doi.org/10.15468/dl.5btacz> |
| NA | 1 | NA | NA | NA | Wadden sea | NA | 53.568373 | 6.942594 | Germany | Europe | [GBIF.org](http://gbif.org) (17 June 2020) GBIF Occurrence Download<https://doi.org/10.15468/dl.5btacz> |
| NA | 1 | NA | NA | NA | Belgium | NA | 51.206967 | 2.845761 | Belgium | Europe | [GBIF.org](http://gbif.org) (17 June 2020) GBIF Occurrence Download<https://doi.org/10.15468/dl.5btacz> |
| NA | 2 | NA | *Pagurus* | *hirsuitiusculus* | Aleutian Islands, In Little Pools Left By The Receding Tide | NA | 52.662454 | -168.267115 | USA | North_America | [GBIF.org](http://gbif.org) (17 June 2020) GBIF Occurrence Download<https://doi.org/10.15468/dl.5btacz> |
| NA | 1 | NA | NA | NA | Port Seton, Firth of Forth | NA | 55.972904 | -2.945014 | UK | Europe | [GBIF.org](http://gbif.org) (17 June 2020) GBIF Occurrence Download<https://doi.org/10.15468/dl.5btacz> |
| NA | 1 | NA | NA | NA | St. Jacut de la mer, Bretagne | NA | 48.606457 | -2.187536 | France | Europe | [GBIF.org](http://gbif.org) (17 June 2020) GBIF Occurrence Download<https://doi.org/10.15468/dl.5btacz> |
| NA | 1 | NA | NA | NA | Port Seton, Firth of Forth | NA | 55.972904 | -2.945014 | UK | Europe | [GBIF.org](http://gbif.org) (17 June 2020) GBIF Occurrence Download<https://doi.org/10.15468/dl.5btacz> |
| NA | 1 | NA | NA | NA | Naples | NA | 40.76453 | 14.273097 | Italy | Europe | [GBIF.org](http://gbif.org) (17 June 2020) GBIF Occurrence Download<https://doi.org/10.15468/dl.5btacz> |
| NA | 1 | NA | NA | NA | Laeso | NA | 57.251923 | 11.248752 | Denmark | Europe | [GBIF.org](http://gbif.org) (17 June 2020) GBIF Occurrence Download<https://doi.org/10.15468/dl.5btacz> |
| NA | 1 | NA | NA | NA | Helgoland | NA | 54.183333 | 7.883333 | Germany | Europe | [GBIF.org](http://gbif.org) (17 June 2020) GBIF Occurrence Download<https://doi.org/10.15468/dl.5btacz> |
| NA | 1 | NA | NA | NA | Norway | NA | 59.794276 | 5.09965 | Norway | Europe | [GBIF.org](http://gbif.org) (17 June 2020) GBIF Occurrence Download<https://doi.org/10.15468/dl.5btacz> |
| NA | 1 | NA | NA | NA | Norway | NA | 59.794276 | 5.09965 | Norway | Europe | [GBIF.org](http://gbif.org) (17 June 2020) GBIF Occurrence Download<https://doi.org/10.15468/dl.5btacz> |
| NA | 1 | NA | NA | NA | Mediterranean | NA | 35.443605 | 18.214512 | NA |  | [GBIF.org](http://gbif.org) (17 June 2020) GBIF Occurrence Download<https://doi.org/10.15468/dl.5btacz> |
| NA | 1 | NA | NA | NA | Wimereux | NA | 50.77295 | 1.615055 | France | Europe | Muller, Y. (2004). Faune et flore du littoral du Nord, du Pas-de-Calais et de la Belgique: inventaire. [Coastal fauna and flora of the Nord, Pas-de-Calais and B [...]OBIS (2020) Ocean Biodiversity Information System. Intergovernmental Oceanographic Commission of UNESCO. [www.iobis.org](http://www.iobis.org). Accessed: 2020-06-16 |
| NA | 1 | NA | NA | NA | Cobscook Bay | NA | 44.9095235 | -67.0555366 | USA | North_America | Trott, TJ. (2004). Cobscook Bay inventory: a historical checklist of marine invertebrates spanning 162 years. Northeastern Naturalist. 11, 261-324.OBIS (2020) Ocean Biodiversity Information System. Intergovernmental Oceanographic Commission of UNESCO. [www.iobis.org](http://www.iobis.org). Accessed: 2020-06-16 |
| NA | 1 | NA | NA | NA | North sea | 2012 | 51.42218 | 1.64683 | UK | Europe | Cooper et al. (2017). RSMP Baseline Dataset. Cefas, UK. V1.<https://doi.org/10.14466/CefasDataHub.34OBIS> (2020) Ocean Biodiversity Information System. Intergovernmental Oceanographic Commission of UNESCO. [www.iobis.org](http://www.iobis.org). Accessed: 2020-06-16 |
| NA | 1 | NA | NA | NA | Flemish Banks | NA | 51.28777778 | 2.641111111 | Belgium | Europe | Boschma, H. (1947). The European Rhizocephala in the collection of the Brussels Museum of Natural History. <i>Bull. Mus. royal d'Hist. Nat. Belg./Med. Kon. Natu [...]OBIS (2020) Ocean Biodiversity Information System. Intergovernmental Oceanographic Commission of UNESCO. [www.iobis.org](http://www.iobis.org). Accessed: 2020-06-16 |
| NA | 1 | NA | NA | NA | NE Atlantic | NA | 46 | -20 | NA |  | Hayward, P.J.; Ryland, J.S. (Ed.) (1990). The marine fauna of the British Isles and North-West Europe: 1. Introduction and protozoans to arthropods. Clarendon P [...]OBIS (2020) Ocean Biodiversity Information System. Intergovernmental Oceanographic Commission of UNESCO. [www.iobis.org](http://www.iobis.org). Accessed: 2020-06-16 |
| 0 | 0 | NA | *Pagurus* | *proximus* | Vostok Bay | 2014 | 42.877341 | 132.743056 | Russia | Asia | Kornienko, E. S., Selin, N. I., & Korn, O. M. (2019). Population and reproductive characteristics of the hermit crab Pagurus proximus Komai, 2000 (Decapoda: Anomura: Paguridae) in the northern part of the species range. Journal of the Marine Biological Association of the United Kingdom, 99(1), 101–109.<https://doi.org/10.1017/S0025315417001679> |
| 0 | 0 | 229 | *Pagurus* | *bernhardus* | SW coast | 2011 | 51.782529 | -8.279628 | Ireland | Europe | Lynch, S. A., Darmody, G., Laide, C., Walsh, D., & Culloty, S. C. (2015). A preliminary health survey of the hermit crab, Pagurus bernhardus, on the southwest coast of Ireland. Journal of Invertebrate Pathology, 127, 73–75.<https://doi.org/10.1016/j.jip.2015.03.006> |
| 1.4 | NA | NA | *Pagurus* | *bernhardus?* | Quiberon | 1960 | 47.478692 | -3.120155 | France | Europe | Bourdon (1960) in Adema, J. P. H. M., & Huwae, P. H. M. (1982). New and supplementary records of marine isopoda for the Netherlands and the southern North Sea since 1956, with a note on Peltogaster Paguri (Crustacea, Cirripedia). Zoologische Bijdragen, 28(3), 33–57. |
